# Exercise referral schemes enhanced by self-management strategies to reduce sedentary behaviour and increase physical activity among community-dwelling older adults from four European countries: protocol for the process evaluation of the SITLESS randomised controlled trial

**DOI:** 10.1136/bmjopen-2018-027073

**Published:** 2019-06-14

**Authors:** Laura Coll-Planas, Sergi Blancafort Alias, Mark Tully, Paolo Caserotti, Maria Giné-Garriga, Nicole Blackburn, Mathias Skjødt, Katharina Wirth, Manuela Deidda, Emma McIntosh, Dietrich Rothenbacher, Rodrigo Gallardo Rodríguez, Javier Jerez-Roig, Oriol Sansano-Nadal, Marta Santiago, Jason Wilson, Míriam Guerra-Balic, Carme Martín-Borràs, Denise Gonzalez, Guillaume Lefebvre, Michael Denkinger, Frank Kee, Antoni Salvà Casanovas, Marta Roqué i Figuls

**Affiliations:** 1 Fundació Salut i Envelliment (Foundation on Health and Ageing)- UAB, Universitat Autònoma de Barcelona, Barcelona, Spain; 2 Institute of Biomedical Research (IIB Sant Pau), Barcelona, Spain; 3 Institute of Mental Health Sciences, School of Health Sciences, Ulster University, Newtownabbey, UK; 4 UKCRC Centre of Excellence for Public Health (NI), Centre for Public Health, Queen’s University Belfast School of Medicine Dentistry and Biomedical Sciences, Belfast, UK; 5 Department of Sports Science and Clinical Biomechanics, Center for Active and Healthy Ageing (CAHA), Syddansk Universitet, Odense, Denmark; 6 School of Health and Life Sciences, Glasgow Caledonian University, Glasgow, UK; 7 Department of Physical Activity and Sport Sciences, Faculty of Psychology, Education and Sport Sciences (FPCEE) Blanquerna, Ramon Llull University, Barcelona, Spain; 8 Institute of Epidemiology and Medical Biometry, Universitat Ulm, Ulm, Germany; 9 Agaplesion Bethesda Clinic, Geriatric Research Unit Ulm University and Geriatric Center Ulm Alb-Donau, Universitat Ulm, Ulm, Germany; 10 Health Economics and Health Technology Assessment (HEHTA), University of Glasgow Institute of Health and Wellbeing, Glasgow, UK; 11 Departamento de Ciencias del Deporte y Acondicionamiento Físico, CEADE, Universidad Católica de la Santísima Concepción, Concepción, Chile; 12 Research Group on Methodology, Methods, Models and Outcomes of Health and Social Sciences (M3O), Faculty of Health Science and Welfare, Centre for Health and Social Care Research (CESS), Universitat de Vic - Universitat Central de Catalunya, Catalunya, Spain; 13 Department of Physical Therapy, Faculty of Health Sciences (FCS) Blanquerna, Ramon Llull University, Barcelona, Spain; 14 Sport Initiative et Loisir Bleu Association, Strasbourg, France

**Keywords:** epidemiology, public health, geriatric medicine

## Abstract

**Introduction:**

SITLESS is a randomised controlled trial determining whether exercise referral schemes can be enhanced by self-management strategies to reduce sedentary behaviour and increase physical activity in the long term, in community-dwelling older citizens. The intervention is complex and requires a process evaluation to understand how implementation, causal mechanisms and context shape outcomes. The specific aims are to assess fidelity and reach of the implementation, understand the contextual aspects of each intervention site, evaluate the mechanisms of impact, and explore perceived effects.

**Methods and analysis:**

Following the Medical Research Council guidance on complex interventions, a combination of qualitative and quantitative procedures is applied, including observational checklists and attendance registries, standardised scales (ie, Marcus’s Self-Efficacy Questionnaire, Physical Activity Self-Regulation Scale and the Lubben Social Network Scale) at baseline, postintervention and follow-up assessments, semistructured questionnaires gathering contextual characteristics, and participant observations of the sessions. Semistructured interviews and focus groups with the participants and trainers are conducted at postintervention and during the follow-up to explore their experiences. Outcomes from the standardised scales are analysed as moderators within the impact evaluation. Descriptive results on context and perceived effects complement results on impact. The qualitative and quantitative findings will help to refine the logic model to finally support the interpretation of the results on the effectiveness of the intervention.

**Ethics and dissemination:**

The study design was approved by the respective Ethical Committee of Ramon Llull University, Southern Denmark, Northern Ireland and Ulm University. Participation is voluntary, and all participants are asked to sign informed consent before starting the study. A dissemination plan operationalises how to achieve a social impact by reaching academic and non-academic stakeholders. A data management plan describes the specific data sets and regulates its deposition and curation. All publications will be open access.

**Trial registration number:**

NCT02629666; Pre-results.

**Strengths and limitations of this study**

- Process evaluation is an essential part of the testing of the SITLESS complex intervention.
- The Medical Research Council guidance on ‘Process evaluation of complex interventions’, by Moore *et al.*, is used as a framework for conducting and reporting this process evaluation.
- Mixed methods are used to address the specific aims, and each aim is addressed by several methodological procedures applied with a complementary purpose.
- Important information is identified about the differences and commonalities regarding implementation, context and mechanisms of impact of a complex intervention aimed at increasing physical activity and reducing sedentary behaviour across intervention sites in four European countries.
- Potential limitations are the reduced number of cases interviewed from the control arm and the lack of in-depth analysis of the experiences of participants with lower adherence to the intervention and of those dropping out of the study.

## Introduction

Randomised controlled trials have been traditionally focused on outcome evaluations by linking cause and effect and have been criticised as oversimplistic for ignoring relevant aspects, such as the crucial role of context in shaping outcomes.^1^ Therefore, the guidance on the design and evaluation of trials of complex interventions has emphasised the relevance of the processes to provide greater confidence in the conclusions about the effectiveness and thus improve how they inform future intervention development.^2^ Complex interventions are composed of multiple active components that might, for instance, interact, be directed to change behaviour, allow a degree of tailoring or require a level of skills by those delivering and/or receiving the intervention. In addition, the effects of the intervention might be context-dependent, and the causal pathway between the intervention and the outcome might be long, variable or include more than one causal pathway.^2^

The Medical Research Council (MRC) has recently proposed guidance to structure the process evaluation of clinical trials of complex interventions.^3 4^ This framework is organised around three categories—(1) context, (2) implementation and (3) mechanisms of impact—and considers their mutual relationships. This guideline recommends assessing fidelity and quality of implementation, clarifying causal mechanisms (ie, to get a better understanding of the complex pathways between the intervention and the outcomes, and to identify unexpected mechanisms) and identifying contextual factors associated that might explain why different results could be found across sites. Likewise, the Context and Implementation of Complex Interventions (CICI) framework has been proposed to assess context in detail.^5^ According to the CICI framework, context is defined as ‘anything external to the intervention that may act as a barrier or facilitator to its implementation, or its effect’.^5^ According to Moore *et al.*,^4^ ‘complex interventions work by introducing mechanisms that are sufficiently suited to their context to produce change, while causes of problems targeted by interventions may differ from one context to another’. The CICI framework comprises three dimensions—context, implementation and setting—which interact with one another and with the intervention dimension. Hence, *context* comprises seven domains—geographical, epidemiological, sociocultural, socioeconomic, ethical, legal and political—whereas *setting* refers to the specific physical location in which the intervention is put into practice.

In our current ageing societies, promoting physical activity has become one of the key healthcare policies for the prevention of age-related disability, risk reduction of non-communicable diseases and improvement of quality of life.^6–8^ Moreover, sedentary behaviour has been established as a risk factor for mortality, which is independent of low physical activity, and reducing it is increasingly becoming a relevant public health concern, especially for older adults.^9–12^ Therefore, the SITLESS project was designed to determine whether the effects of exercise referral schemes (ERS) may be enhanced by self-management strategies (SMS), to reduce sedentary behaviour and increase physical activity with the final goal of long-term behaviour change, an improvement in health, physical function, quality of life as well as psychosocial outcomes in community-dwelling older European citizens. In order to achieve these goals, SITLESS was designed as a three-armed pragmatic randomised controlled trial to assess the effectiveness and cost-effectiveness of existing physical activity interventions (ERS) enhanced with SMS, compared with ERS alone and also with general recommendations about physical activity (control group).^13 14^ The ERS intervention aims to improve strength, aerobic capacity and balance during 16 weeks, with two 60 min sessions per week. The SMS consists of one one-to-one session, six group-based sessions and four telephone prompts to encourage behaviour change. Participants are followed up for 12 and 18 months postintervention. The control group receives two sessions consisting of general information and recommendations on healthy ageing. A total of 1338 participants are expected to be included (446 in each arm).

**Figure 1:**
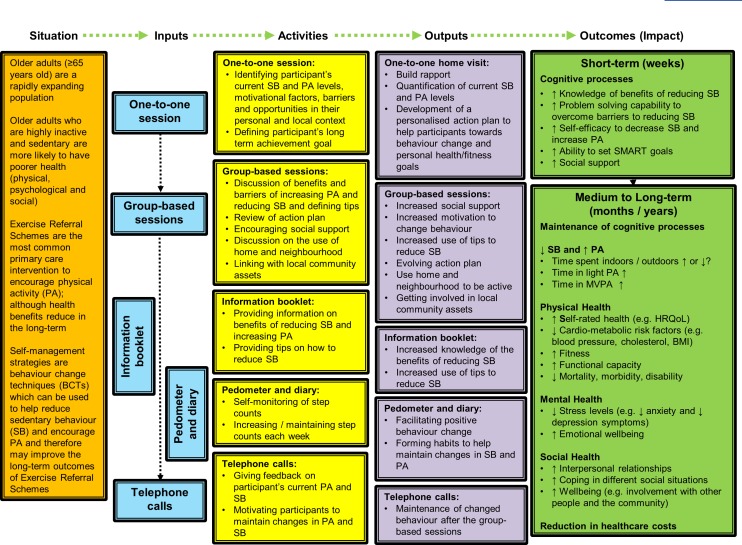
The logic model of the SITLESS intervention. BMI, Body Mass Index; HRQoL, Health-related Quality of Life; MVPA, Moderate and Vigorous Physical Activity; SMART, Specific Measurable Achievable Relevant Time-Oriented.

The SITLESS intervention fulfils the definition of a complex intervention, mainly since it comprises different active components that allow for tailoring to the personal and local characteristics, it is aimed at changing behaviours, and has a wide potential range of short-term and long-term physical, social and psychological effects.^15^ Accordingly, it was built on a logic model presented in figure 1, and the SITLESS trial requires a process evaluation as recommended.

In addition, for the SITLESS study, conducted in the European research framework Horizon 2020, it is a key issue to use the approach of Responsible Research and Innovation (RRI). RRI is understood as an approach that anticipates and assesses potential implications and societal expectations with regard to research and innovation, with the aim to foster the design of inclusive and sustainable research and innovation.^16^ RRI has been defined as an attempt to rethink research as a public good and thus conducted with and for society.^16^ In practice, the SITLESS project has considered specifically public involvement, gender, ethics, open access and governance along the project.

Therefore, the SITLESS project has designed a process evaluation with the aim of understanding how implementation, causal mechanisms and the contextual factors shape the short-term and long-term outcomes. The primary outcomes of the trial include (1) sedentary behaviour as sitting time and time spent in activities which have been traditionally reported as requiring ≤1.5 Metabolic Equivalent of Task (MET), and (2) physical activity as daily counts-per-minute and intensity of exercise performed, both objectively measured by ActiGraph accelerometer. Furthermore, the secondary outcomes include other health-related aspects, quality of life, function and psychosocial outcomes at postintervention and at 12 and 18 months follow-up. The specific aims of the process evaluation are first to assess the implementation of the intervention in terms of (1) fidelity to the intervention design, variability of what was delivered and how between intervention sites; and (2) reach (adherence) of participants to the intervention. Second is to understand the role of context through the following: (1) exploring whether contextual factors regarding the characteristics of the setting, facilitator, the neighbourhood, healthcare system and other location-specific aspects affect implementation and outcomes; (2) assessing the variability and comparability between and within intervention sites; and (3) assessing the generalisability of the intervention’s effectiveness results. Third, the SITLESS project aims to understand the mechanisms of impact. Fourth and finally it aims to explore the perceived effects of the intervention.

**Figure 2:**
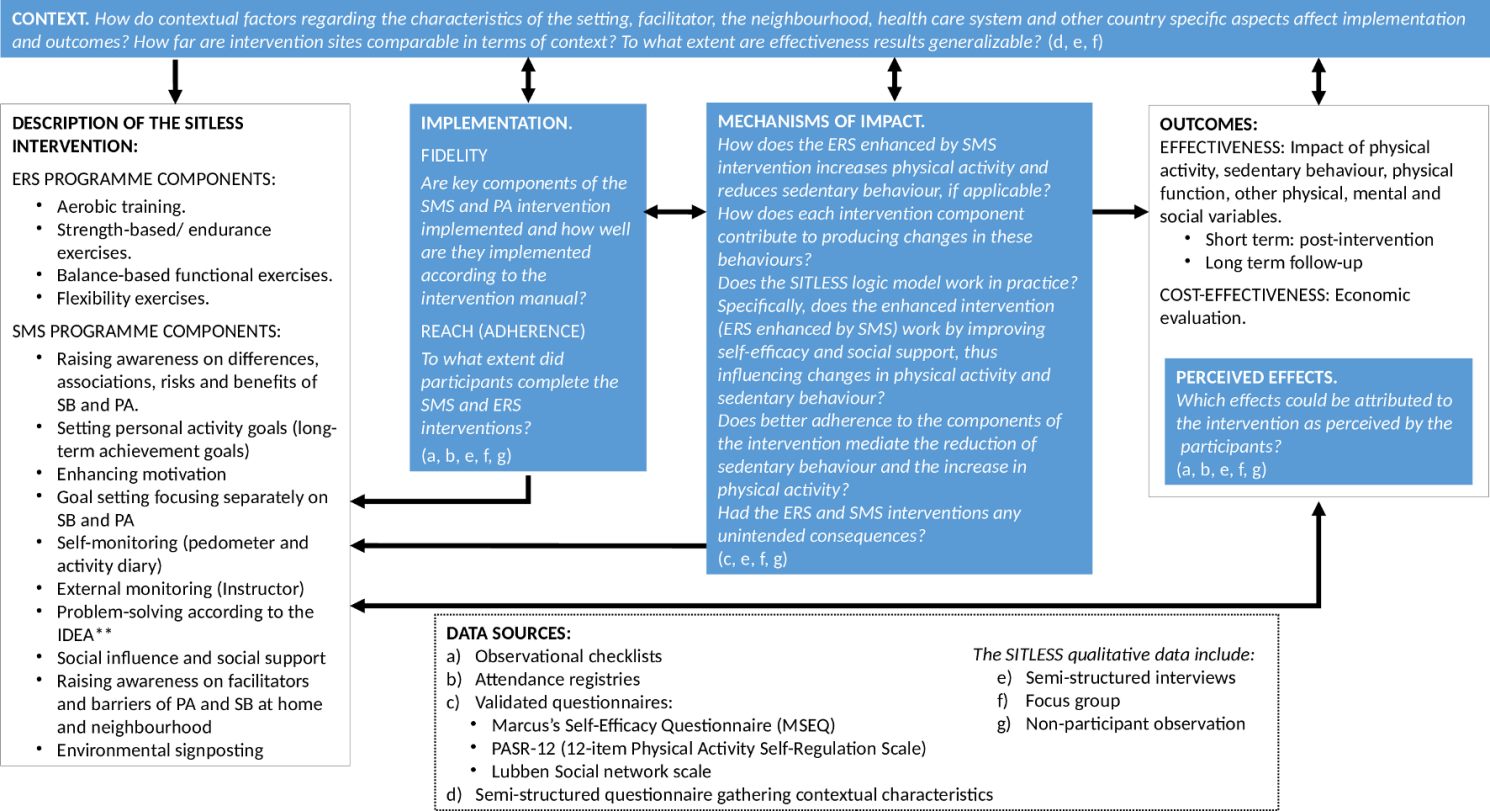
The process evaluation framework for the SITLESS trial. **IDEA refers to the IDEA (Identify Develop Evaluate Analyse) problem solving framework.^24^ ERS, exercise referral schemes; PA, physical activity; SB, sedentary behaviour; SMS, self-management strategies.

The specific research questions corresponding to each aim are shown in figure 2.

## Methods and analysis

### Theoretical frameworks informing this process evaluation

The process evaluation of the SITLESS project follows the guidance on ‘Process evaluation of complex interventions’ from the MRC.^4^ According to this guidance, process evaluation is understood as an essential part of the design and testing of the SITLESS intervention. In addition, context will be specifically evaluated according to the CICI framework.^5^

### The SITLESS process evaluation framework

Figure 2 illustrates the application of the MRC guidance to the SITLESS project. This figure highlights the research question in the categories of implementation, context, mechanisms of impact and perceived effects, and relates them with each methodological procedure applied. In addition, it shows the intervention characteristics and the outcomes.

### Mixed methods used to address the specific aims

A combination of quantitative and qualitative procedures will be applied. This process evaluation was planned prior to implementation of the intervention, except for the semistructured questionnaire on contextual data that was added more recently. Nevertheless, observational checklists, attendance registries and guidance for the qualitative procedures were tested with the first participants and improved accordingly to ease their use and capture the most relevant data.

*Quantitative procedures* quantify the fidelity to the intervention manual and participants’ adherence to the intervention through observational checklists and attendance registries, respectively. They also involve standardised scales to assess the mediating factors (ie, Marcus’s Self-Efficacy Questionnaire (MSEQ),^17^ the 12-item Physical Activity Self-Regulation Scale (PASR-12)^18^ and the Lubben Social Network Scale (LSNS)).^19^ Hence, contextual characteristics will be collected in a table format with prespecified topics (eg, geographical aspects).

*Qualitative procedures* address the same research questions but according to participants’ perceptions, trainers’ experiences and researchers’ observations. Qualitative procedures include participant observation, semistructured interviews and focus groups and are detailed in table 1.

**Table 1:** Specific qualitative procedures, preselected criteria for the purposeful sampling

Qualitative procedure	Target	Preselected criteria for the purposeful sampling and numbers of each profile in each intervention site	Timeframe
Participant observation	Participants in the SMS group	Criteria: 1 from low socioeconomic neighbourhood (or medium, if no low). 1 medium neighbourhood (or high, if no low).	During the SMS intervention.
Semistructured individual interviews	Participants in the SMS group	Criteria (priority low and medium socioeconomic status): 1 frail man and 1 frail woman. 1 robust man and 1 robust woman. 1 for each ethnic minority (if applicable).	End of intervention.
		Criteria: 1 man and 1 woman (priority is low-to-medium socioeconomic status).	12-month follow-up. 18-month follow-up.
	Participants of the ERS group	Criteria: 1 man and 1 woman (priority is low-to-medium socioeconomic status).	12-month follow-up. 18-month follow-up.
	Participants of the control group	Criteria (priority is low-to-medium socioeconomic status): 1 man. 1 woman.	End of intervention. 12-month follow-up. 18-month follow-up.
Focus groups	Participants of the SMS intervention groups	Criteria: 1 low socioeconomic neighbourhood (or medium, if no low). 1 medium neighbourhood (or high, if no low).	End of intervention.
	Participants of the ERS intervention groups	1 (priority is low-to-medium socioeconomic status).	End of intervention.
	ERS and SMS trainers	1 with all trainers involved in the ERS and SMS training.	End of intervention.

ERS, exercise referral schemes; SMS, self-management strategies.

Qualitative methods require specific considerations regarding the research team, reflexivity and the study design.

A multicountry and multidisciplinary research team including a variety of health professionals (medical doctors, epidemiologists, exercise physiologists, physiotherapists and so on) will lead the analysis of the qualitative data gathered in Barcelona, Odense, Ulm and Belfast. All members of the team involved in the qualitative research will be encouraged to keep a research diary to record their reflexive notes, impressions of the data and thoughts about the analysis throughout the process. Researchers should remain flexible and adaptive throughout the process and not be attached to certainty to generate rich findings that could explain the complexity of social issues. Researchers with less experience in qualitative research will be trained to code, index and chart data and to think reflexively about how their identities and experience affect the analysis process in order to help to interpret with accuracy the meaning and significance of the data.

We will apply purposeful sampling of participants with a maximum variation sampling method as a strategy to select a small number of cases that maximise the diversity relevant to the research question. By doing so, we aim to explore the differences and commonalities regarding gender, functional level, ethnicity and socioeconomic background, next to the type of intervention arm (SMS+ERS group, ERS group and control group). Each qualitative procedure targets a specific purposeful sample of participants from each of the four intervention sites (Barcelona, Belfast, Odense and Ulm) according to the characteristics specified in table 1. Moreover, several procedures will be applied at different time-points (postintervention and follow-up) to match with the relevant assessment time-points of the trial. The aims regarding context, mechanisms of impact and perceived effects will be explored at the end of intervention and both follow-up periods, whereas implementation is only assessed at the end of the intervention.

Online Supplementary appendices 1–6 show examples of the templates used as observational checklists and attendance registries, and guidance for the qualitative procedures (semistructured interviews and focus groups).

**Figure 3:**
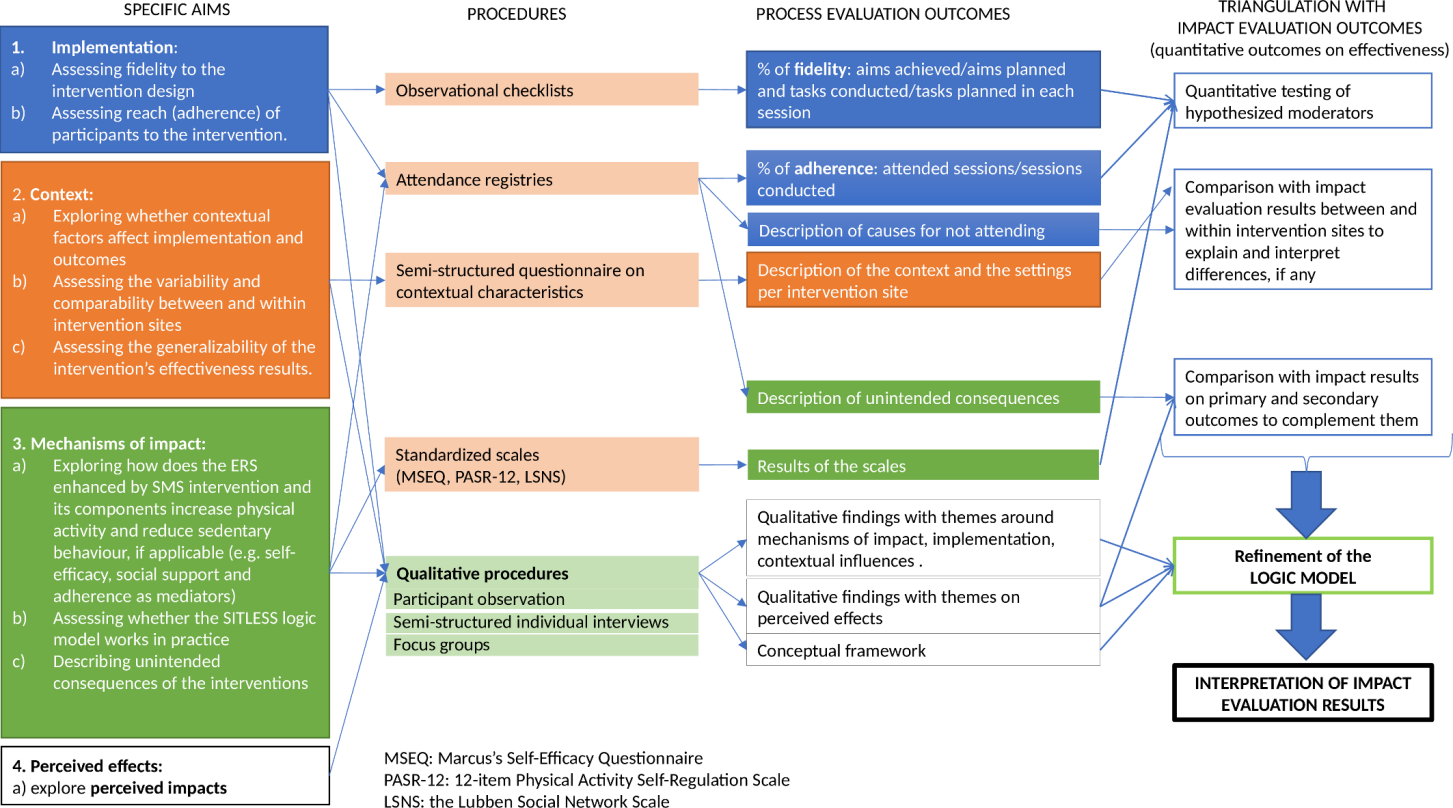
Specific aims, procedures and outcomes of the process evaluation and their triangulation with impact evaluation outcomes. ERS, exercise referral schemes; SMS, self-management strategies.

Each aim is addressed by several methodological procedures applied with a complementary purpose as illustrated in figure 3 and described in detail below.

### Methods to evaluate aim 1

#### Observational checklists

After each session of all SMS and ERS groups, the trainer records the degree of fulfilment of each task and each aim. Hence, the observational checklist of the ERS sessions also covers the implementation characteristics that might vary across intervention sites or across sessions (eg, whether the sessions are conducted indoors or outdoors, and whether and how the trainers tailored the ERS sessions to the frail participants).

#### Attendance registries

All SMS, ERS and control group sessions have a corresponding attendance registry recorded by the trainer after each session. It contains each participant’s attendance, reasons for not attending and adverse events due to the intervention. The attendance is used to calculate the adherence as an equivalent concept of dose and considers the compliance with the intervention (eg, did only part of the exercises due to pain).

#### Qualitative procedures

Semistructured interviews and focus groups with participants will include questions about their perception of the implementation of the specific intervention elements. Focus groups with trainers will have specific questions on their perception of the implementation challenges. In addition, the participant observation of the SMS and ERS sessions by researchers will focus on the implementation of the intervention.

### Methods to evaluate aim 2

#### Prespecified contextual characteristics

The evaluation of this aim is addressed with a table with prespecified domains of context. Specifically, all intervention sites will provide information on the specific intervention setting and context. Setting characteristics will comprise neighbourhood socioeconomic level (low/medium/high), urban/semirural/rural area, type of centres involved with the recruitment, type of centres in which the ERS intervention, SMS intervention and the control group intervention are conducted and existing ERS in these settings. This information will be collected by researchers at each intervention site. Data on context will include those geographical, epidemiological, sociocultural, socioeconomic, ethical, legal and political information that might be relevant as a barrier or facilitator to the intervention implementation, or its effect, for instance existing national programmes on physical activity. These contextual data will be first collected by the leaders of each intervention site and complemented with the knowledge and views of the members of each local advisory board.

#### Qualitative procedures

Semistructured interviews and focus groups with participants and trainers include specific questions about the context. We will specifically explore the role of the physical environment and social context, with the latter comprising the personal circumstances, the personal network and the group dynamic of the SMS+ERS and ERS intervention arms.

### Methods to evaluate aim 3

#### Standardised scales

The MSEQ,^17^ PASR-12^18^ and LSNS^19^ are assessed within the baseline, postintervention and follow-up evaluations to explore the role of self-efficacy, self-monitoring, goal setting, eliciting social support, reinforcements, time management, relapse prevention and social network as mediators of the decrease in sedentary behaviour and increase on physical activity as predicted by the SITLESS logic model (figure 1). The MSEQ has been shown to be a reliable, valid and accurate measure of self-efficacy for physical activity participation in populations with chronic diseases.^20 21^ The PASR-12 has been shown to be a reliable and valid scale for the measurement of self-regulation for physical activity among older adults.^18^ The LSNS is commonly used as a measure of social networks. It has high levels of internal consistency, stable factor structures and high correlations with criterion variables, and clinical cut-points show good convergent validity among European community-dwelling older adults.^22^

#### Qualitative procedures

This will be used to get a better understanding of complex causal pathways and unexpected mechanisms between intervention and outcomes. Specifically, semistructured interviews with participants and focus groups with participants and trainers at the end of the intervention and at follow-up will include questions about perceived effects according to participants’ experiences and trainers’ observations. In addition, the participant observation of the SMS and ERS sessions by researchers will focus on how participants actively respond and interact with the intervention.

### Methods to evaluate aim 4

#### Qualitative procedures

This aim is addressed through semistructured interviews with participants and focus groups with participants and trainers at the end of the intervention and at follow-up. Perceived impacts will be considered as complementary to the results of the effectiveness evaluation assessed with standardised scales.^13^

Within the RRI framework, the SITLESS process evaluation explores if there are differing responses to the intervention by disadvantage categories related with gender, disability, ethnicity and socioeconomic position. It also considers how these categories affect implementation and mechanisms of impact and, consequently, the effectiveness of the intervention.^16^

### Analysis plan

Data from the process evaluation will be analysed according to two main procedures: quantitative and qualitative analyses.

#### Quantitative analysis

Quantitative process evaluation data, recorded in the attendance registries and the observational checklists, will be used to conduct a descriptive analysis, overall and by intervention site. The *SITLESS observational checklists* will be used to calculate the percentage of fidelity of the implementation regarding the tasks and aims of the SMS and ERS sessions in each intervention site. The adherence to the ERS, SMS and control group sessions will be calculated as a percentage of sessions attended with each respective attendance registry. In addition, this registry will allow estimating the frequency of reasons for not attending. Percentage of fidelity of the implementation and adherence will be compared across intervention sites to identify whether differences may exist between them. Comparison will be conducted applying analysis of variance models.

Specific data will be entered in the main study database. This is the case for the variable fidelity and for adherence that will allow us to assess whether a better implemented intervention or a higher dose of the intervention is related with more effect, respectively. The potential mediator role of adherence/dose, the MSEQ, the PASR-12 and the LSNS on the effect of the intervention on physical activity and sedentary behaviour will be explored by covariance analyses, adding these scales as covariates to the mixed linear models testing the effect of the intervention. The statistical efficacy analysis of SITLESS has been described previously.^13^ The information collected in the prespecified table on context characteristics will be used to describe each intervention site.

#### Qualitative analysis

The SITLESS qualitative data analysis will be based on the transcripts of the semistructured interviews and focus groups, and the trainers’ notes from the participant observation. The framework method will be applied to analyse these data.^23^ The framework method belongs to the thematic analysis or qualitative content analysis. These approaches first identify commonalities and differences in qualitative data, and then focus on relationships between different parts of the data to draw descriptive and/or explanatory conclusions clustered around themes. Accordingly, we will apply a combined approach to the analysis, enabling themes to be developed both inductively from the accounts (experiences and views) of research participants and deductively from existing literature. Regular team meetings will facilitate our critical exploration of participant responses, discussion of deviant cases and agreement on recurring themes.

The analysis will be conducted following these steps: transcription, familiarisation with the interview, coding of the transcripts and diaries of the participant observation, developing a working analytical framework, applying the analytical framework, charting data into the framework matrix, and interpreting the data.

The initial coding of the first four transcripts, one per intervention site, will be conducted by two independent researchers, adding coding labels, notes and ideas. As a next step, a working analytical framework will be developed by discussing codes assigned, similarities and differences to achieve an agreement on a set of codes to establish an initial analytical framework. This framework will be refined by coding of further manuscripts until no new codes are generated by two independent researchers. Next, the final analytical framework will be applied to the remaining transcripts. The use of a software to conduct the analysis will be discussed. Once all the data are coded, we will summarise the data in a matrix for each theme.^23^ The matrix will comprise one row per participant and one column per code with a separate sheet for each category, using also illustrative quotations into the corresponding cells. To interpret the data, themes are generated from the data set by reviewing the matrix and making connections within and between participants and categories. Lastly, analytical memos and team discussions will be used to develop themes aimed at offering possible explanations for what is happening within the data agreed by consensus.

### Triangulation with impact evaluation outcomes

As shown in figure 3, outcomes of the process evaluation are triangulated with impact evaluation outcomes (ie, quantitative outcomes on effectiveness) to support their interpretation, thus reinforcing each other. Specifically, quantitative outcomes on fidelity, adherence and from standardised scales are analysed as moderators with the impact evaluation outcomes. Descriptive results on context and qualitative findings on perceived effects are compared with impact results to complement them. Qualitative results and the overall quantitative results will help to refine the logic model to finally support the interpretation of impact evaluation results.

### Patient and public involvement

SITLESS, as an RRI project, has considered in the study design and involved from its onset different types of stakeholders as primary, secondary and tertiary end users.^13^ Stakeholders comprise female and male older adults, representatives of older adults’ associations, primary healthcare and sports professionals, policymakers and, where relevant, other local stakeholders such as health insurance. Specifically, a total of four local advisory boards were created at the beginning of the project, one in each intervention site (Barcelona, Odense, Belfast and Ulm). They met periodically with the local researchers’ team and participated in a number of decisions of the project to consider patients’ priorities and motivations, experience and preferences. Initially, a literature review was conducted, which included how older adults perceive physical activity and sedentary behaviour in order to design an intervention with the potential to achieve sustained changes of healthy behaviour. At each site, we initially explored older adults’ experiences, preferences and priorities regarding behaviour change through focus groups. Their views were used to adapt the intervention design. Local advisory boards contributed to the challenges faced regarding recruitment, retention of participants in the study and the dissemination actions. Once the intervention design was finished, a feasibility study was conducted in the four sites and evaluated with qualitative interviews to the participants. Their perceptions were gathered and used to improve the intervention for the definitive trial.

Qualitative procedures undertaken in the trial include questions to assess whether participants perceive any burden due to the intervention and to their participation in the study.

The dissemination plan comprises sharing the results of the trial at each healthcare, social or leisure centre involved in the recruitment and/or intervention to reach end users, health professionals and other relevant stakeholders. Further dissemination activities will be held to involve participants of each site as much as possible.

## Ethics and dissemination

### Dissemination

The SITLESS dissemination plan operationalises the main dissemination goal, which is to achieve a social impact by reaching academic and non-academic stakeholders, that is, primary care and healthcare professionals, older adults, policymakers, professionals related to physical activity promotion and so on. Specifically, process evaluation results will enhance social impact by providing implementation details that might be of interest for policymakers. In addition, qualitative results (eg, quotations of participants) will support reaching non-academic stakeholders. A data management plan has been developed for the SITLESS study describing each specific data set in the study and regulating its deposition and curation.

All publications from the SITLESS project, including those related with process evaluation, will be open access.

## Discussion

The SITLESS project has developed a process evaluation to understand how implementation, causal mechanisms and the contextual factors shape the outcomes of a complex intervention that enhances ERS with SMS to reduce sedentary behaviour and increase physical activity among community-dwelling older adults of four European countries. Accordingly, this trial has the purpose of facilitating future intervention development by informing policymakers, practitioners and researchers about implementation, mechanisms of impact and contextual factors in detail. In case the intervention is proven as being effective and cost-effective, the information will be focused on the delivered intervention to allow replication in new contexts considering the relevant core components, context characteristics and implementation challenges. In case of being ineffective, process evaluation results will contribute to define whether the failure can be attributed to the intervention itself or to a poor implementation. Hence, process evaluation will provide useful data for systematic reviews to compare interventions considering their implementation and contextual characteristics.

### Trial status

SITLESS started in May 2015. The feasibility study was conducted from December 2015 until June 2016. The recruitment for the trial started in July 2016. At the time of submission of this protocol, all 1390 participants have been included in the study. To date, follow-up assessments are being conducted and are planned to finish by September 2019.

10.1136/bmjopen-2018-027073.supp1 Supplementary Appendix 1

10.1136/bmjopen-2018-027073.supp2 Supplementary Appendix 2

10.1136/bmjopen-2018-027073.supp3 Supplementary Appendix 3

10.1136/bmjopen-2018-027073.supp4 Supplementary Appendix 4

10.1136/bmjopen-2018-027073.supp5 Supplementary Appendix 5

10.1136/bmjopen-2018-027073.supp6 Supplementary Appendix 6

## Supplementary Material

Reviewer comments

Author's manuscript
