# Supplementary material for: Exercise referral schemes enhanced by self-management strategies to reduce sedentary behaviour and increase physical activity among community-dwelling older adults from four European countries: protocol for the process evaluation of the SITLESS randomised controlled trial
Source: BMJ Open. 2019 Jun 14;9(6):e027073. doi: 10.1136/bmjopen-2018-027073 (PMC6588992; doi:10.1136/bmjopen-2018-027073)
Supplement: Supplementary Appendix 1 [file bmjopen-2018-027073supp001.pdf]

Appendix 1: SEMI-STRUCTURED QUESTIONNAIRE FOR INTERVIEWS WITH PARTICIPANTS OF THE SMS GROUP

Note: Aspects related with gender, frailty/robustness and ethnicity have been considered as elegibility criteria and will be considered in the content analysis. However, this questionnaire does not include any specific question regarding these aspects and are considered to emerge in their discourse.

Part one: introduction (5')

Section	Comments
**Introduction**	• Acknowledgements<br>• The conductor introduces briefly him/her self to the participant<br>• Inform about the duration and the aims of the meeting<br>• The conductor informs the participant that the meeting will be audio-recorded for later analysis.<br>• The conductor reminds the participant to turn-off or silence the phone.
**Informed consent**	• The conductor asks for permission about recording of the interview and informed consent.<br>• Reading and signing of informed consent.

Part two: overall mechanisms of impact (20')

Dimension	Questions
*According to participant' perceptions: how do the participant' actively respond and interact with the **overall intervention**?*<br><br>__Note:__ take into account that the SMS intervention requires following tasks:<br>◦ *Fill the diary daily: steps or PA time, and calculate means.*<br>◦ *Wear daily, open and read the pedometer.*<br>◦ *Set goals on PA and follow-up accomplishment.*<br>◦ *Set goals on SB and follow-up accomplishment (SITLESS tips).*<br>◦ *Active participation in group discussions.*<br>◦ *Telephone prompts*	• What are your general impression / thoughts of the intervention?<br>• Overall how satisfied are you with the intervention?<br>• What specific features of the intervention impressed you?<br>• What features of the intervention disappointed you?<br>• How likely would you be to recommend this intervention to a family member or friend? Why?<br>• What, if anything, do you find frustrating or unappealing about the intervention?<br>• Do you have any suggestions on how we could improve the intervention to make it more appealing?<br>• Were any of tasks required in the intervention difficult for you to perform?

Part three: overall perceived effects and mechanisms of impact (20')

Dimension	Questions
**Secondary outcomes**	• Have you perceived any effects of the intervention? Which ones?<br>• Have you perceived any specific effects on<br>o General health?<br>o Mental health? In terms of depression, loneliness, etc. and psychological/emotional wellbeing?<br>o Physical health?<br>o Social relationships?
**Primary outcomes: Effects on PA and SB**	
How does the SMS intervention produce change?	• During the intervention: have you increased your PA? (besides the ERS sessions)<br>• In case that you have increased your PA, why do you think you have done it?, Because your physical capacity increased<br>o Because your self-efficacy increased<br>o For other reasons<br>• In case that you have not increased your PA, why do you think you haven't done it?<br>• During the program: have you decreased your SB?<br>• If so, in which manner?<br>o Incorporate new routines<br>o Other
**Mid-long term effects on PA and SB**	• If not, why do you think that?<br>• Do you think you will maintain your increased PA / decreased SB once the intervention has finished?<br>• If so, in which manner?<br>• If not, why do you think that?

Part four: mechanisms of impact for each component (20')

<table>
<tr><th>Dimension</th><th>Questions</th></tr>
<tr><td>

How do each intervention components contribute to produce change?
Specifically, does the intervention work by improving self-efficacy and social support?
Does a better accomplishment of the goal setting, self-monitoring and cues mediate the reduction of sedentary behaviour and the increase in physical activity?"

</td><td>

Can you state the strategies you have used to accomplish each session's goals? Have you accomplished them by yourself?
What are the reasons why you have decided to accomplish (or not) the goals?
How and why each component supported/permitted to achieve the goals of the intervention? (mechanisms of impact)

Raising awareness on differences, associations, risks and benefits of SB and PA.
Setting personal activity goals (long-term achievement goals)
Goal setting focusing separately on PA
Goal setting focusing separately on SB (SITLESS tips)
Self-monitoring:

pedometer
activity diary

External monitoring (Instructor)
Problem-solving
Raising awareness on facilitators and barriers of PA and SB at home and at the neighborhood (environmental signposting)
Peer and social support from the group
The trainer
Telephone prompts

</td></tr>
</table>

Part five: context (20')

Dimension	Questions
**Physical environment**<br><br>*How do contextual factors regarding the characteristics of the neighbourhood affect outcomes according to participants' perceptions?*	• How do you perceive the physical environment of your neighbourhood?<br>• How do you perceive the specific settings of the intervention? (i.e., facilities used for the SMS and PA sessions)<br>○ Primary health care centre<br>○ Parks<br>○ Sports facilities<br>○ Community resources<br>• How do you perceive these specific aspects of your neighbourhood? (especially considering whether and how these aspects affect doing PA in the neighbourhood)<br>○ Socio-economic level<br>○ Urban/rural setting<br>○ Orography / walkability<br>○ Pleasant environment (comfortable, traffic…)<br>○ Personal safety (crimes, robberies, …)<br>○ Availability of places within an easy walking distance to do informal PA (parks, urban gym, …) and to do formal PA (organized PA offers)
**Personal networks**<br><br>*How do contextual factors regarding the characteristics of the personal network affect outcomes according to the participant's perceptions?*	• Do you think your personal situation or health status (e.g., having a supportive social network, being a caregiver, living alone, having a chronic disease, etc.) helps or hinders your physical activity and your sedentary behavior? How and why?<br>• Do you think your personal network (partners, family, friends) helps or hinders your physical activity and your sedentary behavior? How and why?<br>• Did you know other participants of the group before? How has that affected your participation in the group?
