# Supplementary material for: Exercise referral schemes enhanced by self-management strategies to reduce sedentary behaviour and increase physical activity among community-dwelling older adults from four European countries: protocol for the process evaluation of the SITLESS randomised controlled trial
Source: BMJ Open. 2019 Jun 14;9(6):e027073. doi: 10.1136/bmjopen-2018-027073 (PMC6588992; doi:10.1136/bmjopen-2018-027073)
Supplement: Supplementary Appendix 2 [file bmjopen-2018-027073supp002.pdf]

Appendix 2: SEMI-STRUCTURED QUESTIONNAIRE FOR THE FOCUS GROUP FOR THE SMS INTERVENTION GROUPS

Part one: introduction and presentation of participants (10')

Section	Comments
**Introduction**	• Acknowledgements<br>• The conductor introduces briefly him/her self to the participants<br>• Inform about the duration and the aims of the meeting<br>• The conductor informs the participants that the meeting will be audio-recorded for later analysis.<br>• The conductor reminds the participants to turn-off or silence phones.
**Informed consent**	• The conductor asks for permission about recording of the meeting and informed consent.<br>• Reading and signing of informed consent.
**Presentation of participants**	• Kindly ask participants to introduce their selves to the rest of the group.

Part two: overall mechanisms of impact (20')

Dimension	Questions
*According to participants' perceptions: how do participants' actively respond and interact with the **overall intervention**?*<br><br>**<u>Note:</u>** take into account that the SMS intervention requires following tasks:<br>○ *Fill the diary daily: steps or PA time, and calculate means.*<br>○ *Wear daily, open and read the pedometer.*<br>○ *Set goals on PA and follow-up accomplishment.*<br>○ *Set goals on SB and follow-up accomplishment (SITLESS tips).*<br>○ *Active participation in group discussions.*<br>○ *Telephone promots*	• What are your general impression / thoughts of the intervention?<br>• Overall how satisfied are you with the intervention?<br>• What specific features of the intervention impressed you?<br>• What features of the intervention disappointed you?<br>• How likely would you be to recommend this intervention to a family member or friend? Why?<br>• What, if anything, do you find frustrating or unappealing about the intervention?<br>• Do you have any suggestions on how we could improve the intervention to make it more appealing?<br>• Were any of tasks required in the intervention difficult for you to perform?

Part three: overall perceived effects and mechanisms of impact (20')

Dimension	Questions
**Secondary outcomes**	• Have you perceived any effects of the intervention? Which ones?<br>• Have you perceived any specific effects on<br>○ General health?<br>○<br>○ Mental health? In terms of depression, loneliness, etc. and psychological/emotional wellbeing?<br>○ Physical health?<br>○ Social relationships?
**Primary outcomes: Effects on PA and SB**<br><br>*How does the SMS intervention produce change?*	• During the intervention: have you increased your PA? (besides the ERS sessions)<br>• In case that you have increased your PA, why do you think you have done it?, Because your physical capacity increased<br>○ Because your self-efficacy increased<br>○ For other reasons<br>• In case that you have not increased your PA, why do you think you haven't done it?<br>• During the program: have you decreased your SB?<br>• If so, in which manner?<br>○ Incorporate new routines<br>○ Other<br>• If not, why do you think that?
**Mid-long term effects on PA and SB**	• Do you think you will maintain your increased PA / decreased SB once the intervention has finished?<br>• If so, in which manner?<br>• If not, why do you think that?

Part four: mechanisms of impact for each component (20’)

Dimension	Questions
▸ *How do **each intervention components** contribute to produce change?*<br>▸ *Specifically, does the intervention work by improving self-efficacy and social support?*<br>▸ *Does a better accomplishment of the goal setting, self-monitoring and cues mediate the reduction of sedentary behaviour and the increase in physical activity?”*	• Can you state the strategies you have used to accomplish each session’s goals? Have you accomplished them by yourself?<br>• What are the reasons why you have decided to accomplish (or not) the goals?<br>• How and why each component supported/permitted to achieve the goals of the intervention? (mechanisms of impact)<br>○ Raising awareness on differences, associations, risks and benefits of SB and PA.<br>○ Setting personal activity goals (long-term achievement goals)<br>○ Goal setting focusing separately on PA<br>○ Goal setting focusing separately on SB (SITLESS tips)<br>○ Self-monitoring:<br>▪ pedometer<br>▪ activity diary<br>○ External monitoring (Instructor)<br>○ Problem-solving<br>○ Raising awareness on facilitators and barriers of PA and SB at home and at the neighborhood (environmental signposting)<br>○ Peer and social support from the group<br>○ The trainer<br>○ Telephone prompts

Part five: context (20')

Dimension	Questions
**Physical environment**<br><br>*How do contextual factors regarding the characteristics of the neighbourhood affect outcomes according to participants' perceptions?*	• How do you perceive the physical environment of your neighbourhood?<br>• How do you perceive the specific settings of the intervention? (i.e., facilities used for the SMS and PA sessions)<br>○ Primary health care centre<br>○ Parks<br>○ Sports facilities<br>○ Community resources<br>• How do you perceive these specific aspects of your neighbourhood? (especially considering whether and how these aspects affect doing PA in the neighbourhood)<br>○ Socio-economic level<br>○ Urban/rural setting<br>○ Orography / walkability<br>○ Pleasant environment (comfortable, traffic…)<br>○ Personal safety (crimes, robberies, …)<br>○ Availability of places within an easy walking distance to do informal PA (parks, urban gym, …) and to do formal PA (organized PA offers)
**Personal networks**<br><br>*How do contextual factors regarding the characteristics of the personal network affect outcomes according to participants' perceptions?*	• Do you think your personal situation or health status (e.g., having a supportive social network, being a caregiver, living alone, having a chronic disease, etc.) helps or hinders your physical activity and your sedentary behavior? How and why?<br>• Do you think your personal network (partners, family, friends) helps or hinders your physical activity and your sedentary behavior? How and why?<br>• Did you know other participants of the group before? How has that affected your participation in the group?
