# Supplementary material for: Exercise referral schemes enhanced by self-management strategies to reduce sedentary behaviour and increase physical activity among community-dwelling older adults from four European countries: protocol for the process evaluation of the SITLESS randomised controlled trial
Source: BMJ Open. 2019 Jun 14;9(6):e027073. doi: 10.1136/bmjopen-2018-027073 (PMC6588992; doi:10.1136/bmjopen-2018-027073)
Supplement: Supplementary Appendix 3 [file bmjopen-2018-027073supp003.pdf]

Appendix 3: OBSERVATIONAL CHECKLIST OF THE SMS INTERVENTION SESSIONS

Centre: Group: Trainer:

Date: Session number: 2	
Week: 3	Stage: Ramping

FIRST GROUP-BASED SESSION Length of session: ______ minutes

**Tasks**	**Completed**	**Partially completed**	**Not completed**
**Activity 1** - Identifying the benefits of being less sedentary and more active.			
**Activity 2** - Share and discuss personal lifestyles related to sedentary behaviour, physical activity and physical exercise.			
**Activity 3** - Share and discuss tips for sitting less			
**Activity 4** - Retrieve the participants' baseline daily steps and/or weekly time in physical activities / exercise and discuss the reasons for differences between certain days.			
**Activity 5** - Agree on which SITLESS tips the participants will try over the next week.			
**FINAL TASKS**<br>• Make a statement to encourage the group to practise the SITLESS tips:<br>• The final tasks are to arrange a date/time for the next session and to remind the participants to bring their information booklets and activity diaries			

**Aims of the session**	**Completed**	**Partially completed**	**Not completed**
1. To identify the benefits of being less sedentary and more active.			
2. To share and discuss personal lifestyles related to sedentary behaviour, physical activity and physical exercise			
3. To share and discuss tips for sitting less.			
4. To retrieve the participants' baseline daily steps and/or weekly time in physical activities / exercise and discuss the reasons for differences between certain days.			
5. To agree on which SITLESS tips the participants will try over the next week.			

Centre: Group: Trainer:

Date:	Session number:	
	Week:	Stage:

Which tasks went particularly well during the session? Why?

Were any tasks difficult to implement? Why?

Were any modifications made to the planned session? Why? *(adhoc tailoring)*

Have you observed any adverse effects? Which ones?

Generally, what was the participant's response / attitude to the different activities (e.g. uninterested, excitable, attentive, sceptical, quiet, chatty)? Have you observed specific responses to be highlighted regarding gender, educational level, ethnicity..? Which ones?

Other comments
