# Supplementary material for: Exercise referral schemes enhanced by self-management strategies to reduce sedentary behaviour and increase physical activity among community-dwelling older adults from four European countries: protocol for the process evaluation of the SITLESS randomised controlled trial
Source: BMJ Open. 2019 Jun 14;9(6):e027073. doi: 10.1136/bmjopen-2018-027073 (PMC6588992; doi:10.1136/bmjopen-2018-027073)
Supplement: Supplementary Appendix 4 [file bmjopen-2018-027073supp004.pdf]

Appendix 4: OBSERVATIONAL CHECKLIST OF THE PA SESSIONS

Note: This annex contains the standard Observation Checklist of the PA sessions with all the information needed from each intervention site. This checklist has been adapted by each intervention site in order to avoid repetition by keeping track only of aspects of the PA interventions that may vary from session to session. E.g., Odense is conducting all sessions indoors and trainers do not need to register this aspect after each session.

Centre: Group: Trainer:

Date: Session number:

This checklist should be filled in by the PA instructor after EACH physical activity session

DATA ON EACH SESSION REPORTED BY THE INSTRUCTOR

1. Where have you conducted the physical activity session?

Indoors/outdoors Specific setting:___________

2. Has the climate / environment affected this session? *(for an individual / overall group)* YES/NO

How? ___________________________________

3. Was this session conducted by the usual instructor? YES/NO
4. Have you tailored the physical activity session to the frail participants? YES/NO

How?____

5. Have you personalized the progression in the exercise intensity? YES/NO

How?____

6. Have you tailored the physical activity session to specific participants for other reasons? YES/NO

How? ____

7. Use of equipment/ material:

Indicate the use of weight lifting equipment	Yes	No
Indicate the use of cardiovascular machines	Yes	No
Indicate the use of other material (e.g. therabands, balls, bosu)	Yes<br>If yes, which:_______<br>__________________	No
Indicate the use of recycled/ adapted material	Yes	No
Indicate the use of music	Yes	No

8. Have you applied any group dynamics to promote group cohesion? (e.g., cooperative games, work in pairs...)

How? ____

9. Every second session, use the final 5 minutes of session to ask the participants whether they have done the extra Physical Activity session on their own per week (defined as at least 30 minutes of moderate or vigorous PA following the WHO guidelines, for instance brisk walking)

Number of participants that had done the extra Physical Activity session/ out of the total attending → calculate percentage:

………………./………………: ____%

10. Observations relevant for the study (e.g. gender specific issues?)

Please add here observations on the development of the session. Add the participant code to refer to observations of specific participants

……

……

……

……

……

……
