# Supplementary material for: Exercise referral schemes enhanced by self-management strategies to reduce sedentary behaviour and increase physical activity among community-dwelling older adults from four European countries: protocol for the process evaluation of the SITLESS randomised controlled trial
Source: BMJ Open. 2019 Jun 14;9(6):e027073. doi: 10.1136/bmjopen-2018-027073 (PMC6588992; doi:10.1136/bmjopen-2018-027073)
Supplement: Supplementary Appendix 5 [file bmjopen-2018-027073supp005.pdf]

Appendix 5: ATTENDANCE REGISTRY FOR THE SMS SESSIONS

Centre: Group: Trainer:

		Dates of the sessions (dd/mm/yy)										
**Participant code**	**Participant name**											
		One-to-one	1st group session	2nd group session	3rd group session	4th group session	5th group session	6th group session	1st phone call	2nd phone call	3rd phone call	4th phone call
**EXAMPLE:** CL004	Maria Roca	√ 01.02.16	√	¥ B	√	√	√	√	√ 5/05/16	√ 6/07/16	∏ E 01-15/10/16	∏ E 01-15/01/17

√ = Attendance. In case a certain participant arrived considerably late or performed only part of the session (e.g., arrived 20 minutes late or did half of the exercises due to pain), report the % performed of the session.

¥ = Unexcused absence (e.g.., the trainer was NOT previously informed)

∏ = Excused absence (e.g.., the trainer was previously informed)

Appendix 5: ATTENDANCE REGISTRY FOR THE SMS SESSIONS

Centre: Group: Trainer:

Please, state the reason for non-attendance: (A) health problem (cold, illness, surgery, etc), (B) medical visit, (C) trip/travel, (D) familiar reason (taking care of grandsons, etc), (E) other (specify).

Please, state any **adverse effect** for participant and session (e. g. anxiety).

Please, fill the table in case you need to specify the reason for non-attendance or the adverse effect. Include those objective or perceived adverse effects that happened during this SMS session, that participants and/or the trainer attribute to the SMS intervention.

**Participant code**	**Date**	**Note of reasons for non-attendance**	**Note on adverse effect**
CL004	01-15/10/16	Participant not reached after repeated calls	
CL004	01-15/01/17	Participant not reached after repeated calls	
