# Supplementary material for: Exercise referral schemes enhanced by self-management strategies to reduce sedentary behaviour and increase physical activity among community-dwelling older adults from four European countries: protocol for the process evaluation of the SITLESS randomised controlled trial
Source: BMJ Open. 2019 Jun 14;9(6):e027073. doi: 10.1136/bmjopen-2018-027073 (PMC6588992; doi:10.1136/bmjopen-2018-027073)
Supplement: Supplementary Appendix 6 [file bmjopen-2018-027073supp006.pdf]

Appendix 6: ATTENDANCE REGISTRY FOR THE PA SESSIONS Centre: Group:

Trainer:

			Dates of the sessions (dd/mm/yy)															
**Participant code**	**Participant name**	**SPPB**	01.02 .16	03.02 .16	08.02 .16	10.0 2.16	15.02. 06	17.02. 16										
**EXAMPLE:** CL004	Maria Roca	L	√	X	∏ B	√	√ 2 50%	∏ E										

√ = Attendance. In case a certain participant arrived considerably late or performed only part of the session (e.g., arrived 20 minutes late or did half of the exercises due to pain), report the % performed of the session.

X = Unexcused absence (i.e., the trainer was NOT previously informed)
∏ = Excused absence (i.e., the trainer was previously informed)
Please, state the **SPPB** (Short Physical Performance Battery) as L (low), M (medium), or H (high)

Please, state any **adverse effect** of physical activity (PA) for participant and session, according to following codes: (1) stiffness after PA, (2) joint pain, (3) fall during PA session, (4) dizziness, (5) other (specify)

Please, state the **reason for non-attendance**: (A) health problem (cold, illness, surgery, etc.), (B) medical visit, (C) trip/travel, (D) familiar reason (taking care of grandsons, etc.), (E) other (specify)

Please, fill the table in case you need to specify the reason for non-attendance or the adverse effect. Include those objective or perceived adverse effects that participants and/or the trainer attribute to the PA session.

**Participant code**	**Date**	**Note of reasons for non-attendance**	**Note on adverse effect**
CL004	17.02.16	Forgot the session	
