# Supplementary material for: Exercise referral schemes enhanced by self-management strategies to reduce sedentary behaviour and increase physical activity among community-dwelling older adults from four European countries: protocol for the process evaluation of the SITLESS randomised controlled trial
Source: BMJ Open. 2019 Jun 14;9(6):e027073. doi: 10.1136/bmjopen-2018-027073 (PMC6588992; doi:10.1136/bmjopen-2018-027073)
Supplement: Author's manuscript [file bmjopen-2018-027073.draft_revisions.pdf]

BMJ Open

BMJ Open is committed to open peer review. As part of this commitment we make the peer review history of every article we publish publicly available.

When an article is published we post the peer reviewers' comments and the authors' responses online. We also post the versions of the paper that were used during peer review. These are the versions that the peer review comments apply to.

The versions of the paper that follow are the versions that were submitted during the peer review process. They are not the versions of record or the final published versions. They should not be cited or distributed as the published version of this manuscript.

BMJ Open is an open access journal and the full, final, typeset and author-corrected version of record of the manuscript is available on our site with no access controls, subscription charges or pay-per-view fees (http://bmjopen.bmj.com).

If you have any questions on BMJ Open's open peer review process please email info.bmjopen@bmj.com

BMJ Open

Exercise referral schemes enhanced by self-management strategies to reduce sedentary behaviour and increase physical activity among community-dwelling older adults from four European countries: protocol for the process evaluation of The SITLESS randomised controlled trial

Journal:	*BMJ Open*
Manuscript ID	bmjopen-2018-027073
Article Type:	Protocol
Date Submitted by the Author:	04-Oct-2018
Complete List of Authors:	Coll-Planas, Laura; Universitat Autònoma de Barcelona, Fundació Salut i Envelliment (Foundation on Health and Ageing)- UAB; Institute of Biomedical Research (IIB Sant Pau)<br>Blancafort Alias, Sergi; Universitat Autònoma de Barcelona, Fundació Salut i Envelliment (Foundation on Health and Ageing)- UAB; Institute of Biomedical Research (IIB Sant Pau)<br>Tully, Mark; Ulster University, Institute of Mental Health Sciences, School of Health Sciences; Queen's University Belfast, 2. UKCRC Centre of Excellence for Public Health Research (Northern Ireland)<br>Caserotti, Paolo; Syddansk Universitet, Department of Sports Science and Clinical Biomechanics, Center for Active and Healthy Ageing (CAHA)<br>Giné-Garriga, Maria; Universitat Ramon Llull, Facultat de Psicologia, Ciències de l'Educació i de l'Esport Blanquerna<br>Blackburn, Nicole; Queen's University Belfast School of Medicine Dentistry and Biomedical Sciences, UKCRC Centre of Excellence for Public Health (NI), Centre for Public Health<br>Skjødt, Mathias; Syddansk Universitet , Department of Sports Science and Clinical Biomechanics, Center for Active and Healthy Ageing (CAHA)<br>Wirth, Katharina ; Universitat Ulm, Institute of Epidemiology and Medical Biometry; Agaplesion Bethesda Clinic, Geriatric Research Unit Ulm University and Geriatric Center Ulm Alb-Donau,<br>Deidda, Manuela; University of Glasgow, Health Economics and Health Technology assessment<br>McIntosh, Emma; University of Glasgow Institute of Health and Wellbeing, Health Economics and Health Technology Assessment (HEHTA)<br>Rothenbacher, Dietrich; Ulm University, Institute of Epidemiology and Medical Biometry<br>Gallardo, Rodrigo; Universidad Catolica de la Santisima Concepcion, Departamento de Ciencias del Deporte y Acondicionamiento Físico, CEADE<br>Jerez, Javier; Universitat Ramon Llull, 1. Facultat de Psicologia, Ciències de l'Educació i de l'Esport Blanquerna; University of Vic-Central University of Catalonia (UVIC-UCC), Faculty of Health Science and Welfare. Centre for Health and Social Care Research (CESS).<br>Sansano-Nadal, Oriol; Universitat Ramon Llull, Facultat de Psicologia, Ciències de l'Educació i de l'Esport Blanquerna

Santiago, Marta; Universitat Ramon Llull, Facultat de Psicologia, Ciències de l’Educació i de l’Esport Blanquerna
Wilson, Jason; Queen's University Belfast School of Medicine Dentistry and Biomedical Sciences, Centre for Public Health
Guerra-Balic, Míriam ; Universitat Ramon Llull, Facultat de Psicologia, Ciències de l’Educació i de l’Esport Blanquerna
Martín-Borràs, Carme; Faculty of Psychology, Education and Sport Sciences (FPCEE) Blanquerna, Ramon Llull University., Department of Physical Activity and Sport Sciences; Faculty of Health Sciences (FCS) Blanquerna, Ramon Llull Univesity., Department of Physical Therapy
Gonzalez, Denise; Sport Initiative et Loisir Bleu Association; Sport Initiative et Loisir Bleu Association
Lefebvre, Guillaume; Sport Initiative et Loisir Bleu Association; Sport Initiative et Loisir Bleu Association
Denkinger, Michael; Universitat Ulm, Agaplesion Bethesda Clinic, Geriatric Research Unit Ulm University and Geriatric Center Ulm Alb-Donau
Kee, Frank; Queen's University Belfast, UKCRC Centre of Excellence for Public Health (NI)
Salvà Casanovas, Antoni ; Universitat autonoma de Barcelona, Fundació Salut i Envelliment (Foundation on Health and Ageing)- UAB; Institute of Biomedical Research (IIB Sant Pau)
Roqué i Figuls , Marta; Universitat Autònoma de Barcelona, Fundació Salut i Envelliment (Foundation on Health and Ageing)- UAB; Institute of Biomedical Research (IIB Sant Pau)

Keywords: EPIDEMIOLOGY, PUBLIC HEALTH, GERIATRIC MEDICINE

SCHOLARONE™
Manuscripts

TITLE PAGE:

The title page must contain the following information:

- Title of the article.

Exercise referral schemes enhanced by self-management strategies to reduce sedentary behaviour and increase physical activity among community-dwelling older adults from four European countries: protocol for the process evaluation of The SITLESS randomised controlled trial

Authors: **Coll-Planas, Laura; Blancafort, Sergi; Tully, Mark A; Caserotti, Paolo; Giné-Garriga, Maria; Blackburn, Nicole E; Skjødt, Mathias; Wirth, Katharina; Deidda, Manuela; McIntosh, Emma; Rothenbacher, Dietrich; Gallardo Rodríguez, Rodrigo; Jerez-Roig, Javier; Sansano-Nadal, Oriol; Santiago, Marta; Wilson, Jason J; Guerra-Balic, Miriam; Martín-Borràs, Carme; González, Denise; Denkinger, Michael; Kee, Frank; Salvà, Antoni; Roqué i Figuls, Marta.**

- Full name, postal address, e-mail and telephone number of the **corresponding author.**

Full name: Laura Coll i Planas
Postal address: Fundació Salut i Envelliment (Foundation on Health and Ageing)- Universitat Autònoma de Barcelona
Sant Antoni Maria Claret, 171 E-08041
Barcelona
E-mail: laura.coll@uab.cat

Tel.: +34 93 446 45 06

- Full name, department, institution, city and country of all co-authors.

Coll-Planas, Laura
Blancafort, Sergi
Salvà, Antoni
Roqué i Figuls, Marta.

1. Fundació Salut i Envelliment (Foundation on Health and Ageing)- Universitat Autònoma de Barcelona
   Barcelona, Spain
2. Institute of Biomedical Research (IIB Sant Pau), Barcelona, Spain

Tully, Mark A

1. Institute of Mental Health Sciences, School of Health Sciences, Ulster University, Newtownabbey, Northern Ireland, UK.
2. UKCRC Centre of Excellence for Public Health Research (Northern Ireland), Queen's University Belfast, Belfast, Northern Ireland, UK.

For peer review only - http://bmjopen.bmj.com/site/about/guidelines.xhtml

Blackburn, Nicole E
Wilson, Jason J;
Kee, Frank
UKCRC Centre of Excellence for Public Health (NI), Centre for Public Health, School of Medicine, Dentistry and Biomedical Sciences, Queen's University Belfast, Belfast, UK.

Caserotti, Paolo
Skjodt, Mathias
Department of Sports Science and Clinical Biomechanics, Center for Active and Healthy Ageing (CAHA), University of Southern Denmark, Odense, Campusvej 55, 5230 Odense M, Denmark

Giné-Garriga, Maria
Sansano-Nadal, Oriol
Santiago, Marta
Guerra, Míriam
Martín-Borràs, Carme
Facultat de Psicologia, Ciències de l'Educació i de l'Esport Blanquerna (Universitat Ramon Llull), C/ Císter 34, 08022 Barcelona, Spain

Jerez-Roig, Javier
1. Facultat de Psicologia, Ciències de l'Educació i de l'Esport Blanquerna (Universitat Ramon Llull), C/ Císter 34, 08022 Barcelona, Spain
2. Research group on Methodology, Methods, Models and Outcomes of Health and Social Sciences (M3O). Faculty of Health Science and Welfare. Centre for Health and Social Care Research (CESS). University of Vic-Central University of Catalonia (UVIC-UCC), C.Sagrada Família, 7, 08500 Vic, Spain

Rothenbacher, Dietrich
Institute of Epidemiology and Medical Biometry, Ulm University, 89081 Ulm, Germany.

Wirth, Katharina
1. Institute of Epidemiology and Medical Biometry, Ulm University, 89081 Ulm, Germany
2. Agaplesion Bethesda Clinic, Geriatric Research Unit Ulm University and Geriatric Center Ulm Alb-Donau, Zollernring 26, 89073 Ulm, Germany

Denkinger, Michael;
Agaplesion Bethesda Clinic, Geriatric Research Unit Ulm University and Geriatric Center Ulm Alb-Donau, Zollernring 26, 89073 Ulm, Germany

Deidda, Manuela
McIntosh, Emma
Health Economics and Health Technology Assessment (HEHTA), Institute of Health and Wellbeing (IHW), University of Glasgow, 1 Lilybank Gardens, Glasgow G12 8RZ, UK.

For peer review only

For peer review only - http://bmjopen.bmj.com/site/about/guidelines.xhtml

Gallardo Rodríguez, Rodrigo
Departamento de Ciencias del Deporte y Acondicionamiento Físico, CEADE, Universidad Católica de la Santísima Concepción, Concepción, Chile.

González, Denise
1. Sport Initiative et Loisir Bleu Association, BP 1810467038 Strasbourg, Cedex, France.
2. Sport Initiative et Loisir Bleu Association, C/ Bailén 11 bajos, 08010 Barcelona, Spain

Word count (excluding title page, abstract, references, figures and tables): 3750

For peer review only

For peer review only - http://bmjopen.bmj.com/site/about/guidelines.xhtml

TITLE:

Exercise referral schemes enhanced by self-management strategies to reduce sedentary behaviour and increase physical activity among community-dwelling older adults from four European countries: protocol for the process evaluation of The SITLESS randomised controlled trial

ABSTRACT

Introduction: SITLESS is a randomised controlled trial determining whether exercise referral schemes can be enhanced by self-management strategies to reduce sedentary behaviour, increase physical activity and improve health, function and quality of life in the long term in community-dwelling older citizens from Spain, UK, Germany and Denmark. The intervention is complex and requires a process evaluation to understand how implementation, causal mechanisms and context shape outcomes. The specific aims are to: assess the implementation (quantity and quality of what was delivered); assess the generalizability of the effectiveness by understanding the role of context and the comparability between intervention sites; understand the mechanisms of impact; and explore perceived impacts.

Methods and analysis: Following the Medical Research Council guidance on complex interventions, it is organized around three categories: implementation, context, and mechanisms of impact. Each aim is addressed complementarily by several methodological procedures. Observation checklists and attendance registry are conducted in each session of physical activity and self-management strategies. Standardized scales (i.e. Marcus's Self-Efficacy Questionnaire, the Physical Activity Self-Regulation Scale, and the Lubben Social Network Scale) are included in the baseline, post-intervention and follow-up assessments. Contextual characteristics are collected in a semi-structured questionnaire. Qualitative procedures comprise participant observation of the sessions, semi-structured interviews and focus groups with participants and trainers at post-intervention and during the follow-up.

For peer review only - http://bmjopen.bmj.com/site/about/guidelines.xhtml

Ethics and dissemination: The study design was approved by the respective Ethical Committee of Ramon Llull University, Southern Denmark, Northern Ireland and Ulm University. Participation is voluntary, and all participants are asked to sign informed consent before starting the study.

A dissemination plan operationalizes how to achieve a social impact by reaching academic and non-academic stakeholders. A Data Management Plan describes the specific data sets and regulates its deposition and curation. All publications will be open access.

Trial registration number: ClinicalTrials.gov, NCT02629666. Registered 19 November 2015.

Key words: Process Assessment (Health Care), Clinical Protocols, Clinical Trial, Health Behaviour, Qualitative Research.

STRENGTHS AND LIMITATIONS OF THIS STUDY:

1. Process evaluation is an essential part of the testing of the SITLESS complex intervention.
2. The Medical Research Council guidance on "Process evaluation of complex interventions", by Moore et al (2015) is used as a framework for conducting and reporting this process evaluation.
3. Mixed methods are used to address the specific aims and each aim is addressed by several methodological procedures applied with a complementary purpose.
4. Important information is identified about the differences and communalities regarding implementation, context and mechanisms of impact of a complex intervention aimed at increasing physical activity and reducing sedentary behaviour across intervention sites in four European countries.
5. Potential limitations are the reduced number of cases interviewed from the control arm, and the lack of in-depth analysis of the experiences of participants with lower adherence to the intervention and of those dropping out of the study.

For peer review only - http://bmjopen.bmj.com/site/about/guidelines.xhtml

INTRODUCTION

Randomized controlled trials (RCTs) have been traditionally focused on outcome evaluations by linking cause and effect and have been criticised as over simplistic for ignoring relevant aspects, such as the crucial role of context in shaping outcomes.[1] Therefore, the guidance on the design and evaluation of RCTs of complex interventions has emphasized the relevance of the processes to provide greater confidence in the conclusions about the effectiveness and thus improve how they inform future intervention development.[2] Complex interventions are composed by multiple active components that might: interact, be directed to change behaviour or provoke actions of intervention participants, target several organisational levels, allow a degree of tailoring or flexibility, and require a level of skills by those delivering and/or receiving the intervention. In addition, the effects of the intervention might be context-dependent or changed by recipient or provider factors and the causal pathway between the intervention and the outcome might be long, variable or include more than one causal pathway.[3]

The Medical Research Council (MRC) has recently proposed guidance to structure the process evaluation of clinical trials of complex interventions.[45] This framework is organized around three categories: i) context, ii) implementation and iii) mechanisms of impact, and considers their mutual relationships. This guideline recommends assessing fidelity and quality of implementation, clarifying causal mechanisms (i.e., to get a better understanding of the complex pathways between the intervention and the outcomes, and to identify unexpected mechanisms) and identifying contextual factors associated that might explain why different results could be found across sites.

In our current ageing societies, promoting physical activity has become one of the key health care policies for the prevention of age-related disability, risk reduction of non-communicable diseases and improvement of quality of life.[6–8] Moreover, sedentary behaviour has been established as a risk factor for mortality which is independent of low physical activity and reducing it, is

For peer review only - http://bmjopen.bmj.com/site/about/guidelines.xhtml

increasingly becoming a relevant public health concern, especially for older adults.[9–12] Therefore, the SITLESS project was designed to determine whether the effects of exercise referral schemes (ERS) may be enhanced by self-management strategies (SMS), to reduce sedentary behaviour and increase physical activity with the final goal of long-term behaviour change, an improvement in health, physical function, quality of life as well as psychosocial outcomes in community-dwelling older European citizens. In order to achieve these goals, SITLESS was designed as a three-armed pragmatic randomised controlled trial, to assess the effectiveness and cost-effectiveness of existing physical activity interventions (ERS) enhanced with SMS, compared with ERS alone and also with general recommendations about physical activity (control group).[1314]

SITLESS is based on an intervention that fulfils the definition of a complex intervention, mainly since it comprises different active components that allow for tailoring to the personal and local characteristics, it is aimed at changing behaviours and has a wide potential range of short and long-term physical, social and psychological effects.[15] Accordingly, the SITLESS intervention was built upon a logic model presented in Figure 1 and the SITLESS consortium decided to design and implement a process evaluation as recommended.

Figure 1. The logic model of the SITLESS intervention.

In addition, the SITLESS study follows the approach of Responsible Research Innovation (RRI), understood as an approach that anticipates and assesses potential implications and societal expectations with regard to research and innovation, with the aim to foster the design of inclusive and sustainable research and innovation.[16]

Therefore, the SITLESS project has designed a process evaluation with the **aim** of understanding how implementation, causal mechanisms and the contextual factors shape the short and long-term outcomes. The specific aims of the process evaluation are to: 1) assess the quantity and quality of what was delivered by detailing the implementation; 2) assess the generalizability of the effectiveness by understanding the role of context and specially exploring the comparability

For peer review only - http://bmjopen.bmj.com/site/about/guidelines.xhtml

between intervention sites, including the variability between countries and between intervention sites within the four European countries involved; 3) understand the mechanisms of impact; and 4) explore perceived impacts.

The specific **research questions**, as shown in **figure 2**, in each objective are:

- **Implementation**: Regarding fidelity: *"Are key components of the SMS and PA intervention implemented and how well are they implemented according to the intervention manual?"* Regarding dose: *"Does the adherence to the intervention of each participant explain the effectiveness?"*
- **Context:** *"How do contextual factors regarding the characteristics of the setting, facilitator, the neighbourhood, health care system, and other country specific aspects affect implementation and outcomes?"*
- **Mechanisms of impact:** *"How does the SMS intervention produce change? How does each intervention component contribute to producing changes in behaviour? Does the SITLESS logic model work in practice? Specifically, does the intervention work by improving self-efficacy and social support? Does better adherence to the components of the intervention mediate the reduction of sedentary behaviour and the increase in physical activity?"*
- **Perceived effects:** "Which effects could be attributed to the intervention as perceived by the participants?"

METHODS AND ANALYSIS

Theoretical frameworks informing this process evaluation

The process evaluation of the SITLESS project follows the guidance on **"Process evaluation of complex interventions"** from the Medical Research Council (MRC).[5] According to this guidance,

For peer review only - http://bmjopen.bmj.com/site/about/guidelines.xhtml

process evaluation is understood as an essential part of the design and testing of the SITLESS intervention.

The SITLESS process evaluation framework

Figure 2 illustrates the application of the MRC guidance to the SITLESS project. This figure highlights the research question in the categories of implementation, context, and mechanisms of impact, and relates them with each methodological procedure applied. In addition, it shows the intervention characteristics and the outcomes.

Figure 2. The process evaluation framework for the SITLESS trial.

Mixed methods used to address the specific aims

A combination of quantitative and qualitative procedures will be applied.

Quantitative procedures quantify the participants' adherence to the intervention and the fidelity to the intervention manual through observation checklists and attendance registries; involve standardized scales to assess mediating factors, and a semi-structured questionnaire on the contextual characteristics that might influence delivery (e.g., orography).

Qualitative procedures address the same research questions but according to participants' perceptions, trainers' experience and researchers' observations. Qualitative procedures include participant observation, semi-structured interviews and focus groups and are detailed in **table 1**.

Qualitative methods require specific considerations regarding the research team, reflexivity and the study design.

A multi-country and multi-disciplinary research team including a variety of health professionals (medical doctors, epidemiologists, exercise physiologists, physiotherapists, etc.) will lead the analysis of the qualitative data gathered in Barcelona, Odense, Ulm and Belfast. All members of the team involved in the qualitative research will be encouraged to keep a research diary to record their reflexive notes, impressions of the data and thoughts about the analysis throughout

For peer review only - http://bmjopen.bmj.com/site/about/guidelines.xhtml

the process. Researchers should remain flexible and adaptive throughout the process and not be attached to certainty to generate rich findings that could explain the complexity of social issues. Researchers with less experience in qualitative research will be trained to code, index, and chart data and to think reflexively about how their identities and experience affect the analysis process in order to help to interpret with accuracy the meaning and significance of the data.

We will apply purposeful sampling of participants with maximal variation as a strategy to explore the differences and communalities regarding gender, functional level, ethnicity and socio-economic background, next to the type of intervention arm (SMS+ERS group, ERS group and control group). Each qualitative procedure targets a specific purposeful sample of participants from each of the four intervention sites (Barcelona, Belfast, Odense and Ulm) according to the characteristics specified in **table 1**. Moreover, several procedures will be applied at different time-points (post-intervention and follow-up) to match with the relevant assessment time-points of the trial. The aims regarding context and mechanisms of impact will be explored at the end of intervention and both follow-up periods, whereas implementation is only assessed at the end of the intervention.

Each aim is addressed by several methodological procedures applied with a complementary purpose as illustrated in Figure 3 and described in detail below.

Figure 3. Research questions and methods adopted for the process evaluation of the SITLESS trial.

Aim 1: to assess the quantity and quality of what was delivered by detailing the implementation of the intervention components.

Methods to evaluate aim 1:

Observation checklists: the observation checklist of the SMS intervention sessions comprises of the one-to-one sessions, the group-based sessions, and the telephone prompts. It requires

For peer review only - http://bmjopen.bmj.com/site/about/guidelines.xhtml

recording of the degree of fulfilment of each task and each aim of all sessions. The observation checklist of the ERS sessions covers the implementation characteristics that might vary across intervention sites or across sessions (e.g., whether the sessions are conducted indoors or outdoors and information about whether and how the trainers tailored the physical activity session to the frail participants and personalized the progression in the exercise intensity). These data are collected by the trainer after each session of all SMS and ERS groups.

Attendance registry: all SMS, ERS and control group sessions have a corresponding attendance registry. It contains each participant's attendance and reasons for not attending. These data are recorded by the trainer after each session of all SMS, ERS and control groups.

Qualitative procedures: semi-structured interviews and focus groups with participants will include questions about their perception of the implementation of the specific intervention elements. Focus groups with trainers will have specific questions on their perception of the implementation challenges. In addition, the participant observation of the SMS and ERS sessions by researchers will focus on the implementation of the intervention.

Aim 2: to assess the generalizability of the effectiveness of the intervention by assessing the role of context and specially exploring the comparability between intervention sites in four European countries.

Methods to evaluate aim 2:

Semi-structured questionnaire: the evaluation of this aim is addressed with a semi-structured questionnaire on contextual characteristics. Specifically, all intervention sites will provide information on the specific intervention setting regarding a specific list of characteristics available to be recorded in a standardized manner:

For peer review only - http://bmjopen.bmj.com/site/about/guidelines.xhtml

- Conditions of the neighbourhood: socio-economic conditions, urban/rural context, topography, public availability of formal or other local opportunities for physical activity (e.g. parks, urban gym).
- Characteristics of the primary health care setting and other settings involved in the recruitment: size in terms of reach of patients, type of patients (e.g. public or private insurance, national health service), type of health professionals involved (e.g. primary care doctors, nurses, physical therapists, social workers), and existing exercise referral schemes in the setting.
- Characteristics of the setting in which the intervention takes place: type of setting (e.g. primary health care, senior club, sports club), relationship of the intervention's setting with the recruitment points (if they differ), outdoor / indoor physical activity.
- Season and adverse climate during the intervention.

Qualitative procedures: semi-structured interviews and focus groups with participants and trainers include specific questions about the context. We will specifically explore the role of the physical environment and social context, with the latter comprising the personal circumstances, the personal network and the group dynamic of the SMS+ERS and ERS intervention arms.

Aim 3: to understand the mechanisms of impact of the intervention.

Methods to evaluate aim 3:

Standardized scales: three standardized scales are included to measure intermediate outcomes that may mediate the relationship between the intervention and changes in physical activity and sedentary behaviour. Specifically, Marcus's Self-Efficacy Questionnaire (MSEQ)[18], the 12-item Physical Activity Self-Regulation Scale (PASR-12)[19] and the Lubben Social Network Scale[20] are assessed within the baseline, post-intervention, and follow-up evaluations. All three scales are applied to explore the role of self-efficacy, self-monitoring, goal setting, eliciting social support, reinforcements, time management, relapse prevention and social network as mediators of the

For peer review only - http://bmjopen.bmj.com/site/about/guidelines.xhtml

decrease in sedentary behaviour and increase on physical activity as predicted by the SITLESS logic model (Figure 1).

Qualitative procedures: we will employ qualitative methods to explore pathways in the intervention logic model not assessed through the standardised questionnaires. Thus, they will be used to get a better understanding of complex causal pathways and unexpected mechanisms between intervention and outcomes. Specifically, semi-structured interviews with participants and focus groups with participants and trainers at the end of the intervention and at follow-up will include questions about perceived effects according to participants' experiences and trainers' observations. In addition, the participant observation of the SMS and ERS sessions by researchers will focus on how participants' actively respond and interact with the intervention.

Aim 4: to explore participants' and trainers' perception of the impact of the intervention.

Methods to evaluate aim 4:

Qualitative procedures: This aim is addressed through semi-structured interviews with participants and focus groups with participants and trainers at the end of the intervention and at follow-up. Perceived impacts will be considered as complementary to results of the effectiveness evaluation assessed with standardised scales.[13]

Using the approach of Responsible Research Innovation (RRI), the SITLESS process evaluation explores if there are differing responses to the intervention by disadvantage categories related with gender, disability, ethnicity, and socio-economic position. It also considers how these categories affect implementation and mechanisms of impact and, consequently, the effectiveness of the intervention.[16]

For peer review only - http://bmjopen.bmj.com/site/about/guidelines.xhtml

Table 1. Specific qualitative procedures, pre-selected criteria for the purposeful sampling,

**Qualitative procedure**	**Target**	**Pre-selected criteria for the purposeful sampling and numbers of each profile in each intervention site**	**Timeframe**
Participant observation	Participants in the SMS group	*Criteria:*<br>1 from low socio-economic neighbourhood (or medium, if no low)<br>1 medium neighbourhood (or high, if no low)	During the SMS intervention
Semi-structured individual interviews	Participants in the SMS group	*Criteria:* (priority low and medium socio-economic status)<br>1 frail man & 1 frail woman<br>1 robust man & 1 robust woman<br>1 for each ethnic minority (if applicable)	End of intervention
		Criteria: 1 man & 1 woman (priority is low-to-medium socio-economic status)	12 months follow-up<br>18 months follow-up
	Participants of the ERS group	*Criteria:* 1 man & 1 woman (priority is low-to-medium socio-economic status)	12 months follow-up<br>18 months follow-up
	Participants of the control group	*Criteria:* (priority is low-to-medium socio-economic status)<br>1 man<br>1 woman	End of intervention<br>12 months follow-up<br>18 months follow-up
Focus groups	Participants of the SMS intervention groups	*Criteria:*<br>1 low socio-economic neighbourhood (or medium, if no low)<br>1 medium neighbourhood (or high, if no low)	End of intervention
	Participants of the ERS intervention groups	1 (priority is low-to-medium socio-economic status)	End of intervention
	ERS and SMS trainers	1 with all trainers involved in the ERS and SMS training.	End of intervention

For peer review only - http://bmjopen.bmj.com/site/about/guidelines.xhtml

Analysis plan

Data from the process evaluation will be analysed according to two main procedures: quantitative and qualitative analyses.

Quantitative analysis:

Quantitative process evaluation data, recorded in the attendance registry and the observation checklists, will be used to conduct a descriptive analysis. The *SITLESS observation checklists* will be used to calculate the percentage of fidelity of the implementation regarding tasks and aims of the SMS and ERS sessions in each intervention site and to identify whether differences may exist. All variables will be analysed considering the overall trial and in subgroup analysis, e.g., considering each intervention site. The adherence to the ERS, SMS and control group sessions will be calculated as a percentage of sessions attended with each respective attendance registry. The adherence is an equivalent concept of dose and considers the compliance with the intervention (e.g., did only part of the exercises due to pain). In addition, this registry will allow estimating the frequency of reasons for not attending.

Specific data will be entered in the main study database. This is the case for the variable "adherence/dose" that will allow us to assess whether a higher dose of the intervention is related with more effect. The MSEQ, the PASR-12 and the Lubben Social Network Scale will be analysed within the main database to test their potential role as mediating the effect of the intervention on physical activity and sedentary behaviour. The information collected in the semi-structured questionnaire on context characteristics will be used to describe each intervention site.

Qualitative analysis:

The SITLESS qualitative data analysis will be based on the transcripts of the semi-structured interviews and focus groups, and the trainers' notes from the participant observation. The Framework Method will be applied to analyse these data.[21] Accordingly, we will apply a

For peer review only - http://bmjopen.bmj.com/site/about/guidelines.xhtml

combined approach to the analysis, enabling themes to be developed both inductively from the accounts (experiences and views) of research participants and deductively from existing literature. Regular team meetings will facilitate our critical exploration of participant responses, discussion of deviant cases and agreement on recurring themes.

The analysis will be conducted following these steps: transcription, familiarisation with the interview, coding of the transcripts and diaries of the participant observation, developing a working analytical framework, applying the analytical framework, charting data into the framework matrix and interpreting the data.

The initial coding of the first four transcripts, one per intervention site, will be conducted by two independent researchers, adding coding labels, notes and ideas. As a next step, a working analytical framework will be developed by discussing codes assigned, similarities and differences to achieve an agreement on a set of codes to establish an initial analytical framework. This framework will be refined by coding of further manuscripts until no new codes are generated by two independent researchers. Next, the final analytical framework will be applied to the remaining transcripts. The use of a software to conduct the analysis will be discussed. Once all the data are coded, we will summarize the data in a matrix for each theme.[21] The matrix will be comprised of one row per participant and one column per code with a separate sheet for each category, using also verbatims into the corresponding cells. To interpret the data, themes are generated from the data set by reviewing the matrix and making connections within and between participants and categories. Lastly, analytical memos and team discussions will be used to develop themes aimed at offering possible explanations for what is happening within the data.

ETHICS AND DISSEMINATION

Ethics

For peer review only - http://bmjopen.bmj.com/site/about/guidelines.xhtml

The study design was approved by the Ethics and Research Committee of each intervention site: The Ethics and Research Committee of Fundació Blanquerna (Ramon Llull University, Spain), The Regional Committees on Health Research Ethics for Southern Denmark (University of Southern Denmark, Denmark), Office for Research Ethics Committees in Northern Ireland (ORECNI) (Queen's University of Belfast) and the Ethical Review Board of Ulm University (Ulm, Germany). Participation is voluntary and all participants will be asked to sign informed consent before the start of the study.

Dissemination

The SITLESS dissemination plan operationalizes how to accomplish the main dissemination goal, which is to achieve a social impact by reaching academic and non-academic stakeholders, i.e., primary care and health care professionals, older adults, policy makers, professionals in fields related to physical activity promotion, etc. Specifically, process evaluation results will enhance social impact by providing implementation details that might be of interest for policy makers. In addition, qualitative results (e.g., quotations of participants) will support reaching non-academic stakeholders. A Data Management Plan has been developed for the SITLESS study describing each specific dataset in the study and regulating its deposition and curation. Quantitative data are entered into an on-line platform database. Qualitative data are locally stored in each intervention site (e.g., audio recordings) and also deposited centrally (e.g., transcripts) by the partner leading the analysis (FSIE).

All publications from the SITLESS project, including those related with process evaluation, will be open access.

Conclusions

The SITLESS project has developed a process evaluation to understand how implementation, causal mechanisms and the contextual factors shape the short and long-term outcomes of a complex intervention that enhance ERS with SMS to reduce sedentary behaviour and increase

For peer review only - http://bmjopen.bmj.com/site/about/guidelines.xhtml

For peer review only

physical activity among community dwelling older adults of four European countries. Accordingly, this RCT has the purpose of facilitating future intervention development by informing policy-makers, practitioners and researchers about implementation, mechanisms of impact and contextual factors in detail. In case the intervention is proven as being effective and cost-effective, the information will be focused on the actually delivered intervention to allow replication in new contexts considering the relevant core components, context characteristics and implementation challenges. In case of being ineffective, process evaluation results will contribute to define whether the failure can be attributed to the intervention itself or to a poor implementation. Hence, process evaluation will provide useful data for systematic reviews to compare interventions considering their implementation and contextual characteristics.

Trial status

This initial phase of this project commenced in May 2015. A feasibility study was performed from December 2015 until June 2016. The recruitment for the trial started in July 2016. At the time of submission of this protocol, all 1390 participants have been included in the study. To date, follow-up assessments are being conducted and are planned to finish by April 2019.

Author Contributions:

LCP, MT, MGG, PC, EM and DR contributed to the conception of the study. LCP, SB, KW, MS and NB contributed to the development of the specific procedures.

LCP will coordinate the overall development of the process evaluation. LCP, KW, MS and NB will coordinate the process evaluation in their respective intervention sites and LCP, SB, KW, MS and NB will contribute to the acquisition of process evaluation data.

LCP, SB, MT, PC, MGG, CM, FK, MSk, KW, NB, MDeid, EM, DR, RGR, JJ, OS, MSa; JW, MG, DG, MDenk, AS and MR contributed to the design of the final process evaluation and will contribute to the analysis and interpretation of data and writing of the manuscript. LCP and MR contributed to the data management plan and analysis.

All authors provided feedback on the drafts of this paper and have read and approved the final manuscript.

For peer review only - http://bmjopen.bmj.com/site/about/guidelines.xhtml

Acknowledgements

Data statement: Not applicable.

Funding:

This work was supported by the European Union program Horizon 2020 with the grant number H2020-Grant 634270.

Competing interests:

The authors declare that they have no competing interests.

Ethics approval:

The study design was approved by the Ethics and Research Committee of each intervention site: The Ethics and Research Committee of Ramon Llull University (reference number: 1314001P) (Fundació Blanquerna, Spain), The Regional Committees on Health Research Ethics for Southern Denmark (reference number: S-20150186) (University of Southern Denmark, Denmark), Office for Research Ethics Committees Northern Ireland (ORECNI reference number: 16/NI/0185) (Queen's University of Belfast) and the Ethical Review Board of Ulm University (reference number: 354/15) (Ulm, Germany). Participation is voluntary and all participants will be asked to give written informed consent before the start of the study.

Open access

Not applicable.

For peer review only

REFERENCES

1. Berwick, D. M. The science of improvement. *JAMA - Journal of the American Medical Association* **299,** 1182–1184 (2008).

2. Campbell, N. C. *et al.* Designing and evaluating complex interventions to improve health care. *BMJ* **334,** 455–9 (2007).

3. Campbell, N. C. *et al.* Designing and evaluating complex interventions to improve health care. *BMJ* **334,** 455–9 (2007).

4. Moore, G., Audrey, S., Barker, M. & Bond, L. Process evaluation of complex interventions. *UK Med. Res. Counc. Guid. Prep.* 19-45; 64-75 (2014). doi:http://www.populationhealthsciences.org/MRC-PHSRN-Process-evaluation-guidance-final-2-.pdf

5. Moore, G. F. *et al.* Process evaluation of complex interventions: Medical Research Council guidance. *BMJ Br. Med. J.* **350,** 1–7 (2015).

6. Lee, I. M. *et al.* Effect of physical inactivity on major non-communicable diseases worldwide: An analysis of burden of disease and life expectancy. *Lancet* **380,** 219–229 (2012).

7. Who, W. H. O. Global recommendations on physical activity for health. *Geneva World Heal. Organ.* 60 (2010). doi:10.1080/11026480410034349

8. Ding, D. *et al.* The economic burden of physical inactivity: a global analysis of major non-communicable diseases. *Lancet* **388,** 1311–1324 (2016).

9. Owen, N., Sparling, P. B., Healy, G. N., Dunstan, D. W. & Matthews, C. E. Sedentary behavior: Emerging evidence for a new health risk. *Mayo Clinic Proceedings* **85,** 1138–1141 (2010).

10. Koster, A. *et al.* Association of Sedentary time with mortality independent of moderate to vigorous physical activity. *PLoS One* **7,** (2012).

11. Harvey, J. A., Chastin, S. F. M. & Skelton, D. A. Prevalence of sedentary behavior in older adults: a systematic review. *Int. J. Environ. Res. Public Health* **10,** 6645–61 (2013).

12. Ekelund, U. *et al.* Does physical activity attenuate, or even eliminate, the detrimental association of sitting time with mortality? A harmonised meta-analysis of data from more than 1 million men and women. *Lancet* **388,** 1302–1310 (2016).

13. Giné-Garriga, M. *et al.* The SITLESS project: Exercise referral schemes enhanced by self-management strategies to battle sedentary behaviour in older adults: Study protocol for a randomised controlled trial. *Trials* **18,** (2017).

14. Deidda, M. & Coll-Planas, L. Cost effectiveness of exercise referral schemes enhanced by self-management strategies to battle sedentary behaviour in older adults: protocol for an economic evaluation alongside the SITLESS three-armed pragmatic randomised clinical trial. *BMJ Open* (2018).

15. Lewin, S. *et al.* Assessing the complexity of interventions within systematic reviews:

For peer review only - http://bmjopen.bmj.com/site/about/guidelines.xhtml

Development, content and use of a new tool (iCAT-SR). *BMC Med. Res. Methodol.* **17,** 1–13 (2017).

16. Bardone, E. & Lind, M. Towards a phronetic space for responsible research (and innovation). *Life Sci. Soc. Policy* **12,** 5 (2016).

17. Marcus, B. & Forsyth, L. *Motivating people to be physically active*. (Human Kinetics, 2009).

18. Marcus, B. H., Selby, V. C., Nlaura, R. S. & Rossi, J. S. Self-efficacy and the stages of exercise behavior change. *Res. Q. Exerc. Sport* **63,** 60–66 (1992).

19. Umstattd, M. R., Motl, R., Wilcox, S., Saunders, R. & Watford, M. Measuring physical activity self-regulation strategies in older adults. *J. Phys. Act. Health* **6 Suppl 1,** S105-12 (2009).

20. Lubben, J. E. Assessing social networks among elderly populations. *Fam. Community Health* **11,** 42–52 (1988).

21. Gale, N. K., Heath, G., Cameron, E., Rashid, S. & Redwood, S. Using the framework method for the analysis of qualitative data in multi-disciplinary health research. *BMC Med. Res. Methodol.* **13,** 117 (2013).

Figure's legends:

Figure 1. The logic model of the SITLESS intervention.

Figure 2. The process evaluation framework for the SITLESS trial.

**** IDEA:** refers to the IDEA problem solving framework. [17]

Figure 3. Research questions and methods adopted for the process evaluation of the SITLESS trial.

For peer review only

For peer review only - http://bmjopen.bmj.com/site/about/guidelines.xhtml

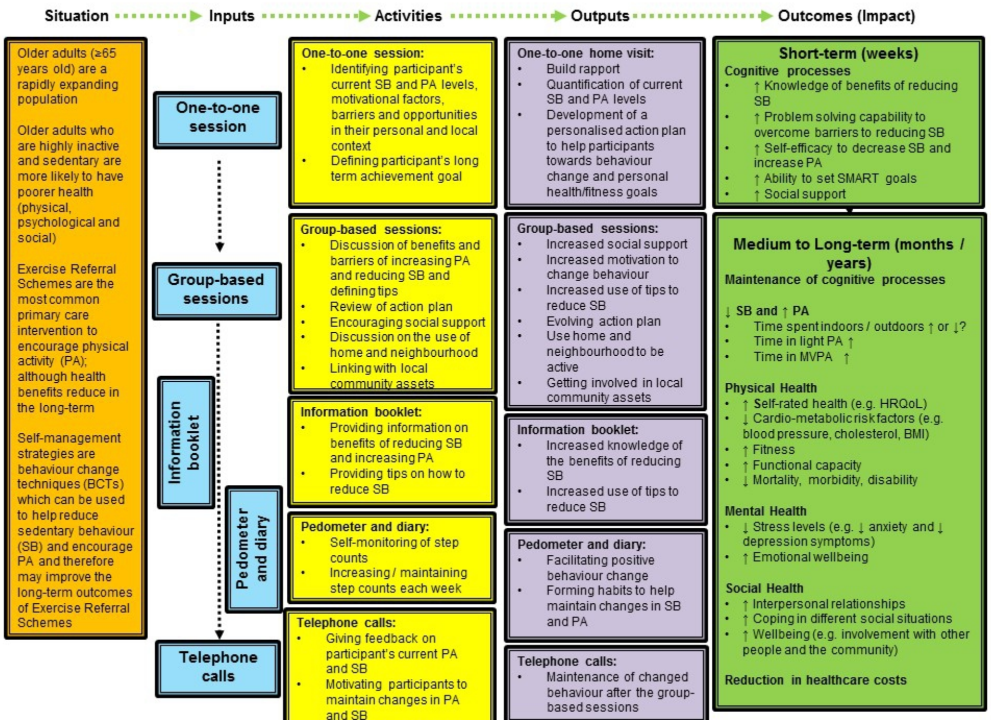

Figure 1. The logic model of the SITLESS intervention.

81x60mm (300 x 300 DPI)

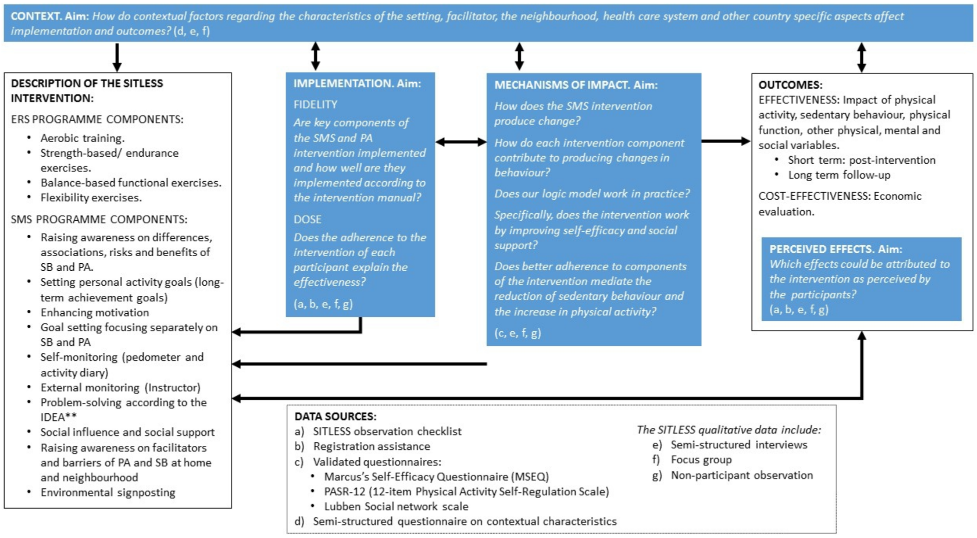

Figure 2. The process evaluation framework for the SITLESS trial.
** IDEA: refers to the IDEA problem solving framework. 17

108x60mm (300 x 300 DPI)

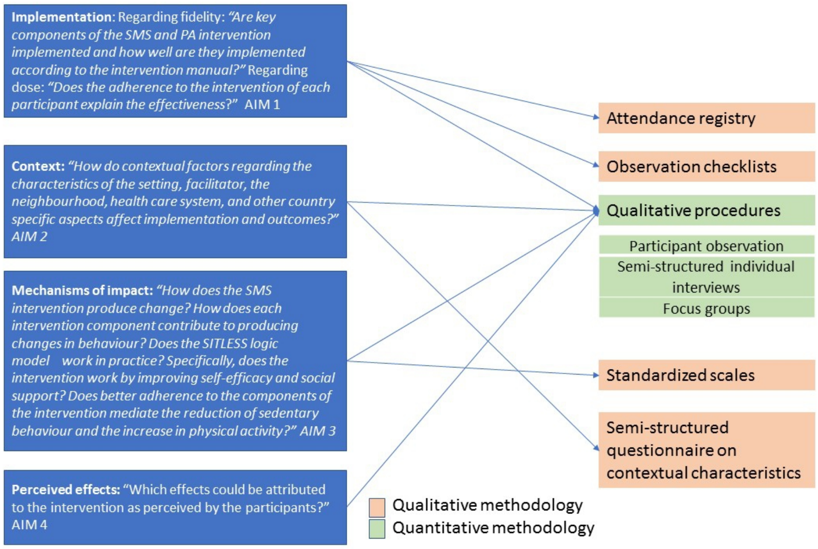

Figure 3. Research questions and methods adopted for the process evaluation of the SITLESS trial.

108x60mm (300 x 300 DPI)

BMJ Open

Exercise referral schemes enhanced by self-management strategies to reduce sedentary behaviour and increase physical activity among community-dwelling older adults from four European countries: protocol for the process evaluation of The SITLESS randomised controlled trial

Journal:	*BMJ Open*
Manuscript ID	bmjopen-2018-027073.R1
Article Type:	Protocol
Date Submitted by the Author:	21-Dec-2018
Complete List of Authors:	Coll-Planas, Laura; Universitat Autònoma de Barcelona, Fundació Salut i Envelliment (Foundation on Health and Ageing)- UAB; Institute of Biomedical Research (IIB Sant Pau)<br>Blancafort Alias, Sergi; Universitat Autònoma de Barcelona, Fundació Salut i Envelliment (Foundation on Health and Ageing)- UAB; Institute of Biomedical Research (IIB Sant Pau)<br>Tully, Mark; Ulster University, Institute of Mental Health Sciences, School of Health Sciences; Queen's University Belfast, 2. UKCRC Centre of Excellence for Public Health Research (Northern Ireland)<br>Caserotti, Paolo; Syddansk Universitet, Department of Sports Science and Clinical Biomechanics, Center for Active and Healthy Ageing (CAHA)<br>Giné-Garriga, Maria; Universitat Ramon Llull, Facultat de Psicologia, Ciències de l'Educació i de l'Esport Blanquerna; Glasgow Caledonian University, School of Health and Life Sciences<br>Blackburn, Nicole; Queen's University Belfast School of Medicine Dentistry and Biomedical Sciences, UKCRC Centre of Excellence for Public Health (NI), Centre for Public Health<br>Skjødt, Mathias; Syddansk Universitet , Department of Sports Science and Clinical Biomechanics, Center for Active and Healthy Ageing (CAHA)<br>Wirth, Katharina ; Universitat Ulm, Institute of Epidemiology and Medical Biometry; Agaplesion Bethesda Clinic, Geriatric Research Unit Ulm University and Geriatric Center Ulm Alb-Donau,<br>Deidda, Manuela; University of Glasgow, Health Economics and Health Technology assessment<br>McIntosh, Emma; University of Glasgow Institute of Health and Wellbeing, Health Economics and Health Technology Assessment (HEHTA)<br>Rothenbacher, Dietrich; Ulm University, Institute of Epidemiology and Medical Biometry<br>Gallardo Rodríguez, Rodrigo; Universidad Catolica de la Santisima Concepcion, Departamento de Ciencias del Deporte y Acondicionamiento Físico, CEADE<br>Jerez-Roig, J.; Universitat de Vic - Universitat Central de Catalunya, 2. Research group on Methodology, Methods, Models and Outcomes of Health and Social Sciences (M3O). Faculty of Health Science and Welfare. Centre for Health and Social Care Research (CESS). ; Universitat Ramon Llull, 1. Facultat de Psicologia, Ciències de l'Educació i

	de l'Esport Blanquerna<br>Sansano-Nadal, Oriol; Universitat Ramon Llull, Facultat de Psicologia, Ciències de l'Educació i de l'Esport Blanquerna<br>Santiago, Marta; Universitat Ramon Llull, Facultat de Psicologia, Ciències de l'Educació i de l'Esport Blanquerna<br>Wilson, Jason; Queen's University Belfast School of Medicine Dentistry and Biomedical Sciences, Centre for Public Health<br>Guerra-Balic, Míriam ; Universitat Ramon Llull, Facultat de Psicologia, Ciències de l'Educació i de l'Esport Blanquerna<br>Martín-Borràs, Carme; Faculty of Psychology, Education and Sport Sciences (FPCEE) Blanquerna, Ramon Llull University., Department of Physical Activity and Sport Sciences; Faculty of Health Sciences (FCS) Blanquerna, Ramon Llull Univesity., Department of Physical Therapy<br>Gonzalez, Denise; Sport Initiative et Loisir Bleu Association; Sport Initiative et Loisir Bleu Association<br>Denkinger, Michael; Universitat Ulm, Agaplesion Bethesda Clinic, Geriatric Research Unit Ulm University and Geriatric Center Ulm Alb-Donau<br>Kee, Frank; Queen's University Belfast, UKCRC Centre of Excellence for Public Health (NI)<br>Salvà Casanovas, Antoni ; Universitat autonoma de Barcelona, Fundació Salut i Envelliment (Foundation on Health and Ageing)- UAB; Institute of Biomedical Research (IIB Sant Pau)<br>Roqué i Figuls , Marta; Universitat Autònoma de Barcelona, Fundació Salut i Envelliment (Foundation on Health and Ageing)- UAB; Institute of Biomedical Research (IIB Sant Pau)

**Primary Subject Heading**:	Public health
Secondary Subject Heading:	Research methods
Keywords:	EPIDEMIOLOGY, PUBLIC HEALTH, GERIATRIC MEDICINE

SCHOLARONE™
Manuscripts

TITLE PAGE:

The title page must contain the following information:

- Title of the article.

Exercise referral schemes enhanced by self-management strategies to reduce sedentary behaviour and increase physical activity among community-dwelling older adults from four European countries: protocol for the process evaluation of The SITLESS randomised controlled trial

Authors: **Coll-Planas, Laura; Blancafort, Sergi; Tully, Mark A; Caserotti, Paolo; Giné-Garriga, Maria; Blackburn, Nicole E; Skjødt, Mathias; Wirth, Katharina; Deidda, Manuela; McIntosh, Emma; Rothenbacher, Dietrich; Gallardo Rodríguez, Rodrigo; Jerez-Roig, Javier; Sansano-Nadal, Oriol; Santiago, Marta; Wilson, Jason J; Guerra-Balic, Miriam; Martín-Borràs, Carme; González, Denise; Denkinger, Michael; Kee, Frank; Salvà, Antoni; Roqué i Figuls, Marta.**

- Full name, postal address, e-mail and telephone number of the **corresponding author.**

Full name: Laura Coll i Planas
Postal address: Fundació Salut i Envelliment (Foundation on Health and Ageing)- Universitat Autònoma de Barcelona
Sant Antoni Maria Claret, 171 E-08041
Barcelona
E-mail: laura.coll@uab.cat

Tel.: +34 93 446 45 06

- Full name, department, institution, city and country of all co-authors.

Coll-Planas, Laura
Blancafort, Sergi
Salvà, Antoni
Roqué i Figuls, Marta.

1. Fundació Salut i Envelliment (Foundation on Health and Ageing)- Universitat Autònoma de Barcelona
   Barcelona, Spain
2. Institute of Biomedical Research (IIB Sant Pau), Barcelona, Spain

Tully, Mark A

1. Institute of Mental Health Sciences, School of Health Sciences, Ulster University, Newtownabbey, Northern Ireland, UK.
2. UKCRC Centre of Excellence for Public Health Research (Northern Ireland), Queen's University Belfast, Belfast, Northern Ireland, UK.

For peer review only - http://bmjopen.bmj.com/site/about/guidelines.xhtml

Blackburn, Nicole E
Wilson, Jason J;
Kee, Frank
UKCRC Centre of Excellence for Public Health (NI), Centre for Public Health, School of Medicine, Dentistry and Biomedical Sciences, Queen's University Belfast, Belfast, UK.

Caserotti, Paolo
Skjodt, Mathias
Department of Sports Science and Clinical Biomechanics, Center for Active and Healthy Ageing (CAHA), University of Southern Denmark, Odense, Campusvej 55, 5230 Odense M, Denmark

Giné-Garriga, Maria
1. Facultat de Psicologia, Ciències de l'Educació i de l'Esport Blanquerna, Universitat Ramon Llull, C/ Císter 34, 08022 Barcelona, Spain
2. School of Health and Life Sciences, Glasgow Caledonian University, Cowcaddens Road, Glasgow, United Kingdom

Sansano-Nadal, Oriol
Santiago, Marta
Guerra, Míriam
Martín-Borràs, Carme
Facultat de Psicologia, Ciències de l'Educació i de l'Esport Blanquerna, Universitat Ramon Llull, C/ Císter 34, 08022 Barcelona, Spain

Jerez-Roig, Javier
1. Facultat de Psicologia, Ciències de l'Educació i de l'Esport Blanquerna, Universitat Ramon Llull, C/ Císter 34, 08022 Barcelona, Spain
2. Research group on Methodology, Methods, Models and Outcomes of Health and Social Sciences (M3O). Faculty of Health Science and Welfare. Centre for Health and Social Care Research (CESS). University of Vic-Central University of Catalonia (UVIC-UCC), C.Sagrada Família, 7, 08500 Vic, Spain

Rothenbacher, Dietrich
Institute of Epidemiology and Medical Biometry, Ulm University, 89081 Ulm, Germany.

Wirth, Katharina
1. Institute of Epidemiology and Medical Biometry, Ulm University, 89081 Ulm, Germany
2. Agaplesion Bethesda Clinic, Geriatric Research Unit Ulm University and Geriatric Center Ulm Alb-Donau, Zollernring 26, 89073 Ulm, Germany

Denkinger, Michael;
Agaplesion Bethesda Clinic, Geriatric Research Unit Ulm University and Geriatric Center Ulm Alb-Donau, Zollernring 26, 89073 Ulm, Germany

For peer review only

For peer review only - http://bmjopen.bmj.com/site/about/guidelines.xhtml

Deidda, Manuela
McIntosh, Emma
Health Economics and Health Technology Assessment (HEHTA), Institute of Health and Wellbeing (IHW), University of Glasgow, 1 Lilybank Gardens, Glasgow G12 8RZ, UK.

Gallardo Rodríguez, Rodrigo
Departamento de Ciencias del Deporte y Acondicionamiento Físico, CEADE, Universidad Católica de la Santísima Concepción, Concepción, Chile.

González, Denise
1. Sport Initiative et Loisir Bleu Association, BP 1810467038 Strasbourg, Cedex, France.
2. Sport Initiative et Loisir Bleu Association, C/ Bailén 11 bajos, 08010 Barcelona, Spain

Word count (excluding title page, abstract, references, figures and tables): 3999

For peer review only - http://bmjopen.bmj.com/site/about/guidelines.xhtml

TITLE:

Exercise referral schemes enhanced by self-management strategies to reduce sedentary behaviour and increase physical activity among community-dwelling older adults from four European countries: protocol for the process evaluation of the SITLESS randomised controlled trial

ABSTRACT

Introduction: SITLESS is a randomised controlled trial determining whether exercise referral schemes can be enhanced by self-management strategies to reduce sedentary behaviour, and increase physical activity in the long term in community-dwelling older citizens. The intervention is complex and requires a process evaluation to understand how implementation, causal mechanisms and context shape outcomes. The specific aims are to: assess fidelity and reach of the implementation; understand the contextual aspects of each intervention site; understand the mechanisms of impact; and explore perceived impacts.

Methods and analysis: Following the Medical Research Council guidance on complex interventions, a combination of qualitative and quantitative procedures are applied: observation checklists and attendance registry of the interventions, standardized scales (i.e. Marcus's Self-Efficacy Questionnaire, Physical Activity Self-Regulation Scale and the Lubben Social Network Scale) at baseline, post-intervention and follow-up assessments, semi-structured questionnaire on contextual characteristics and participant observation of the sessions, semi-structured interviews and focus groups with participants and trainers at post-intervention and during the follow-up. Quantitative outcomes on are analysed as moderators with the impact evaluation outcomes. Descriptive results on context and perceived effects complement impact results. Qualitative results and the overall quantitative results will help to refine the logic model to finally support the interpretation of the results on the effectiveness of the intervention.

For peer review only

Ethics and dissemination: The study design was approved by the respective Ethical Committee of Ramon Llull University, Southern Denmark, Northern Ireland and Ulm University. Participation is voluntary, and all participants are asked to sign informed consent before starting the study.

A dissemination plan operationalizes how to achieve a social impact by reaching academic and non-academic stakeholders. A Data Management Plan describes the specific data sets and regulates its deposition and curation. All publications will be open access.

Trial registration number: ClinicalTrials.gov, NCT02629666. Registered 19 November 2015.

Key words: Process Assessment (Health Care), Clinical Protocols, Clinical Trial, Health Behaviour, Qualitative Research.

STRENGTHS AND LIMITATIONS OF THIS STUDY:

1. Process evaluation is an essential part of the testing of the SITLESS complex intervention.
2. The Medical Research Council guidance on "Process evaluation of complex interventions", by Moore et al (2015) is used as a framework for conducting and reporting this process evaluation.
3. Mixed methods are used to address the specific aims and each aim is addressed by several methodological procedures applied with a complementary purpose.
4. Important information is identified about the differences and communalities regarding implementation, context and mechanisms of impact of a complex intervention aimed at increasing physical activity and reducing sedentary behaviour across intervention sites in four European countries.
5. Potential limitations are the reduced number of cases interviewed from the control arm, and the lack of in-depth analysis of the experiences of participants with lower adherence to the intervention and of those dropping out of the study.

For peer review only - http://bmjopen.bmj.com/site/about/guidelines.xhtml

INTRODUCTION

Randomized controlled trials have been traditionally focused on outcome evaluations by linking cause and effect and have been criticised as over simplistic for ignoring relevant aspects, such as the crucial role of context in shaping outcomes.[1] Therefore, the guidance on the design and evaluation of trials of complex interventions has emphasized the relevance of the processes to provide greater confidence in the conclusions about the effectiveness and thus improve how they inform future intervention development.[2] Complex interventions are composed by multiple active components that might for instance interact, be directed to change behaviour, allow a degree of tailoring or require a level of skills by those delivering and/or receiving the intervention. In addition, the effects of the intervention might be context-dependent and the causal pathway between the intervention and the outcome might be long, variable or include more than one causal pathway.[3]

The Medical Research Council (MRC) has recently proposed guidance to structure the process evaluation of clinical trials of complex interventions.[4,5] This framework is organized around three categories: i) context, ii) implementation and iii) mechanisms of impact, and considers their mutual relationships. This guideline recommends assessing fidelity and quality of implementation, clarifying causal mechanisms (i.e., to get a better understanding of the complex pathways between the intervention and the outcomes, and to identify unexpected mechanisms) and identifying contextual factors associated that might explain why different results could be found across sites. Likewise, the Context and Implementation of Complex Interventions (CICI) framework has been proposed to assess context in detail.[6] Accordingly, context is defined as *anything external to the intervention that may act as a barrier or facilitator to its implementation, or its effect*.[6] Complex interventions work by introducing mechanisms that are sufficiently suited to their context to produce change, while causes of problems targeted by interventions may differ from one context to another. The CICI framework comprises three dimensions: context, implementation and setting, which interact with one another and with the intervention dimension. Hence, *context* comprises

For peer review only - http://bmjopen.bmj.com/site/about/guidelines.xhtml

seven domains: geographical, epidemiological, socio-cultural, socio-economic, ethical, legal and political. Whereas *setting* refers to the specific physical location, in which the intervention is put into practise.

In our current ageing societies, promoting physical activity has become one of the key health care policies for the prevention of age-related disability, risk reduction of non-communicable diseases and improvement of quality of life.[7–9] Moreover, sedentary behaviour has been established as a risk factor for mortality which is independent of low physical activity and reducing it, is increasingly becoming a relevant public health concern, especially for older adults.[10–13] Therefore, the SITLESS project was designed to determine whether the effects of exercise referral schemes (ERS) may be enhanced by self-management strategies (SMS), to reduce sedentary behaviour and increase physical activity with the final goal of long-term behaviour change, an improvement in health, physical function, quality of life as well as psychosocial outcomes in community-dwelling older European citizens. In order to achieve these goals, SITLESS was designed as a three-armed pragmatic randomised controlled trial, to assess the effectiveness and cost-effectiveness of existing physical activity interventions (ERS) enhanced with SMS, compared with ERS alone and also with general recommendations about physical activity (control group).[14,15] The ERS intervention aims to improve strength, aerobic capacity, and balance during 16 weeks, with two 60-minute sessions per week. The SMS consists of one one-to-one session, six group-based sessions and four telephone prompts to encourage behaviour change. Participants are followed-up for 12 and 18-months post-intervention. The control group receives two sessions consisting of general information and recommendations on healthy ageing. A total of 1,338 participants are expected to be included (446 in each arm).

The SITLESS intervention fulfils the definition of a complex intervention, mainly since it comprises different active components that allow for tailoring to the personal and local characteristics, it is aimed at changing behaviours and has a wide potential range of short and long-term physical, social

For peer review only - http://bmjopen.bmj.com/site/about/guidelines.xhtml

and psychological effects.[16] Accordingly, it was built upon a logic model presented in Figure 1 and the SITLESS trial requires a process evaluation as recommended.

Figure 1. The logic model of the SITLESS intervention.

In addition, for the SITLESS study, conducted in the European research framework Horizon 2020, it is a key issue to use the approach of Responsible Research Innovation (RRI). RRI is understood as an approach that anticipates and assesses potential implications and societal expectations with regard to research and innovation, with the aim to foster the design of inclusive and sustainable research and innovation.[17] RRI has been defined as an attempt to re-think research as a public good and thus conducted with and for society;.[17] In practice, the SITLESS project has considered specifically public involvement, gender, ethics, open access and governance along the project.

Therefore, the SITLESS project has designed a process evaluation with the aim of understanding how implementation, causal mechanisms and the contextual factors shape the short and long-term outcomes. Primary outcomes of the trial include: (1) Sedentary behavior as sitting time and time spent in activities which have been traditionally reported as requiring ≤ 1.5 MET, (2) physical activity as daily counts-per-minute and intensity of exercise performed, both objectively measured by ActiGraph® accelerometer. Furthermore, secondary outcomes include other health-related aspects, quality of life, function and psychosocial outcomes at post-intervention and 12-and 18-months follow-up. The specific aims of the process evaluation are firstly to assess the implementation of the intervention in terms of: a) fidelity to the intervention design, variability of what was delivered and how between intervention sites; and b) reach (adherence) of participants to the intervention. Secondly to, understand the role of context as following: a) exploring whether contextual factors regarding the characteristics of the setting, facilitator, the neighbourhood, health care system, and other location specific aspects affect implementation and outcomes; b) assessing the variability and comparability between and within intervention sites; c) assessing the generalizability of the intervention's effectiveness results. Thirdly the SITLESS project aims to,

For peer review only - http://bmjopen.bmj.com/site/about/guidelines.xhtml

understand the mechanisms of impact. Fourth and finally it aims to explore perceived impacts of the intervention.

The specific research questions corresponding to each aim are shown in **figure 2**.

Figure 2. The process evaluation framework for the SITLESS trial.

Figure 2 Legend: **** IDEA:** refers to the IDEA problem solving framework. [18]

METHODS AND ANALYSIS

Theoretical frameworks informing this process evaluation

The process evaluation of the SITLESS project follows the guidance on "Process evaluation of complex interventions" from the MRC.[5] According to this guidance, process evaluation is understood as an essential part of the design and testing of the SITLESS intervention. In addition, context will be specifically evaluated according to the CICI framework.[6]

The SITLESS process evaluation framework

Figure 2 illustrates the application of the MRC guidance to the SITLESS project. This figure highlights the research question in the categories of implementation, context, mechanisms of impact and perceived effects, and relates them with each methodological procedure applied. In addition, it shows the intervention characteristics and the outcomes.

Mixed methods used to address the specific aims

A combination of quantitative and qualitative procedures will be applied. This process evaluation was planned prior to implementation of the intervention, except for the semi-structured questionnaire on contextual data that was added more recently. Nevertheless, observation checklists, attendance list and guidance for the qualitative procedures were tested with the first participants and improved accordingly to ease their use and capture most relevant data.

For peer review only - http://bmjopen.bmj.com/site/about/guidelines.xhtml

Quantitative procedures quantify the fidelity to the intervention manual and the participants' adherence to the intervention through observation checklists and attendance registries, respectively. They also involve standardized scales to assess mediating factors (i.e., Marcus's Self-Efficacy Questionnaire (MSEQ)[19], the 12-item Physical Activity Self-Regulation Scale (PASR-12)[20] and the Lubben Social Network Scale (LSNS)[21]). Hence, contextual characteristics will be collected in a table format with prespecified topics (e.g., geographical aspects).

Qualitative procedures address the same research questions but according to participants' perceptions, trainers' experience and researchers' observations. Qualitative procedures include participant observation, semi-structured interviews and focus groups and are detailed in **table 1**.

Qualitative methods require specific considerations regarding the research team, reflexivity and the study design.

A multi-country and multi-disciplinary research team including a variety of health professionals (medical doctors, epidemiologists, exercise physiologists, physiotherapists, etc.) will lead the analysis of the qualitative data gathered in Barcelona, Odense, Ulm and Belfast. All members of the team involved in the qualitative research will be encouraged to keep a research diary to record their reflexive notes, impressions of the data and thoughts about the analysis throughout the process. Researchers should remain flexible and adaptive throughout the process and not be attached to certainty to generate rich findings that could explain the complexity of social issues. Researchers with less experience in qualitative research will be trained to code, index, and chart data and to think reflexively about how their identities and experience affect the analysis process in order to help to interpret with accuracy the meaning and significance of the data.

We will apply purposeful sampling of participants with maximum variation sampling method as a strategy to select a small number of cases that maximize the diversity relevant to the research question, in this case to explore the differences and communalities regarding gender, functional level, ethnicity and socio-economic background, next to the type of intervention arm (SMS+ERS

For peer review only - http://bmjopen.bmj.com/site/about/guidelines.xhtml

group, ERS group and control group). Each qualitative procedure targets a specific purposeful sample of participants from each of the four intervention sites (Barcelona, Belfast, Odense and Ulm) according to the characteristics specified in **table 1**. Moreover, several procedures will be applied at different time-points (post-intervention and follow-up) to match with the relevant assessment time-points of the trial. The aims regarding context, mechanisms of impact and perceived effects will be explored at the end of intervention and both follow-up periods, whereas implementation is only assessed at the end of the intervention.

Appendixes 1 to 6 show examples of the templates used as observation checklist and attendance list, and guidance for the qualitative procedures (semi-structured interviews and focus groups).

Each aim is addressed by several methodological procedures applied with a complementary purpose as illustrated in Figure 3 and described in detail below.

Figure 3. *Specific aims, procedures and outcomes of the process evaluation and their triangulation with impact evaluation outcomes*

Methods to evaluate aim 1:

Observation checklists: after each session of all SMS and ERS groups, the trainer records the degree of fulfilment of each task and each aim. Hence, the observation checklist of the ERS sessions also covers the implementation characteristics that might vary across intervention sites or across sessions (e.g., whether the sessions are conducted indoors or outdoors and whether and how the trainers tailored the ERS sessions to the frail participants).

Attendance registry: all SMS, ERS and control group sessions have a corresponding attendance registry recorded by the trainer after each session. It contains each participant's attendance, reasons for not attending and adverse events due to the intervention. The attendance is used to calculate the adherence as an equivalent concept of dose and considers the compliance with the intervention (e.g., did only part of the exercises due to pain).

For peer review only - http://bmjopen.bmj.com/site/about/guidelines.xhtml

Qualitative procedures: semi-structured interviews and focus groups with participants will include questions about their perception of the implementation of the specific intervention elements. Focus groups with trainers will have specific questions on their perception of the implementation challenges. In addition, the participant observation of the SMS and ERS sessions by researchers will focus on the implementation of the intervention.

Methods to evaluate aim 2:

Prespecified contextual characteristics: the evaluation of this aim is addressed with a table with prespecified domains of context. Specifically, all intervention sites will provide information on the specific intervention setting and context. Setting characteristics will comprise of: Neighbourhood socioeconomic level (low/medium/high), urban/semi-rural/rural area, type of centers where the recruitment took place, ERS intervention, SMS intervention and the control group intervention takes place, existing ERS in the setting. This information will be collected by researchers of each intervention site. Data on context will include those geographical, epidemiological, socio-cultural, socio-economic, ethical, legal and political information that might be relevant as a barrier or facilitator to the intervention implementation, or its effect, for instance existing national programs on physical activity. These contextual data will be firstly collected by the leaders of each intervention site and complemented with the knowledge and views of the members of each local advisory board.

Qualitative procedures: semi-structured interviews and focus groups with participants and trainers include specific questions about the context. We will specifically explore the role of the physical environment and social context, with the latter comprising the personal circumstances, the personal network and the group dynamic of the SMS+ERS and ERS intervention arms.

Methods to evaluate aim 3:

For peer review only - http://bmjopen.bmj.com/site/about/guidelines.xhtml

Standardized scales: the MSEQ[19], PASR-12[20] and LSNS[21] are assessed within the baseline, post-intervention, and follow-up evaluations , to explore the role of self-efficacy, self-monitoring, goal setting, eliciting social support, reinforcements, time management, relapse prevention and social network as mediators of the decrease in sedentary behaviour and increase on physical activity as predicted by the SITLESS logic model (Figure 1). MSEQ has been shown to be a reliable, valid and accurate measure of self-efficacy for physical activity participation in populations with chronic diseases.[22,23] The PASR-12 has been shown to be a reliable and valid scale for the measurement of self-regulation for physical activity among older adults.[20] The LSNS is commonly used as a measure of social networks. It has high levels of internal consistency, stable factor structures, high correlations with criterion variables and clinical cut-points show good convergent validity among European community-dwelling older adults.[24]

Qualitative procedures: will be used to get a better understanding of complex causal pathways and unexpected mechanisms between intervention and outcomes. Specifically, semi-structured interviews with participants and focus groups with participants and trainers at the end of the intervention and at follow-up will include questions about perceived effects according to participants' experiences and trainers' observations. In addition, the participant observation of the SMS and ERS sessions by researchers will focus on how participants' actively respond and interact with the intervention.

Methods to evaluate aim 4:

Qualitative procedures: This aim is addressed through semi-structured interviews with participants and focus groups with participants and trainers at the end of the intervention and at follow-up. Perceived impacts will be considered as complementary to results of the effectiveness evaluation assessed with standardised scales.[14]

For peer review only - http://bmjopen.bmj.com/site/about/guidelines.xhtml

Within the RRI framework, the SITLESS process evaluation explores if there are differing responses to the intervention by disadvantage categories related with gender, disability, ethnicity, and socio-economic position. It also considers how these categories affect implementation and mechanisms of impact and, consequently, the effectiveness of the intervention.[17]

For peer review only

Table 1. Specific qualitative procedures, pre-selected criteria for the purposeful sampling,

Qualitative procedure	Target	Pre-selected criteria for the purposeful sampling and numbers of each profile in each intervention site	Timeframe
Participant observation	Participants in the SMS group	*Criteria:*<br>1 from low socio-economic neighbourhood (or medium, if no low)<br>1 medium neighbourhood (or high, if no low)	During the SMS intervention
Semi-structured individual interviews	Participants in the SMS group	*Criteria:* (priority low and medium socio-economic status)<br>1 frail man & 1 frail woman<br>1 robust man & 1 robust woman<br>1 for each ethnic minority (if applicable)	End of intervention
		Criteria: 1 man & 1 woman (priority is low-to-medium socio-economic status)	12 months follow-up<br>18 months follow-up
	Participants of the ERS group	*Criteria:* 1 man & 1 woman (priority is low-to-medium socio-economic status)	12 months follow-up<br>18 months follow-up
	Participants of the control group	*Criteria:* (priority is low-to-medium socio-economic status)<br>1 man<br>1 woman	End of intervention<br>12 months follow-up<br>18 months follow-up
Focus groups	Participants of the SMS intervention groups	*Criteria:*<br>1 low socio-economic neighbourhood (or medium, if no low)<br>1 medium neighbourhood (or high, if no low)	End of intervention
	Participants of the ERS intervention groups	1 (priority is low-to-medium socio-economic status)	End of intervention
	ERS and SMS trainers	1 with all trainers involved in the ERS and SMS training.	End of intervention

For peer review only

For peer review only - http://bmjopen.bmj.com/site/about/guidelines.xhtml

Analysis plan

Data from the process evaluation will be analysed according to two main procedures: quantitative and qualitative analyses.

Quantitative analysis:

Quantitative process evaluation data, recorded in the attendance registry and the observation checklists, will be used to conduct a descriptive analysis, overall and by intervention site. The *SITLESS observation checklists* will be used to calculate the percentage of fidelity of the implementation regarding tasks and aims of the SMS and ERS sessions in each intervention site. The adherence to the ERS, SMS and control group sessions will be calculated as a percentage of sessions attended with each respective attendance registry. In addition, this registry will allow estimating the frequency of reasons for not attending. Percentage of fidelity of the implementation and adherence will be compared across intervention sites to identify whether differences may exist between them. Comparison will be conducted applying analysis of variance models.

Specific data will be entered in the main study database. This is the case for the variable fidelity and for adherence that will allow us to assess whether a better implemented intervention or a higher dose of the intervention is related with more effect, respectively. The potential mediator role of adherence/dose, the MSEQ, the PASR-12 and the LSNS on the effect of the intervention on physical activity and sedentary behaviour will be explored by covariance analyses, adding these scales as covariates to the mixed linear models testing the effect of the intervention. The statistical efficacy analysis of SITLESS has been described previously.[14] The information collected in the prespecified table on context characteristics will be used to describe each intervention site.

For peer review only - http://bmjopen.bmj.com/site/about/guidelines.xhtml

Qualitative analysis:

The SITLESS qualitative data analysis will be based on the transcripts of the semi-structured interviews and focus groups, and the trainers' notes from the participant observation. The Framework Method will be applied to analyse these data.[25] The Framework Method belongs to the thematic analysis or qualitative content analysis. These approaches first identify commonalities and differences in qualitative data, and then focus on relationships between different parts of the data to draw descriptive and/or explanatory conclusions clustered around themes. Accordingly, we will apply a combined approach to the analysis, enabling themes to be developed both inductively from the accounts (experiences and views) of research participants and deductively from existing literature. Regular team meetings will facilitate our critical exploration of participant responses, discussion of deviant cases and agreement on recurring themes.

The analysis will be conducted following these steps: transcription, familiarisation with the interview, coding of the transcripts and diaries of the participant observation, developing a working analytical framework, applying the analytical framework, charting data into the framework matrix and interpreting the data.

The initial coding of the first four transcripts, one per intervention site, will be conducted by two independent researchers, adding coding labels, notes and ideas. As a next step, a working analytical framework will be developed by discussing codes assigned, similarities and differences to achieve an agreement on a set of codes to establish an initial analytical framework. This framework will be refined by coding of further manuscripts until no new codes are generated by two independent researchers. Next, the final analytical framework will be applied to the remaining transcripts. The use of a software to conduct the analysis will be discussed. Once all the data are coded, we will summarize the data in a matrix for each theme.[25] The matrix will be comprised of one row per participant and one column per code with a separate sheet for each

For peer review only - http://bmjopen.bmj.com/site/about/guidelines.xhtml

category, using also verbatims into the corresponding cells. To interpret the data, themes are generated from the data set by reviewing the matrix and making connections within and between participants and categories. Lastly, analytical memos and team discussions will be used to develop themes aimed at offering possible explanations for what is happening within the data.

Triangulation with impact evaluation outcomes

As shown in Fig. 3, outcomes of the process evaluation are triangulated with impact evaluation outcomes (i.e., quantitative outcomes on effectiveness) to support their interpretation thus reinforcing each other. Specifically, quantitative outcomes on fidelity, adherence and from standardized scales are analysed as moderators with the impact evaluation outcomes. Descriptive results on context and qualitative findings on perceived effects are compared with impact results to complement them. Qualitative results and the overall quantitative results help to refine the logic model to finally support the interpretation of impact evaluation results.

Patient and public involvement

SITLESS, as a RRI project, has created guidance for the involvement of several different type of stakeholders in the project from the onset. They comprise older adults of both genders, representatives of older adults' associations, primary healthcare and sports professionals, policymakers and other local stakeholders of relevance (e.g., health insurance, where relevant). Accordingly, four local advisory boards were created at the beginning of the project, one on each intervention site (Barcelona, Odense, Belfast, Ulm), and were periodically involved in the study from its onset. The development of the research question and outcome measures were shared with each advisory board and therefore informed according to patients' priorities and motivations, experience and preferences. We also did a literature review that included how older adults perceive PA and SB, and how could we achieve sustained changes of behaviour to enhance health. The involvement of stakeholders as primary, secondary and tertiary end users

For peer review only - http://bmjopen.bmj.com/site/about/guidelines.xhtml

For peer review only

in the design of the study was specifically included in the intervention design. We explored experiences, preferences and priorities of older adults regarding behaviour change through focus groups that were convened with the assistance of the older people organisations belonging to the local advisory boards. We took into account their views at each site, and the main results were used to modify the intervention design. Local advisory boards also discussed and provided their contributions to the challenges faced regarding recruitment, retention of participants in the study and the dissemination strategies. Before the definitive trial, four feasibility studies were implemented. Qualitative interviews were conducted with participants in each pilot intervention site and from each arm of the trial to explore their perceptions and decide how to improve the intervention.

In the qualitative procedures undertaken along the main trial, patients are asked to assess the burden of the intervention and of their participation in the study.

At the end of the trial, we are planning on disseminating the results at each primary care centre and local leisure centres to end users, health professionals and relevant stakeholders. We also plan to share our results at relevant Citizen Science events, involving participants of each site where possible.

ETHICS AND DISSEMINATION

Ethics

The study design was approved by the Ethics and Research Committee of each intervention site: The Ethics and Research Committee of Fundació Blanquerna (Ramon Llull University, Spain), The Regional Committees on Health Research Ethics for Southern Denmark (University of Southern Denmark, Denmark), Office for Research Ethics Committees in Northern Ireland (ORECNI) (Queen’s University of Belfast) and the Ethical Review Board of Ulm University (Ulm, Germany). Participation is voluntary and all participants will be asked to sign informed consent before the start of the study.

For peer review only - http://bmjopen.bmj.com/site/about/guidelines.xhtml

Dissemination

The SITLESS dissemination plan operationalizes the main dissemination goal, which is to achieve a social impact by reaching academic and non-academic stakeholders, i.e., primary care and health care professionals, older adults, policy makers, professionals related to physical activity promotion, etc. Specifically, process evaluation results will enhance social impact by providing implementation details that might be of interest for policy makers. In addition, qualitative results (e.g., quotations of participants) will support reaching non-academic stakeholders. A Data Management Plan has been developed for the SITLESS study describing each specific dataset in the study and regulating its deposition and curation.

All publications from the SITLESS project, including those related with process evaluation, will be open access.

Conclusions

The SITLESS project has developed a process evaluation to understand how implementation, causal mechanisms and the contextual factors shape the outcomes of a complex intervention that enhances ERS with SMS to reduce sedentary behaviour and increase physical activity among community dwelling older adults of four European countries. Accordingly, this trial has the purpose of facilitating future intervention development by informing policy-makers, practitioners and researchers about implementation, mechanisms of impact and contextual factors in detail. In case the intervention is proven as being effective and cost-effective, the information will be focused on the actually delivered intervention to allow replication in new contexts considering the relevant core components, context characteristics and implementation challenges. In case of being ineffective, process evaluation results will contribute to define whether the failure can be attributed to the intervention itself or to a poor implementation. Hence, process evaluation will provide useful data for systematic reviews to compare interventions considering their implementation and contextual characteristics.

For peer review only - http://bmjopen.bmj.com/site/about/guidelines.xhtml

Trial status

This initial phase of this project commenced in May 2015. A feasibility study was performed from December 2015 until June 2016. The recruitment for the trial started in July 2016. At the time of submission of this protocol, all 1390 participants have been included in the study. To date, follow-up assessments are being conducted and are planned to finish by April 2019.

Author Contributions:

LCP, MT, MGG, PC, EM and DR contributed to the conception of the study. LCP, SB, KW, MS and NB contributed to the development of the specific procedures.

LCP will coordinate the overall development of the process evaluation. LCP, KW, MS and NB will coordinate the process evaluation in their respective intervention sites and LCP, SB, KW, MS and NB will contribute to the acquisition of process evaluation data.

LCP, SB, MT, PC, MGG, CM, FK, MSk, KW, NB, MDeid, EM, DR, RGR, JJ, OS, MSa; JW, MG, DG, MDenk, AS and MR contributed to the design of the final process evaluation and will contribute to the analysis and interpretation of data and writing of the manuscript. LCP and MR contributed to the data management plan and analysis.

All authors provided feedback on the drafts of this paper and have read and approved the final manuscript.

Acknowledgements

We would like to thank the advisory board members from Barcelona, Odense, Ulm and Belfast and the participants of the feasibility study and the trial for their valuable contributions.

Data statement: Not applicable.

Funding:

For peer review only

This work was supported by the European Union program Horizon 2020 with the grant number H2020-Grant 634270.

Competing interests:

The authors declare that they have no competing interests.

Ethics approval:

The study design was approved by the Ethics and Research Committee of each intervention site: The Ethics and Research Committee of Ramon Llull University (reference number: 1314001P) (Fundació Blanquerna, Spain), The Regional Committees on Health Research Ethics for Southern Denmark (reference number: S-20150186) (University of Southern Denmark, Denmark), Office for Research Ethics Committees Northern Ireland (ORECNI reference number: 16/NI/0185) (Queen's University of Belfast) and the Ethical Review Board of Ulm University (reference number: 354/15) (Ulm, Germany). Participation is voluntary and all participants will be asked to give written informed consent before the start of the study.

Open access

Not applicable.

For peer review only - http://bmjopen.bmj.com/site/about/guidelines.xhtml

REFERENCES

1. Berwick, D. M. The science of improvement. *JAMA - Journal of the American Medical Association* **299,** 1182–1184 (2008).

2. Campbell, N. C. *et al.* Designing and evaluating complex interventions to improve health care. *BMJ* **334,** 455–9 (2007).

3. Campbell, N. C. *et al.* Designing and evaluating complex interventions to improve health care. *BMJ* **334,** 455–9 (2007).

4. Moore, G., Audrey, S., Barker, M. & Bond, L. Process evaluation of complex interventions. *UK Med. Res. Counc. Guid. Prep.* 19-45; 64-75 (2014). doi:http://www.populationhealthsciences.org/MRC-PHSRN-Process-evaluation-guidance-final-2-.pdf

5. Moore, G. F. *et al.* Process evaluation of complex interventions: Medical Research Council guidance. *BMJ Br. Med. J.* **350,** 1–7 (2015).

6. Pfadenhauer, L. M. *et al.* Making sense of complexity in context and implementation: The Context and Implementation of Complex Interventions (CICI) framework. *Implement. Sci.* **12,** 1–17 (2017).

7. Lee, I. M. *et al.* Effect of physical inactivity on major non-communicable diseases worldwide: An analysis of burden of disease and life expectancy. *Lancet* **380,** 219–229 (2012).

8. Who, W. H. O. Global recommendations on physical activity for health. *Geneva World Heal. Organ.* 60 (2010). doi:10.1080/11026480410034349

9. Ding, D. *et al.* The economic burden of physical inactivity: a global analysis of major non-communicable diseases. *Lancet* **388,** 1311–1324 (2016).

10. Owen, N., Sparling, P. B., Healy, G. N., Dunstan, D. W. & Matthews, C. E. Sedentary behavior: Emerging evidence for a new health risk. *Mayo Clinic Proceedings* **85,** 1138–1141 (2010).

11. Koster, A. *et al.* Association of Sedentary time with mortality independent of moderate to vigorous physical activity. *PLoS One* **7,** (2012).

12. Harvey, J. A., Chastin, S. F. M. & Skelton, D. A. Prevalence of sedentary behavior in older adults: a systematic review. *Int. J. Environ. Res. Public Health* **10,** 6645–61 (2013).

13. Ekelund, U. *et al.* Does physical activity attenuate, or even eliminate, the detrimental association of sitting time with mortality? A harmonised meta-analysis of data from more than 1 million men and women. *Lancet* **388,** 1302–1310 (2016).

14. Giné-Garriga, M. *et al.* The SITLESS project: Exercise referral schemes enhanced by self-management strategies to battle sedentary behaviour in older adults: Study protocol for a randomised controlled trial. *Trials* **18,** (2017).

15. Deidda, M. & Coll-Planas, L. Cost effectiveness of exercise referral schemes enhanced by self-management strategies to battle sedentary behaviour in older adults: protocol

For peer review only - http://bmjopen.bmj.com/site/about/guidelines.xhtml

for an economic evaluation alongside the SITLESS three-armed pragmatic randomised clinical trial. *BMJ Open* **8,** (2018).

16. Lewin, S. *et al.* Assessing the complexity of interventions within systematic reviews: Development, content and use of a new tool (iCAT-SR). *BMC Med. Res. Methodol.* **17,** 1–13 (2017).

17. Bardone, E. & Lind, M. Towards a phronetic space for responsible research (and innovation). *Life Sci. Soc. Policy* **12,** 5 (2016).

18. Marcus, B. & Forsyth, L. *Motivating people to be physically active*. (Human Kinetics, 2009).

19. Marcus, B. H., Selby, V. C., Nlaura, R. S. & Rossi, J. S. Self-efficacy and the stages of exercise behavior change. *Res. Q. Exerc. Sport* **63,** 60–66 (1992).

20. Umstattd, M. R., Motl, R., Wilcox, S., Saunders, R. & Watford, M. Measuring physical activity self-regulation strategies in older adults. *J. Phys. Act. Health* **6 Suppl 1,** S105-12 (2009).

21. Lubben, J. E. Assessing social networks among elderly populations. *Fam. Community Health* **11,** 42–52 (1988).

22. Garratt, A. M., Løchting, I., Smedslund, G. & Hagen, K. B. Measurement properties of instruments assessing self-efficacy in patients with rheumatic diseases. *Rheumatology* **53,** 1161–1171 (2014).

23. Mendoza-Vasconez, A. S., Marquez, B., Benitez, T. J. & Marcus, B. H. Psychometrics of the self-efficacy for physical activity scale among a Latina women sample. *BMC Public Health* **18,** 1097 (2018).

24. Lubben, J. *et al.* Performance of an abbreviated version of the Lubben Social Network Scale among three European community-dwelling older adult populations. *Gerontologist* **46,** 503–13 (2006).

25. Gale, N. K., Heath, G., Cameron, E., Rashid, S. & Redwood, S. Using the framework method for the analysis of qualitative data in multi-disciplinary health research. *BMC Med. Res. Methodol.* **13,** 117 (2013).

Figure's legends:

Figure 1. The logic model of the SITLESS intervention.

Figure 2. The process evaluation framework for the SITLESS trial.

**** IDEA:** refers to the IDEA problem solving framework. [18]

Figure 3. Specific aims, procedures and outcomes of the process evaluation and their triangulation with impact evaluation outcomes

For peer review only - http://bmjopen.bmj.com/site/about/guidelines.xhtml

APPENDIX

Appendix 1: SEMI-STRUCTURED QUESTIONNAIRE FOR INTERVIEWS WITH PARTICIPANTS OF THE SMS GROUP

Appendix 2: SEMI-STRUCTURED QUESTIONNAIRE FOR THE FOCUS GROUP FOR THE SMS INTERVENTION GROUPS

Appendix 3: OBSERVATION CHECKLIST OF THE SMS INTERVENTION SESSIONS

Appendix 4: OBSERVATION CHECKLIST OF THE PA SESSIONS

Appendix 5: ATTENDANCE LIST FOR THE SMS SESSIONS

Appendix 6: ATTENDANCE LIST FOR THE PA SESSIONS

For peer review only

For peer review only - http://bmjopen.bmj.com/site/about/guidelines.xhtml

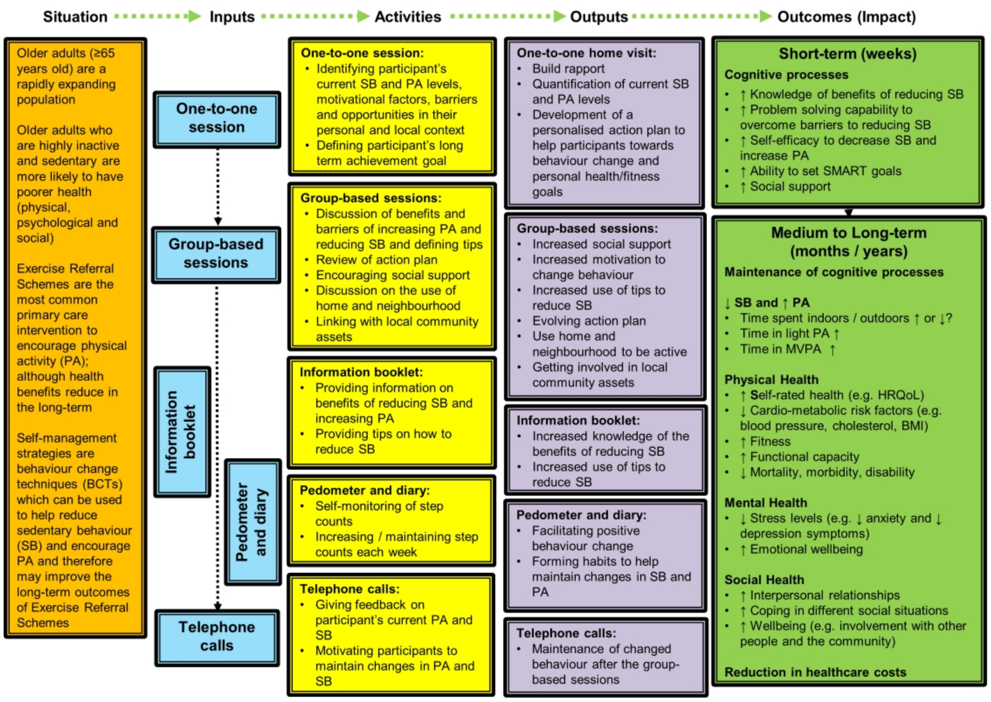

254x190mm (300 x 300 DPI)

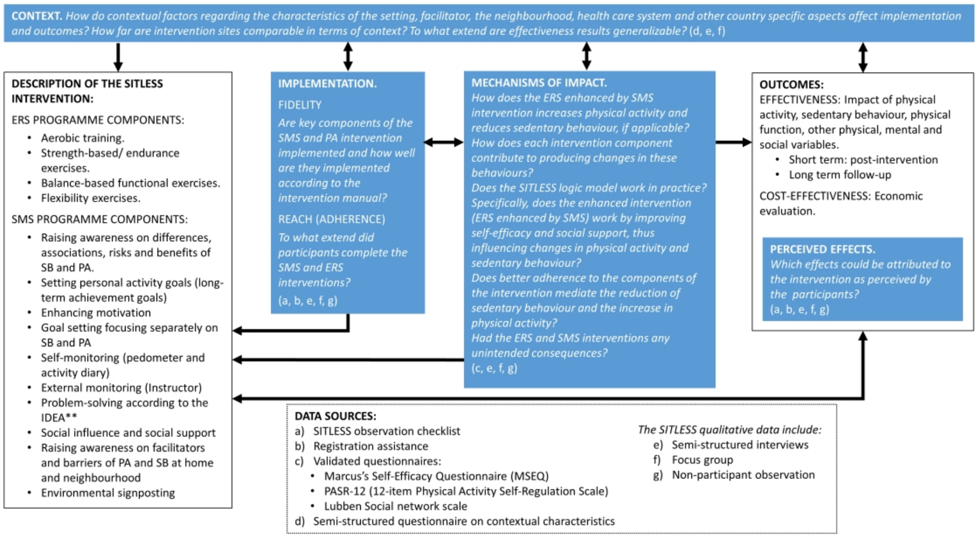

338x190mm (300 x 300 DPI)

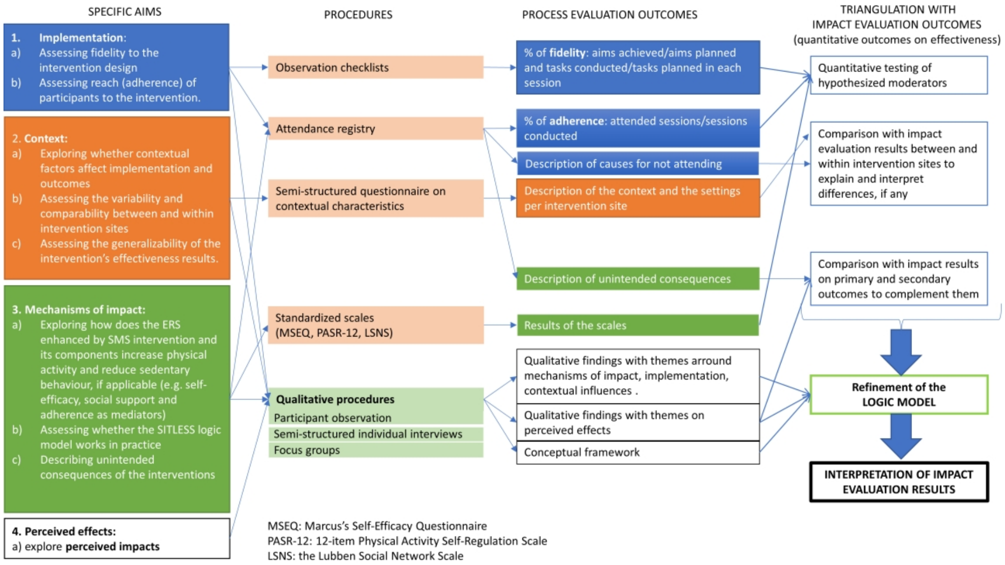

338x190mm (300 x 300 DPI)

Appendix 1: SEMI-STRUCTURED QUESTIONNAIRE FOR INTERVIEWS WITH PARTICIPANTS OF THE SMS GROUP

Note: Aspects related with gender, frailty/robustness and ethnicity have been considered as elegibility criteria and will be considered in the content analysis. However, this questionnaire does not include any specific question regarding these aspects and are considered to emerge in their discourse.

Part one: introduction (5')

Section	Comments
**Introduction**	• Acknowledgements<br>• The conductor introduces briefly him/her self to the participant<br>• Inform about the duration and the aims of the meeting<br>• The conductor informs the participant that the meeting will be audio-recorded for later analysis.<br>• The conductor reminds the participant to turn-off or silence the phone.
**Informed consent**	• The conductor asks for permission about recording of the interview and informed consent.<br>• Reading and signing of informed consent.

For peer review only

Part two: overall mechanisms of impact (20’)

Dimension	Questions
According to participant’ perceptions: how do the participant’ actively respond and interact with the ***overall intervention****?*<br><br>**<u>Note:</u>** take into account that the SMS intervention requires following tasks:<br>○ *Fill the diary daily: steps or PA time, and calculate means.*<br>○ *Wear daily, open and read the pedometer.*<br>○ *Set goals on PA and follow-up accomplishment.*<br>○ *Set goals on SB and follow-up accomplishment (SITLESS tips).*<br>○ *Active participation in group discussions.*<br>○ *Telephone prompts*	• What are your general impression / thoughts of the intervention?<br>• Overall how satisfied are you with the intervention?<br>• What specific features of the intervention impressed you?<br>• What features of the intervention disappointed you?<br>• How likely would you be to recommend this intervention to a family member or friend? Why?<br>• What, if anything, do you find frustrating or unappealing about the intervention?<br>• Do you have any suggestions on how we could improve the intervention to make it more appealing?<br>• Were any of tasks required in the intervention difficult for you to perform?

For peer review only

Part three: overall perceived effects and mechanisms of impact (20')

Dimension	Questions
**Secondary outcomes**	• Have you perceived any effects of the intervention? Which ones?<br>• Have you perceived any specific effects on<br>○ General health?<br>○ Mental health? In terms of depression, loneliness, etc. and psychological/emotional wellbeing?<br>○ Physical health?<br>○ Social relationships?
**Primary outcomes: Effects on PA and SB**	
How does the SMS intervention produce change?	• During the intervention: have you increased your PA? (besides the ERS sessions)<br>• In case that you have increased your PA, why do you think you have done it?, Because your physical capacity increased<br>○ Because your self-efficacy increased<br>○ For other reasons<br>• In case that you have not increased your PA, why do you think you haven't done it?<br>• During the program: have you decreased your SB?<br>• If so, in which manner?<br>○ Incorporate new routines<br>○ Other<br>• If not, why do you think that?
**Mid-long term effects on PA and SB**	• Do you think you will maintain your increased PA / decreased SB once the intervention has finished?<br>• If so, in which manner?<br>• If not, why do you think that?

For peer review only

Part four: mechanisms of impact for each component (20’)

Dimension	Questions
• *How do **each intervention components** contribute to produce change?*<br>• *Specifically, does the intervention work by improving self-efficacy and social support?*<br>• *Does a better accomplishment of the goal setting, self-monitoring and cues mediate the reduction of sedentary behaviour and the increase in physical activity?”*	• Can you state the strategies you have used to accomplish each session’s goals? Have you accomplished them by yourself?<br>• What are the reasons why you have decided to accomplish (or not) the goals?<br>• How and why each component supported/permitted to achieve the goals of the intervention? (mechanisms of impact)<br>○ Raising awareness on differences, associations, risks and benefits of SB and PA.<br>○ Setting personal activity goals (long-term achievement goals)<br>○ Goal setting focusing separately on PA<br>○ Goal setting focusing separately on SB (SITLESS tips)<br>○ Self-monitoring:<br>▪ pedometer<br>▪ activity diary<br>○ External monitoring (Instructor)<br>○ Problem-solving<br>○ Raising awareness on facilitators and barriers of PA and SB at home and at the neighborhood (environmental signposting)<br>○ Peer and social support from the group<br>○ The trainer<br>○ Telephone prompts

For peer review only

Part five: context (20')

Dimension	Questions
**Physical environment**<br><br>*How do contextual factors regarding the characteristics of the neighbourhood affect outcomes according to participants' perceptions?*	• How do you perceive the physical environment of your neighbourhood?<br>• How do you perceive the specific settings of the intervention? (i.e., facilities used for the SMS and PA sessions)<br>○ Primary health care centre<br>○ Parks<br>○ Sports facilities<br>○ Community resources<br>• How do you perceive these specific aspects of your neighbourhood? (especially considering whether and how these aspects affect doing PA in the neighbourhood)<br>○ Socio-economic level<br>○ Urban/rural setting<br>○ Orography / walkability<br>○ Pleasant environment (comfortable, traffic…)<br>○ Personal safety (crimes, robberies, …)<br>○ Availability of places within an easy walking distance to do informal PA (parks, urban gym, …) and to do formal PA (organized PA offers)
**Personal networks**<br><br>*How do contextual factors regarding the characteristics of the personal network affect outcomes according to the participant's perceptions?*	• Do you think your personal situation or health status (e.g., having a supportive social network, being a caregiver, living alone, having a chronic disease, etc.) helps or hinders your physical activity and your sedentary behavior? How and why?<br>• Do you think your personal network (partners, family, friends) helps or hinders your physical activity and your sedentary behavior? How and why?<br>• Did you know other participants of the group before? How has that affected your participation in the group?

For peer review only

Appendix 2: SEMI-STRUCTURED QUESTIONNAIRE FOR THE FOCUS GROUP FOR THE SMS INTERVENTION GROUPS

Part one: introduction and presentation of participants (10')

Section	Comments
**Introduction**	• Acknowledgements<br>• The conductor introduces briefly him/her self to the participants<br>• Inform about the duration and the aims of the meeting<br>• The conductor informs the participants that the meeting will be audio-recorded for later analysis.<br>• The conductor reminds the participants to turn-off or silence phones.
**Informed consent**	• The conductor asks for permission about recording of the meeting and informed consent.<br>• Reading and signing of informed consent.
**Presentation of participants**	• Kindly ask participants to introduce their selves to the rest of the group.

For peer review only

Part two: overall mechanisms of impact (20')

Dimension	Questions
According to participants' perceptions: how do participants' actively respond and interact with the ***overall intervention****?*<br><br>**Note:** take into account that the SMS intervention requires following tasks:<br>○ *Fill the diary daily: steps or PA time, and calculate means.*<br>○ *Wear daily, open and read the pedometer.*<br>○ *Set goals on PA and follow-up accomplishment.*<br>○ *Set goals on SB and follow-up accomplishment (SITLESS tips).*<br>○ *Active participation in group discussions.*<br>○ *Telephone promots*	• What are your general impression / thoughts of the intervention?<br>• Overall how satisfied are you with the intervention?<br>• What specific features of the intervention impressed you?<br>• What features of the intervention disappointed you?<br>• How likely would you be to recommend this intervention to a family member or friend? Why?<br>• What, if anything, do you find frustrating or unappealing about the intervention?<br>• Do you have any suggestions on how we could improve the intervention to make it more appealing?<br>• Were any of tasks required in the intervention difficult for you to perform?

For peer review only

Part three: overall perceived effects and mechanisms of impact (20')

Dimension	Questions
**Secondary outcomes**	• Have you perceived any effects of the intervention? Which ones?<br>• Have you perceived any specific effects on<br>○ General health?<br>○<br>○ Mental health? In terms of depression, loneliness, etc. and psychological/emotional wellbeing?<br>○ Physical health?<br>○ Social relationships?
**Primary outcomes: Effects on PA and SB**<br><br>*How does the SMS intervention produce change?*	• During the intervention: have you increased your PA? (besides the ERS sessions)<br>• In case that you have increased your PA, why do you think you have done it?, Because your physical capacity increased<br>○ Because your self-efficacy increased<br>○ For other reasons<br>• In case that you have not increased your PA, why do you think you haven't done it?<br>• During the program: have you decreased your SB?<br>• If so, in which manner?<br>○ Incorporate new routines<br>○ Other<br>• If not, why do you think that?
**Mid-long term effects on PA and SB**	• Do you think you will maintain your increased PA / decreased SB once the intervention has finished?<br>• If so, in which manner?<br>• If not, why do you think that?

Part four: mechanisms of impact for each component (20')

For peer review only

Dimension	Questions
▸ *How do **each intervention components** contribute to produce change?*<br><br>▸ *Specifically, does the intervention work by improving self-efficacy and social support?*<br><br>▸ *Does a better accomplishment of the goal setting, self-monitoring and cues mediate the reduction of sedentary behaviour and the increase in physical activity?"*	• Can you state the strategies you have used to accomplish each session's goals? Have you accomplished them by yourself?<br>• What are the reasons why you have decided to accomplish (or not) the goals?<br>• How and why each component supported/permitted to achieve the goals of the intervention? (mechanisms of impact)<br>○ Raising awareness on differences, associations, risks and benefits of SB and PA.<br>○ Setting personal activity goals (long-term achievement goals)<br>○ Goal setting focusing separately on PA<br>○ Goal setting focusing separately on SB (SITLESS tips)<br>○ Self-monitoring:<br>▪ pedometer<br>▪ activity diary<br>○ External monitoring (Instructor)<br>○ Problem-solving<br>○ Raising awareness on facilitators and barriers of PA and SB at home and at the neighborhood (environmental signposting)<br>○ Peer and social support from the group<br>○ The trainer<br>○ Telephone prompts

For peer review only

Part five: context (20')

Dimension	Questions
**Physical environment**<br><br>*How do contextual factors regarding the characteristics of the neighbourhood affect outcomes according to participants' perceptions?*	• How do you perceive the physical environment of your neighbourhood?<br>• How do you perceive the specific settings of the intervention? (i.e., facilities used for the SMS and PA sessions)<br>○ Primary health care centre<br>○ Parks<br>○ Sports facilities<br>○ Community resources<br>• How do you perceive these specific aspects of your neighbourhood? (especially considering whether and how these aspects affect doing PA in the neighbourhood)<br>○ Socio-economic level<br>○ Urban/rural setting<br>○ Orography / walkability<br>○ Pleasant environment (comfortable, traffic…)<br>○ Personal safety (crimes, robberies, …)<br>○ Availability of places within an easy walking distance to do informal PA (parks, urban gym, …) and to do formal PA (organized PA offers)
**Personal networks**<br><br>*How do contextual factors regarding the characteristics of the personal network affect outcomes according to participants' perceptions?*	• Do you think your personal situation or health status (e.g., having a supportive social network, being a caregiver, living alone, having a chronic disease, etc.) helps or hinders your physical activity and your sedentary behavior? How and why?<br>• Do you think your personal network (partners, family, friends) helps or hinders your physical activity and your sedentary behavior? How and why?<br>• Did you know other participants of the group before? How has that affected your participation in the group?

For peer review only

Appendix 3: OBSERVATION CHECKLIST OF THE SMS INTERVENTION SESSIONS

Centre: Group: Trainer:

Date: Session number: 2	
Week: 3	Stage: Ramping

FIRST GROUP-BASED SESSION Length of session: ______ minutes

Tasks	Completed	Partially completed	Not completed
**Activity 1** - Identifying the benefits of being less sedentary and more active.			
**Activity 2** - Share and discuss personal lifestyles related to sedentary behaviour, physical activity and physical exercise.			
**Activity 3** - Share and discuss tips for sitting less			
**Activity 4** - Retrieve the participants' baseline daily steps and/or weekly time in physical activities / exercise and discuss the reasons for differences between certain days.			
**Activity 5** - Agree on which SITLESS tips the participants will try over the next week.			
**FINAL TASKS**<br>• Make a statement to encourage the group to practise the SITLESS tips:<br>• The final tasks are to arrange a date/time for the next session and to remind the participants to bring their information booklets and activity diaries			

Aims of the session	Completed	Partially completed	Not completed
1. To identify the benefits of being less sedentary and more active.			
2. To share and discuss personal lifestyles related to sedentary behaviour, physical activity and physical exercise			
3. To share and discuss tips for sitting less.			
4. To retrieve the participants' baseline daily steps and/or weekly time in physical activities / exercise and discuss the reasons for differences between certain days.			
5. To agree on which SITLESS tips the participants will try over the next week.			

For peer review only

Centre: Group: Trainer:

Date: Session number:
Week: Stage:

Which tasks went particularly well during the session? Why?

Were any tasks difficult to implement? Why?

Were any modifications made to the planned session? Why? *(adhoc tailoring)*

Have you observed any adverse effects? Which ones?

Generally, what was the participant's response / attitude to the different activities (e.g. uninterested, excitable, attentive, sceptical, quiet, chatty)? Have you observed specific responses to be highlighted regarding gender, educational level, ethnicity..? Which ones?

Other comments

For peer review only

Appendix 4: OBSERVATION CHECKLIST OF THE PA SESSIONS

Note: This annex contains the standard Observation Checklist of the PA sessions with all the information needed from each intervention site. This checklist has been adapted by each intervention site in order to avoid repetition by keeping track only of aspects of the PA interventions that may vary from session to session. E.g., Odense is conducting all sessions indoors and trainers do not need to register this aspect after each session.

Centre: Group: Trainer:

Date: Session number:

This checklist should be filled in by the PA instructor after EACH physical activity session

DATA ON EACH SESSION REPORTED BY THE INSTRUCTOR

1. Where have you conducted the physical activity session?

Indoors/outdoors Specific setting:___________

2. Has the climate / environment affected this session? *(for an individual / overall group)* YES/NO

How? _________________________________

3. Was this session conducted by the usual instructor? YES/NO
4. Have you tailored the physical activity session to the frail participants? YES/NO

How?____

5. Have you personalized the progression in the exercise intensity? YES/NO

How?____

6. Have you tailored the physical activity session to specific participants for other reasons? YES/NO

How? ____

7. Use of equipment/ material:

Indicate the use of weight lifting equipment	Yes No
Indicate the use of cardiovascular machines	Yes No
Indicate the use of other material (e.g. therabands, balls, bosu)	Yes No<br>If yes, which:_______<br>_________________
Indicate the use of recycled/ adapted material	Yes No
Indicate the use of music	Yes No

For peer review only

8. Have you applied any group dynamics to promote group cohesion? (e.g., cooperative games, work in pairs...)

How? ____

9. Every second session, use the final 5 minutes of session to ask the participants whether they have done the extra Physical Activity session on their own per week (defined as at least 30 minutes of moderate or vigorous PA following the WHO guidelines, for instance brisk walking)

Number of participants that had done the extra Physical Activity session/ out of the total attending → calculate percentage:

………………../………………: ____%

10. Observations relevant for the study (e.g. gender specific issues?)

Please add here observations on the development of the session. Add the participant code to refer to observations of specific participants

……

……

……

……

……

……

For peer review only

Appendix 5: ATTENDANCE LIST FOR THE SMS SESSIONS

Centre: Group: Trainer:

		Dates of the sessions (dd/mm/yy)										
**Participant code**	**Participant name**	One-to-one	1st group session	2nd group session	3rd group session	4th group session	5th group session	6th group session	1st phone call	2nd phone call	3rd phone call	4th phone call
**EXAMPLE:** CL004	Maria Roca	√ 01.02.16	√	¥ B	√	√	√	√	√ 5/05/16	√ 6/07/16	∏ E 01-15/10/16	∏ E 01-15/01/17

√ = Attendance. In case a certain participant arrived considerably late or performed only part of the session (e.g., arrived 20 minutes late or did half of the exercises due to pain), report the % performed of the session.

¥ = Unexcused absence (e.g.., the trainer was NOT previously informed)

∏ = Excused absence (e.g.., the trainer was previously informed)

For peer review only

Appendix 5: ATTENDANCE LIST FOR THE SMS SESSIONS

Centre: Group: Trainer:

Please, state the reason for non-attendance: (A) health problem (cold, illness, surgery, etc), (B) medical visit, (C) trip/travel, (D) familiar reason (taking care of grandsons, etc), (E) other (specify).

Please, state any **adverse effect** for participant and session (e. g. anxiety).

Please, fill the table in case you need to specify the reason for non-attendance or the adverse effect. Include those objective or perceived adverse effects that happened during this SMS session, that participants and/or the trainer attribute to the SMS intervention.

Participant code	Date	Note of reasons for non-attendance	Note on adverse effect
CL004	01-15/10/16	Participant not reached after repeated calls	
CL004	01-15/01/17	Participant not reached after repeated calls	

For peer review only

Appendix 6: ATTENDANCE LIST FOR THE PA SESSIONS

Centre: Group: Trainer:

			Dates of the sessions (dd/mm/yy)															
**Participant code**	**Participant name**	**SPPB**	01.02.16	03.02.16	08.02.16	10.02.16	15.02.06	17.02.16										
**EXAMPLE:** CL004	Maria Roca	L	√	X	∏ B	√	√ 2 50%	∏ E										

√ = Attendance. In case a certain participant arrived considerably late or performed only part of the session (e.g., arrived 20 minutes late or did half of the exercises due to pain), report the % performed of the session.

X = Unexcused absence (i.e., the trainer was NOT previously informed)
∏ = Excused absence (i.e., the trainer was previously informed)
Please, state the **SPPB** (Short Physical Performance Battery) as L (low), M (medium), or H (high)

Please, state any **adverse effect** of physical activity (PA) for participant and session, according to following codes: (1) stiffness after PA, (2) joint pain, (3) fall during PA session, (4) dizziness, (5) other (specify)

For peer review only

Please, state the **reason for non-attendance**: (A) health problem (cold, illness, surgery, etc.), (B) medical visit, (C) trip/travel, (D) familiar reason (taking care of grandsons, etc.), (E) other (specify)

Please, fill the table in case you need to specify the reason for non-attendance or the adverse effect. Include those objective or perceived adverse effects that participants and/or the trainer attribute to the PA session.

**Participant code**	**Date**	**Note of reasons for non-attendance**	**Note on adverse effect**
CL004	17.02.16	Forgot the session	

For peer review only

BMJ Open

Exercise referral schemes enhanced by self-management strategies to reduce sedentary behaviour and increase physical activity among community-dwelling older adults from four European countries: protocol for the process evaluation of The SITLESS randomised controlled trial

Journal:	*BMJ Open*
Manuscript ID	bmjopen-2018-027073.R2
Article Type:	Protocol
Date Submitted by the Author:	23-Apr-2019
Complete List of Authors:	Coll-Planas, Laura; Universitat Autònoma de Barcelona, Fundació Salut i Envelliment (Foundation on Health and Ageing)- UAB; Institute of Biomedical Research (IIB Sant Pau)<br>Blancafort Alias, Sergi; Universitat Autònoma de Barcelona, Fundació Salut i Envelliment (Foundation on Health and Ageing)- UAB; Institute of Biomedical Research (IIB Sant Pau)<br>Tully, Mark; Ulster University, Institute of Mental Health Sciences, School of Health Sciences; Queen's University Belfast, 2. UKCRC Centre of Excellence for Public Health Research (Northern Ireland)<br>Caserotti, Paolo; Syddansk Universitet, Department of Sports Science and Clinical Biomechanics, Center for Active and Healthy Ageing (CAHA)<br>Giné-Garriga, Maria; Universitat Ramon Llull, Facultat de Psicologia, Ciències de l'Educació i de l'Esport Blanquerna; Glasgow Caledonian University, School of Health and Life Sciences<br>Blackburn, Nicole; Queen's University Belfast School of Medicine Dentistry and Biomedical Sciences, UKCRC Centre of Excellence for Public Health (NI), Centre for Public Health<br>Skjødt, Mathias; Syddansk Universitet , Department of Sports Science and Clinical Biomechanics, Center for Active and Healthy Ageing (CAHA)<br>Wirth, Katharina ; Universitat Ulm, Institute of Epidemiology and Medical Biometry; Agaplesion Bethesda Clinic, Geriatric Research Unit Ulm University and Geriatric Center Ulm Alb-Donau,<br>Deidda, Manuela; University of Glasgow, Health Economics and Health Technology assessment<br>McIntosh, Emma; University of Glasgow Institute of Health and Wellbeing, Health Economics and Health Technology Assessment (HEHTA)<br>Rothenbacher, Dietrich; Ulm University, Institute of Epidemiology and Medical Biometry<br>Gallardo Rodríguez, Rodrigo; Universidad Catolica de la Santisima Concepcion, Departamento de Ciencias del Deporte y Acondicionamiento Físico, CEADE<br>Jerez-Roig, J.; Universitat de Vic - Universitat Central de Catalunya, 2. Research group on Methodology, Methods, Models and Outcomes of Health and Social Sciences (M3O). Faculty of Health Science and Welfare. Centre for Health and Social Care Research (CESS). ; Universitat Ramon Llull, 1. Facultat de Psicologia, Ciències de l'Educació i

	de l’Esport Blanquerna<br>Sansano-Nadal, Oriol; Universitat Ramon Llull, Facultat de Psicologia, Ciències de l’Educació i de l’Esport Blanquerna<br>Santiago, Marta; Universitat Ramon Llull, Facultat de Psicologia, Ciències de l’Educació i de l’Esport Blanquerna<br>Wilson, Jason; Queen's University Belfast School of Medicine Dentistry and Biomedical Sciences, Centre for Public Health<br>Guerra-Balic, Míriam ; Universitat Ramon Llull, Facultat de Psicologia, Ciències de l’Educació i de l’Esport Blanquerna<br>Martín-Borràs, Carme; Faculty of Psychology, Education and Sport Sciences (FPCEE) Blanquerna, Ramon Llull University., Department of Physical Activity and Sport Sciences; Faculty of Health Sciences (FCS) Blanquerna, Ramon Llull Univesity., Department of Physical Therapy<br>Gonzalez, Denise; Sport Initiative et Loisir Bleu Association; Sport Initiative et Loisir Bleu Association<br>Lefebvre, Guillaume; Sport Initiative et Loisir Bleu Association; Sport Initiative et Loisir Bleu Association<br>Denkinger, Michael; Universitat Ulm, Agaplesion Bethesda Clinic, Geriatric Research Unit Ulm University and Geriatric Center Ulm Alb-Donau<br>Kee, Frank; Queen's University Belfast, UKCRC Centre of Excellence for Public Health (NI)<br>Salvà Casanovas, Antoni ; Universitat autonoma de Barcelona, Fundació Salut i Envelliment (Foundation on Health and Ageing)- UAB; Institute of Biomedical Research (IIB Sant Pau)<br>Roqué i Figuls , Marta; Universitat Autònoma de Barcelona, Fundació Salut i Envelliment (Foundation on Health and Ageing)- UAB; Institute of Biomedical Research (IIB Sant Pau)
**Primary Subject Heading**:	Public health
Secondary Subject Heading:	Research methods, Epidemiology
Keywords:	EPIDEMIOLOGY, PUBLIC HEALTH, GERIATRIC MEDICINE

SCHOLARONE™ Manuscripts

TITLE PAGE:

- **Title:**

Exercise referral schemes enhanced by self-management strategies to reduce sedentary behaviour and increase physical activity among community-dwelling older adults from four European countries: protocol for the process evaluation of The SITLESS randomised controlled trial

Authors: **Coll-Planas, Laura; Blancafort Alias, Sergi; Tully, Mark A; Caserotti, Paolo; Giné-Garriga, Maria; Blackburn, Nicole E; Skjødt, Mathias; Wirth, Katharina; Deidda, Manuela; McIntosh, Emma; Rothenbacher, Dietrich; Gallardo Rodríguez, Rodrigo; Jerez-Roig, Javier; Sansano-Nadal, Oriol; Santiago, Marta; Wilson, Jason J; Guerra-Balic, Miriam; Martín-Borràs, Carme; González, Denise; Lefebvre, Guillaume; Denkinger, Michael; Kee, Frank; Salvà Casanovas, Antoni; Roqué i Figuls, Marta.**

- **Corresponding author:**

Full name: Laura Coll i Planas
Postal address: Fundació Salut i Envelliment (Foundation on Health and Ageing)- Universitat Autònoma de Barcelona
Sant Antoni Maria Claret, 171 E-08041
Barcelona
E-mail: laura.coll@uab.cat

Tel.: +34 93 446 45 06

- **Co-authors:**

Coll-Planas, Laura
Blancafort Alias, Sergi
Salvà Casanovas, Antoni
Roqué i Figuls, Marta.

1. Fundació Salut i Envelliment (Foundation on Health and Ageing)- Universitat Autònoma de Barcelona
   Barcelona, Spain
2. Institute of Biomedical Research (IIB Sant Pau), Barcelona, Spain

Tully, Mark A

1. Institute of Mental Health Sciences, School of Health Sciences, Ulster University, Newtownabbey, Northern Ireland, UK.
2. UKCRC Centre of Excellence for Public Health Research (Northern Ireland), Queen's University Belfast, Belfast, Northern Ireland, UK.

Blackburn, Nicole E
Wilson, Jason J;

For peer review only - http://bmjopen.bmj.com/site/about/guidelines.xhtml

Kee, Frank
UKCRC Centre of Excellence for Public Health (NI), Centre for Public Health, School of Medicine, Dentistry and Biomedical Sciences, Queen's University Belfast, Belfast, UK.

Caserotti, Paolo
Skjodt, Mathias
Department of Sports Science and Clinical Biomechanics, Center for Active and Healthy Ageing (CAHA), University of Southern Denmark, Odense, Campusvej 55, 5230 Odense M, Denmark

Giné-Garriga, Maria
1. Facultat de Psicologia, Ciències de l'Educació i de l'Esport Blanquerna, Universitat Ramon Llull, C/ Císter 34, 08022 Barcelona, Spain
2. School of Health and Life Sciences, Glasgow Caledonian University, Cowcaddens Road, Glasgow, United Kingdom

Sansano-Nadal, Oriol
Santiago, Marta
Guerra, Míriam
Martín-Borràs, Carme
Facultat de Psicologia, Ciències de l'Educació i de l'Esport Blanquerna, Universitat Ramon Llull, C/ Císter 34, 08022 Barcelona, Spain

Jerez-Roig, Javier
1. Facultat de Psicologia, Ciències de l'Educació i de l'Esport Blanquerna, Universitat Ramon Llull, C/ Císter 34, 08022 Barcelona, Spain
2. Research group on Methodology, Methods, Models and Outcomes of Health and Social Sciences (M3O). Faculty of Health Science and Welfare. Centre for Health and Social Care Research (CESS). University of Vic-Central University of Catalonia (UVIC-UCC), C.Sagrada Família, 7, 08500 Vic, Spain

Rothenbacher, Dietrich
Institute of Epidemiology and Medical Biometry, Ulm University, 89081 Ulm, Germany.

Wirth, Katharina
1. Institute of Epidemiology and Medical Biometry, Ulm University, 89081 Ulm, Germany
2. Agaplesion Bethesda Clinic, Geriatric Research Unit Ulm University and Geriatric Center Ulm Alb-Donau, Zollernring 26, 89073 Ulm, Germany

Denkinger, Michael;
Agaplesion Bethesda Clinic, Geriatric Research Unit Ulm University and Geriatric Center Ulm Alb-Donau, Zollernring 26, 89073 Ulm, Germany

Deidda, Manuela

For peer review only

For peer review only - http://bmjopen.bmj.com/site/about/guidelines.xhtml

McIntosh, Emma

Health Economics and Health Technology Assessment (HEHTA), Institute of Health and Wellbeing (IHW), University of Glasgow, 1 Lilybank Gardens, Glasgow G12 8RZ, UK.

Gallardo Rodríguez, Rodrigo

Departamento de Ciencias del Deporte y Acondicionamiento Físico, CEADE, Universidad Católica de la Santísima Concepción, Concepción, Chile.

González, Denise

Lefebvre, Guillaume

1. Sport Initiative et Loisir Bleu Association, BP 1810467038 Strasbourg, Cedex, France.
2. Sport Initiative et Loisir Bleu Association, C/ Bailén 11 bajos, 08010 Barcelona, Spain

Word count (excluding title page, abstract, references, figures and tables): 3999

For peer review only - http://bmjopen.bmj.com/site/about/guidelines.xhtml

TITLE:

Exercise referral schemes enhanced by self-management strategies to reduce sedentary behaviour and increase physical activity among community-dwelling older adults from four European countries: protocol for the process evaluation of the SITLESS randomised controlled trial

ABSTRACT

Introduction: SITLESS is a randomised controlled trial determining whether exercise referral schemes can be enhanced by self-management strategies to reduce sedentary behaviour and increase physical activity in the long term, in community-dwelling older citizens. The intervention is complex and requires a process evaluation to understand how implementation, causal mechanisms and context shape outcomes. The specific aims are to: assess fidelity and reach of the implementation; understand the contextual aspects of each intervention site; evaluate the mechanisms of impact; and explore perceived effects.

Methods and analysis: Following the Medical Research Council guidance on complex interventions, a combination of qualitative and quantitative procedures are applied including: observational checklists and attendance registries, standardized scales (i.e. Marcus's Self-Efficacy Questionnaire, Physical Activity Self-Regulation Scale and the Lubben Social Network Scale) at baseline, post-intervention and follow-up assessments, semi-structured questionnaires gathering contextual characteristics and participant observations of the sessions. Semi-structured interviews and focus groups with the participants and trainers are conducted at post-intervention and during the follow-up to explore their experiences. Outcomes from the standardized scales are analysed as moderators within the impact evaluation. Descriptive results on context and perceived effects complement results on impact. The qualitative and quantitative findings will help to refine the logic model to finally support the interpretation of the results on the effectiveness of the intervention.

For peer review only - http://bmjopen.bmj.com/site/about/guidelines.xhtml

Ethics and dissemination: The study design was approved by the respective Ethical Committee of Ramon Llull University, Southern Denmark, Northern Ireland and Ulm University. Participation is voluntary, and all participants are asked to sign informed consent before starting the study.

A dissemination plan operationalizes how to achieve a social impact by reaching academic and non-academic stakeholders. A data management plan describes the specific data sets and regulates its deposition and curation. All publications will be open access.

Trial registration number: ClinicalTrials.gov, NCT02629666. Registered 19 November 2015.

Key words: Process Assessment (Health Care), Clinical Protocols, Clinical Trial, Health Behaviour, Qualitative Research.

STRENGTHS AND LIMITATIONS OF THIS STUDY:

1. Process evaluation is an essential part of the testing of the SITLESS complex intervention.
2. The Medical Research Council guidance on "Process evaluation of complex interventions", by Moore et al (2015) is used as a framework for conducting and reporting this process evaluation.
3. Mixed methods are used to address the specific aims and each aim is addressed by several methodological procedures applied with a complementary purpose.
4. Important information is identified about the differences and communalities regarding implementation, context and mechanisms of impact of a complex intervention aimed at increasing physical activity and reducing sedentary behaviour across intervention sites in four European countries.
5. Potential limitations are the reduced number of cases interviewed from the control arm, and the lack of in-depth analysis of the experiences of participants with lower adherence to the intervention and of those dropping out of the study.

For peer review only - http://bmjopen.bmj.com/site/about/guidelines.xhtml

For peer review only

INTRODUCTION

Randomized controlled trials have been traditionally focused on outcome evaluations by linking cause and effect and have been criticised as over simplistic for ignoring relevant aspects, such as the crucial role of context in shaping outcomes.[1] Therefore, the guidance on the design and evaluation of trials of complex interventions has emphasized the relevance of the processes to provide greater confidence in the conclusions about the effectiveness and thus improve how they inform future intervention development.[2] Complex interventions are composed by multiple active components that might for instance interact, be directed to change behaviour, allow a degree of tailoring or require a level of skills by those delivering and/or receiving the intervention. In addition, the effects of the intervention might be context-dependent and the causal pathway between the intervention and the outcome might be long, variable or include more than one causal pathway.[3]

The Medical Research Council (MRC) has recently proposed guidance to structure the process evaluation of clinical trials of complex interventions.[4,5] This framework is organized around three categories: i) context, ii) implementation and iii) mechanisms of impact, and considers their mutual relationships. This guideline recommends assessing fidelity and quality of implementation, clarifying causal mechanisms (i.e., to get a better understanding of the complex pathways between the intervention and the outcomes, and to identify unexpected mechanisms) and identifying contextual factors associated that might explain why different results could be found across sites. Likewise, the Context and Implementation of Complex Interventions (CICI) framework has been proposed to assess context in detail.[6] According to the CICI framework, context is defined as "anything external to the intervention that may act as a barrier or facilitator to its implementation, or its effect".[6] According to Moore et al., "complex interventions work by introducing mechanisms that are sufficiently suited to their context to produce change, while causes of problems targeted by interventions may differ from one context to another".[5] The CICI framework comprises three dimensions: context, implementation and setting, which interact with one another and with the

For peer review only - http://bmjopen.bmj.com/site/about/guidelines.xhtml

For peer review only

intervention dimension. Hence, *context* comprises seven domains: geographical, epidemiological, socio-cultural, socio-economic, ethical, legal and political. Whereas *setting* refers to the specific physical location, in which the intervention is put into practise.

In our current ageing societies, promoting physical activity has become one of the key health care policies for the prevention of age-related disability, risk reduction of non-communicable diseases and improvement of quality of life.[7–9] Moreover, sedentary behaviour has been established as a risk factor for mortality which is independent of low physical activity and reducing it, is increasingly becoming a relevant public health concern, especially for older adults.[10–13] Therefore, the SITLESS project was designed to determine whether the effects of exercise referral schemes (ERS) may be enhanced by self-management strategies (SMS), to reduce sedentary behaviour and increase physical activity with the final goal of long-term behaviour change, an improvement in health, physical function, quality of life as well as psychosocial outcomes in community-dwelling older European citizens. In order to achieve these goals, SITLESS was designed as a three-armed pragmatic randomised controlled trial, to assess the effectiveness and cost-effectiveness of existing physical activity interventions (ERS) enhanced with SMS, compared with ERS alone and also with general recommendations about physical activity (control group).[14,15] The ERS intervention aims to improve strength, aerobic capacity, and balance during 16 weeks, with two 60-minute sessions per week. The SMS consists of one one-to-one session, six group-based sessions and four telephone prompts to encourage behaviour change. Participants are followed-up for 12 and 18-months post-intervention. The control group receives two sessions consisting of general information and recommendations on healthy ageing. A total of 1,338 participants are expected to be included (446 in each arm).

The SITLESS intervention fulfils the definition of a complex intervention, mainly since it comprises different active components that allow for tailoring to the personal and local characteristics, it is aimed at changing behaviours and has a wide potential range of short and long-term physical, social

For peer review only - http://bmjopen.bmj.com/site/about/guidelines.xhtml

and psychological effects.[16] Accordingly, it was built upon a logic model presented in Figure 1 and the SITLESS trial requires a process evaluation as recommended.

Figure 1. The logic model of the SITLESS intervention.

In addition, for the SITLESS study, conducted in the European research framework Horizon 2020, it is a key issue to use the approach of Responsible Research Innovation (RRI). RRI is understood as an approach that anticipates and assesses potential implications and societal expectations with regard to research and innovation, with the aim to foster the design of inclusive and sustainable research and innovation.[17] RRI has been defined as an attempt to re-think research as a public good and thus conducted with and for society.[17] In practice, the SITLESS project has considered specifically public involvement, gender, ethics, open access and governance along the project.

Therefore, the SITLESS project has designed a process evaluation with the aim of understanding how implementation, causal mechanisms and the contextual factors shape the short and long-term outcomes. Primary outcomes of the trial include: (1) Sedentary behaviour as sitting time and time spent in activities which have been traditionally reported as requiring ≤ 1.5 MET, (2) physical activity as daily counts-per-minute and intensity of exercise performed, both objectively measured by ActiGraph® accelerometer. Furthermore, secondary outcomes include other health-related aspects, quality of life, function and psychosocial outcomes at post-intervention and 12-and 18-months follow-up. The specific aims of the process evaluation are firstly to assess the implementation of the intervention in terms of: a) fidelity to the intervention design, variability of what was delivered and how between intervention sites; and b) reach (adherence) of participants to the intervention. Secondly, to understand the role of context through the following: a) exploring whether contextual factors regarding the characteristics of the setting, facilitator, the neighbourhood, health care system, and other location specific aspects affect implementation and outcomes; b) assessing the variability and comparability between and within intervention sites; c) assessing the generalizability of the intervention's effectiveness results. Thirdly, the SITLESS project

For peer review only - http://bmjopen.bmj.com/site/about/guidelines.xhtml

aims to, understand the mechanisms of impact. Fourth and finally it aims to explore the perceived effects of the intervention.

The specific research questions corresponding to each aim are shown in **figure 2**.

Figure 2. The process evaluation framework for the SITLESS trial.

Figure 2 Legend: **** IDEA:** refers to the IDEA problem solving framework. [18]

METHODS AND ANALYSIS

Theoretical frameworks informing this process evaluation

The process evaluation of the SITLESS project follows the guidance on "Process evaluation of complex interventions" from the MRC.[5] According to this guidance, process evaluation is understood as an essential part of the design and testing of the SITLESS intervention. In addition, context will be specifically evaluated according to the CICI framework.[6]

The SITLESS process evaluation framework

Figure 2 illustrates the application of the MRC guidance to the SITLESS project. This figure highlights the research question in the categories of implementation, context, mechanisms of impact and perceived effects, and relates them with each methodological procedure applied. In addition, it shows the intervention characteristics and the outcomes.

Mixed methods used to address the specific aims

A combination of quantitative and qualitative procedures will be applied. This process evaluation was planned prior to implementation of the intervention, except for the semi-structured questionnaire on contextual data that was added more recently. Nevertheless, observational checklists, attendance registries and guidance for the qualitative procedures were tested with the first participants and improved accordingly to ease their use and capture the most relevant data.

For peer review only - http://bmjopen.bmj.com/site/about/guidelines.xhtml

Quantitative procedures quantify the fidelity to the intervention manual and the participants' adherence to the intervention through observational checklists and attendance registries, respectively. They also involve standardized scales to assess mediating factors (i.e., Marcus's Self-Efficacy Questionnaire (MSEQ)[19], the 12-item Physical Activity Self-Regulation Scale (PASR-12)[20] and the Lubben Social Network Scale (LSNS)[21]). Hence, contextual characteristics will be collected in a table format with prespecified topics (e.g., geographical aspects).

Qualitative procedures address the same research questions but according to participants' perceptions, trainers' experiences and researchers' observations. Qualitative procedures include participant observation, semi-structured interviews and focus groups and are detailed in **table 1**.

Qualitative methods require specific considerations regarding the research team, reflexivity and the study design.

A multi-country and multi-disciplinary research team including a variety of health professionals (medical doctors, epidemiologists, exercise physiologists, physiotherapists, etc.) will lead the analysis of the qualitative data gathered in Barcelona, Odense, Ulm and Belfast. All members of the team involved in the qualitative research will be encouraged to keep a research diary to record their reflexive notes, impressions of the data and thoughts about the analysis throughout the process. Researchers should remain flexible and adaptive throughout the process and not be attached to certainty to generate rich findings that could explain the complexity of social issues. Researchers with less experience in qualitative research will be trained to code, index, and chart data and to think reflexively about how their identities and experience affect the analysis process in order to help to interpret with accuracy the meaning and significance of the data.

We will apply purposeful sampling of participants with a maximum variation sampling method as a strategy to select a small number of cases that maximize the diversity relevant to the research question. By doing so, we aim to explore the differences and communalities regarding gender, functional level, ethnicity and socio-economic background, next to the type of intervention arm

For peer review only - http://bmjopen.bmj.com/site/about/guidelines.xhtml

(SMS+ERS group, ERS group and control group). Each qualitative procedure targets a specific purposeful sample of participants from each of the four intervention sites (Barcelona, Belfast, Odense and Ulm) according to the characteristics specified in **table 1**. Moreover, several procedures will be applied at different time-points (post-intervention and follow-up) to match with the relevant assessment time-points of the trial. The aims regarding context, mechanisms of impact and perceived effects will be explored at the end of intervention and both follow-up periods, whereas implementation is only assessed at the end of the intervention.

Appendixes 1 to 6 show examples of the templates used as observational checklists and attendance registries, and guidance for the qualitative procedures (semi-structured interviews and focus groups).

Each aim is addressed by several methodological procedures applied with a complementary purpose as illustrated in Figure 3 and described in detail below.

Figure 3. *Specific aims, procedures and outcomes of the process evaluation and their triangulation with impact evaluation outcomes*

Methods to evaluate aim 1:

Observational checklists: after each session of all SMS and ERS groups, the trainer records the degree of fulfilment of each task and each aim. Hence, the observational checklist of the ERS sessions also covers the implementation characteristics that might vary across intervention sites or across sessions (e.g., whether the sessions are conducted indoors or outdoors and whether and how the trainers tailored the ERS sessions to the frail participants).

Attendance registries: all SMS, ERS and control group sessions have a corresponding attendance registry recorded by the trainer after each session. It contains each participant's attendance, reasons for not attending and adverse events due to the intervention. The attendance is used to

For peer review only - http://bmjopen.bmj.com/site/about/guidelines.xhtml

calculate the adherence as an equivalent concept of dose and considers the compliance with the intervention (e.g., did only part of the exercises due to pain).

Qualitative procedures: semi-structured interviews and focus groups with participants will include questions about their perception of the implementation of the specific intervention elements. Focus groups with trainers will have specific questions on their perception of the implementation challenges. In addition, the participant observation of the SMS and ERS sessions by researchers will focus on the implementation of the intervention.

Methods to evaluate aim 2:

Prespecified contextual characteristics: the evaluation of this aim is addressed with a table with prespecified domains of context. Specifically, all intervention sites will provide information on the specific intervention setting and context. Setting characteristics will comprise of: Neighbourhood socioeconomic level (low/medium/high), urban/semi-rural/rural area, type of centres where the recruitment took place, ERS intervention, SMS intervention and the control group intervention takes place, existing ERS in the setting. This information will be collected by researchers at each intervention site. Data on context will include those geographical, epidemiological, socio-cultural, socio-economic, ethical, legal and political information that might be relevant as a barrier or facilitator to the intervention implementation, or its effect, for instance existing national programs on physical activity. These contextual data will be firstly collected by the leaders of each intervention site and complemented with the knowledge and views of the members of each local advisory board.

Qualitative procedures: semi-structured interviews and focus groups with participants and trainers include specific questions about the context. We will specifically explore the role of the physical environment and social context, with the latter comprising the personal circumstances, the personal network and the group dynamic of the SMS+ERS and ERS intervention arms.

Methods to evaluate aim 3:

For peer review only - http://bmjopen.bmj.com/site/about/guidelines.xhtml

Standardized scales: the MSEQ[19], PASR-12[20] and LSNS[21] are assessed within the baseline, post-intervention, and follow-up evaluations, to explore the role of self-efficacy, self-monitoring, goal setting, eliciting social support, reinforcements, time management, relapse prevention and social network as mediators of the decrease in sedentary behaviour and increase on physical activity as predicted by the SITLESS logic model (Figure 1). MSEQ has been shown to be a reliable, valid and accurate measure of self-efficacy for physical activity participation in populations with chronic diseases.[22,23] The PASR-12 has been shown to be a reliable and valid scale for the measurement of self-regulation for physical activity among older adults.[20] The LSNS is commonly used as a measure of social networks. It has high levels of internal consistency, stable factor structures, high correlations with criterion variables and clinical cut-points show good convergent validity among European community-dwelling older adults.[24]

Qualitative procedures: will be used to get a better understanding of complex causal pathways and unexpected mechanisms between intervention and outcomes. Specifically, semi-structured interviews with participants and focus groups with participants and trainers at the end of the intervention and at follow-up will include questions about perceived effects according to participants' experiences and trainers' observations. In addition, the participant observation of the SMS and ERS sessions by researchers will focus on how participants' actively respond and interact with the intervention.

Methods to evaluate aim 4:

Qualitative procedures: This aim is addressed through semi-structured interviews with participants and focus groups with participants and trainers at the end of the intervention and at follow-up. Perceived impacts will be considered as complementary to results of the effectiveness evaluation assessed with standardised scales.[14]

For peer review only - http://bmjopen.bmj.com/site/about/guidelines.xhtml

Within the RRI framework, the SITLESS process evaluation explores if there are differing responses to the intervention by disadvantage categories related with gender, disability, ethnicity, and socio-economic position. It also considers how these categories affect implementation and mechanisms of impact and, consequently, the effectiveness of the intervention.[17]

For peer review only

For peer review only - http://bmjopen.bmj.com/site/about/guidelines.xhtml

Table 1. Specific qualitative procedures, pre-selected criteria for the purposeful sampling,

**Qualitative procedure**	**Target**	**Pre-selected criteria for the purposeful sampling and numbers of each profile in each intervention site**	**Timeframe**
Participant observation	Participants in the SMS group	*Criteria:*<br>1 from low socio-economic neighbourhood (or medium, if no low)<br>1 medium neighbourhood (or high, if no low)	During the SMS intervention
Semi-structured individual interviews	Participants in the SMS group	*Criteria:* (priority low and medium socio-economic status)<br>1 frail man & 1 frail woman<br>1 robust man & 1 robust woman<br>1 for each ethnic minority (if applicable)	End of intervention
		Criteria: 1 man & 1 woman (priority is low-to-medium socio-economic status)	12 months follow-up<br>18 months follow-up
	Participants of the ERS group	*Criteria:* 1 man & 1 woman (priority is low-to-medium socio-economic status)	12 months follow-up<br>18 months follow-up
	Participants of the control group	*Criteria:* (priority is low-to-medium socio-economic status)<br>1 man<br>1 woman	End of intervention<br>12 months follow-up<br>18 months follow-up
Focus groups	Participants of the SMS intervention groups	*Criteria:*<br>1 low socio-economic neighbourhood (or medium, if no low)<br>1 medium neighbourhood (or high, if no low)	End of intervention
	Participants of the ERS intervention groups	1 (priority is low-to-medium socio-economic status)	End of intervention
	ERS and SMS trainers	1 with all trainers involved in the ERS and SMS training.	End of intervention

For peer review only - http://bmjopen.bmj.com/site/about/guidelines.xhtml

Analysis plan

Data from the process evaluation will be analysed according to two main procedures: quantitative and qualitative analyses.

Quantitative analysis:

Quantitative process evaluation data, recorded in the attendance registries and the observational checklists, will be used to conduct a descriptive analysis, overall and by intervention site. The *SITLESS observational checklists* will be used to calculate the percentage of fidelity of the implementation regarding tasks and aims of the SMS and ERS sessions in each intervention site. The adherence to the ERS, SMS and control group sessions will be calculated as a percentage of sessions attended with each respective attendance registry. In addition, this registry will allow estimating the frequency of reasons for not attending. Percentage of fidelity of the implementation and adherence will be compared across intervention sites to identify whether differences may exist between them. Comparison will be conducted applying analysis of variance models.

Specific data will be entered in the main study database. This is the case for the variable fidelity and for adherence that will allow us to assess whether a better implemented intervention or a higher dose of the intervention is related with more effect, respectively. The potential mediator role of adherence/dose, the MSEQ, the PASR-12 and the LSNS on the effect of the intervention on physical activity and sedentary behaviour will be explored by covariance analyses, adding these scales as covariates to the mixed linear models testing the effect of the intervention. The statistical efficacy analysis of SITLESS has been described previously.[14] The information collected in the prespecified table on context characteristics will be used to describe each intervention site.

For peer review only - http://bmjopen.bmj.com/site/about/guidelines.xhtml

Qualitative analysis:

The SITLESS qualitative data analysis will be based on the transcripts of the semi-structured interviews and focus groups, and the trainers' notes from the participant observation. The Framework Method will be applied to analyse these data.[25] The Framework Method belongs to the thematic analysis or qualitative content analysis. These approaches first identify commonalities and differences in qualitative data, and then focus on relationships between different parts of the data to draw descriptive and/or explanatory conclusions clustered around themes. Accordingly, we will apply a combined approach to the analysis, enabling themes to be developed both inductively from the accounts (experiences and views) of research participants and deductively from existing literature. Regular team meetings will facilitate our critical exploration of participant responses, discussion of deviant cases and agreement on recurring themes.

The analysis will be conducted following these steps: transcription, familiarisation with the interview, coding of the transcripts and diaries of the participant observation, developing a working analytical framework, applying the analytical framework, charting data into the framework matrix and interpreting the data.

The initial coding of the first four transcripts, one per intervention site, will be conducted by two independent researchers, adding coding labels, notes and ideas. As a next step, a working analytical framework will be developed by discussing codes assigned, similarities and differences to achieve an agreement on a set of codes to establish an initial analytical framework. This framework will be refined by coding of further manuscripts until no new codes are generated by two independent researchers. Next, the final analytical framework will be applied to the remaining transcripts. The use of a software to conduct the analysis will be discussed. Once all the data are coded, we will summarize the data in a matrix for each theme.[25] The matrix will be comprised of one row per participant and one column per code with a separate sheet for each

For peer review only - http://bmjopen.bmj.com/site/about/guidelines.xhtml

category, using also illustrative quotations into the corresponding cells. To interpret the data, themes are generated from the data set by reviewing the matrix and making connections within and between participants and categories. Lastly, analytical memos and team discussions will be used to develop themes aimed at offering possible explanations for what is happening within the data agreed by consensus.

Triangulation with impact evaluation outcomes

As shown in Fig. 3, outcomes of the process evaluation are triangulated with impact evaluation outcomes (i.e., quantitative outcomes on effectiveness) to support their interpretation thus reinforcing each other. Specifically, quantitative outcomes on fidelity, adherence and from standardized scales are analysed as moderators with the impact evaluation outcomes. Descriptive results on context and qualitative findings on perceived effects are compared with impact results to complement them. Qualitative results and the overall quantitative results will help to refine the logic model to finally support the interpretation of impact evaluation results.

Patient and public involvement

SITLESS, as a RRI project, has considered in the study design and involved from its onset different types of stakeholders as primary, secondary and tertiary end users.[14] Stakeholders comprise female and male older adults, representatives of older adults' associations, primary healthcare and sports professionals, policymakers and, where relevant, other local stakeholders such as health insurance. Specifically, a total of four local advisory boards were created at the beginning of the project, one in each intervention site (Barcelona, Odense, Belfast, Ulm). They met periodically with the local researchers' team and participated in a number of decisions of the project to consider patients' priorities and motivations, experience and preferences. Initially, a literature review was conducted, which included how older adults perceive PA and SB in order to design an intervention with the potential to achieve sustained changes of healthy behaviour. At each site, we initially explored older adults' experiences, preferences and priorities regarding

For peer review only - http://bmjopen.bmj.com/site/about/guidelines.xhtml

behaviour change through focus groups. Their views were used to adapt the intervention design. Local advisory boards contributed to the challenges faced regarding recruitment, retention of participants in the study and the dissemination actions. Once the intervention design was finished, a feasibility study was conducted in the four sites and evaluated with qualitative interviews to the participants. Their perceptions were gathered and used to improve the intervention for the definitive trial.

Qualitative procedures undertaken in the trial include questions to assess whether participants perceive any burden due to the intervention and to their participation in the study.

The dissemination plan comprises sharing the results of the trial at each health care, social or leisure centre involved in the recruitment and/or intervention to reach end users, health professionals and other relevant stakeholders. Further dissemination activities will be held to involve participants of each site as much as possible.

ETHICS AND DISSEMINATION

Ethics

The study design was approved by the Ethics and Research Committee of each intervention site: The Ethics and Research Committee of Fundació Blanquerna (Ramon Llull University, Spain), The Regional Committees on Health Research Ethics for Southern Denmark (University of Southern Denmark, Denmark), Office for Research Ethics Committees in Northern Ireland (ORECNI) (Queen's University of Belfast) and the Ethical Review Board of Ulm University (Ulm, Germany). Participation is voluntary, and all participants will be asked to sign informed consent before the start of the study.

Dissemination

The SITLESS dissemination plan operationalizes the main dissemination goal, which is to achieve a social impact by reaching academic and non-academic stakeholders, i.e., primary care and

For peer review only - http://bmjopen.bmj.com/site/about/guidelines.xhtml

health care professionals, older adults, policy makers, professionals related to physical activity promotion, etc. Specifically, process evaluation results will enhance social impact by providing implementation details that might be of interest for policy makers. In addition, qualitative results (e.g., quotations of participants) will support reaching non-academic stakeholders. A data management plan has been developed for the SITLESS study describing each specific dataset in the study and regulating its deposition and curation.

All publications from the SITLESS project, including those related with process evaluation, will be open access.

Discussion

The SITLESS project has developed a process evaluation to understand how implementation, causal mechanisms and the contextual factors shape the outcomes of a complex intervention that enhances ERS with SMS to reduce sedentary behaviour and increase physical activity among community dwelling older adults of four European countries. Accordingly, this trial has the purpose of facilitating future intervention development by informing policy-makers, practitioners and researchers about implementation, mechanisms of impact and contextual factors in detail. In case the intervention is proven as being effective and cost-effective, the information will be focused on the delivered intervention to allow replication in new contexts considering the relevant core components, context characteristics and implementation challenges. In case of being ineffective, process evaluation results will contribute to define whether the failure can be attributed to the intervention itself or to a poor implementation. Hence, process evaluation will provide useful data for systematic reviews to compare interventions considering their implementation and contextual characteristics.

Trial status

SITLESS started in May 2015. The feasibility study was conducted from December 2015 until June 2016. The recruitment for the trial started in July 2016. At the time of submission of this

For peer review only - http://bmjopen.bmj.com/site/about/guidelines.xhtml

protocol, all 1390 participants have been included in the study. To date, follow-up assessments are being conducted and are planned to finish by September 2019.

Author Contributions:

LCP, MT, MGG, PC, EM and DR contributed to the conception of the study. LCP, SB, KW, MS and NB contributed to the development of the specific procedures.

LCP will coordinate the overall development of the process evaluation. LCP, KW, MS and NB will coordinate the process evaluation in their respective intervention sites and LCP, SB, KW, MS and NB will contribute to the acquisition of process evaluation data.

LCP, SB, MT, PC, MGG, CM, FK, MSk, KW, NB, MDeid, EM, DR, RGR, JJ, OS, MSa; JW, MG, DG, GL, MDenk, AS and MR contributed to the design of the final process evaluation and will contribute to the analysis and interpretation of data and writing of the manuscript. LCP and MR contributed to the data management plan and analysis.

All authors provided feedback on the drafts of this paper and have read and approved the final manuscript.

Acknowledgements

We would like to thank the advisory board members from Barcelona, Odense, Ulm and Belfast and the participants of the feasibility study and the trial for their valuable contributions.

Data statement: Not applicable.

Funding:

This work was supported by the European Union program Horizon 2020 with the grant number H2020-Grant 634270.

Competing interests:

For peer review only

The authors declare that they have no competing interests.

Ethics approval:

The study design was approved by the Ethics and Research Committee of each intervention site: The Ethics and Research Committee of Ramon Llull University (reference number: 1314001P) (Fundació Blanquerna, Spain), The Regional Committees on Health Research Ethics for Southern Denmark (reference number: S-20150186) (University of Southern Denmark, Denmark), Office for Research Ethics Committees Northern Ireland (ORECNI reference number: 16/NI/0185) (Queen's University of Belfast) and the Ethical Review Board of Ulm University (reference number: 354/15) (Ulm, Germany). Participation is voluntary, and all participants will be asked to give written informed consent before the start of the study.

Open access

Not applicable.

For peer review only

For peer review only - http://bmjopen.bmj.com/site/about/guidelines.xhtml

REFERENCES

1. Berwick, D. M. The science of improvement. *JAMA - Journal of the American Medical Association* **299,** 1182–1184 (2008).

2. Campbell, N. C. *et al.* Designing and evaluating complex interventions to improve health care. *BMJ* **334,** 455–9 (2007).

3. Campbell, N. C. *et al.* Designing and evaluating complex interventions to improve health care. *BMJ* **334,** 455–9 (2007).

4. Moore, G., Audrey, S., Barker, M. & Bond, L. Process evaluation of complex interventions. *UK Med. Res. Counc. Guid. Prep.* 19-45; 64-75 (2014). doi:http://www.populationhealthsciences.org/MRC-PHSRN-Process-evaluation-guidance-final-2-.pdf

5. Moore, G. F. *et al.* Process evaluation of complex interventions: Medical Research Council guidance. *BMJ Br. Med. J.* **350,** 1–7 (2015).

6. Pfadenhauer, L. M. *et al.* Making sense of complexity in context and implementation: The Context and Implementation of Complex Interventions (CICI) framework. *Implement. Sci.* **12,** 1–17 (2017).

7. Lee, I. M. *et al.* Effect of physical inactivity on major non-communicable diseases worldwide: An analysis of burden of disease and life expectancy. *Lancet* **380,** 219–229 (2012).

8. Who, W. H. O. Global recommendations on physical activity for health. *Geneva World Heal. Organ.* 60 (2010). doi:10.1080/11026480410034349

9. Ding, D. *et al.* The economic burden of physical inactivity: a global analysis of major non-communicable diseases. *Lancet* **388,** 1311–1324 (2016).

10. Owen, N., Sparling, P. B., Healy, G. N., Dunstan, D. W. & Matthews, C. E. Sedentary behavior: Emerging evidence for a new health risk. *Mayo Clinic Proceedings* **85,** 1138–1141 (2010).

11. Koster, A. *et al.* Association of Sedentary time with mortality independent of moderate to vigorous physical activity. *PLoS One* **7,** (2012).

12. Harvey, J. A., Chastin, S. F. M. & Skelton, D. A. Prevalence of sedentary behavior in older adults: a systematic review. *Int. J. Environ. Res. Public Health* **10,** 6645–61 (2013).

13. Ekelund, U. *et al.* Does physical activity attenuate, or even eliminate, the detrimental association of sitting time with mortality? A harmonised meta-analysis of data from more than 1 million men and women. *Lancet* **388,** 1302–1310 (2016).

14. Giné-Garriga, M. *et al.* The SITLESS project: Exercise referral schemes enhanced by self-management strategies to battle sedentary behaviour in older adults: Study protocol for a randomised controlled trial. *Trials* **18,** (2017).

15. Deidda, M. & Coll-Planas, L. Cost effectiveness of exercise referral schemes enhanced by self-management strategies to battle sedentary behaviour in older adults: protocol

For peer review only - http://bmjopen.bmj.com/site/about/guidelines.xhtml

for an economic evaluation alongside the SITLESS three-armed pragmatic randomised clinical trial. *BMJ Open* **8,** (2018).

16. Lewin, S. *et al.* Assessing the complexity of interventions within systematic reviews: Development, content and use of a new tool (iCAT-SR). *BMC Med. Res. Methodol.* **17,** 1–13 (2017).

17. Bardone, E. & Lind, M. Towards a phronetic space for responsible research (and innovation). *Life Sci. Soc. Policy* **12,** 5 (2016).

18. Marcus, B. & Forsyth, L. *Motivating people to be physically active*. (Human Kinetics, 2009).

19. Marcus, B. H., Selby, V. C., Nlaura, R. S. & Rossi, J. S. Self-efficacy and the stages of exercise behavior change. *Res. Q. Exerc. Sport* **63,** 60–66 (1992).

20. Umstattd, M. R., Motl, R., Wilcox, S., Saunders, R. & Watford, M. Measuring physical activity self-regulation strategies in older adults. *J. Phys. Act. Health* **6 Suppl 1,** S105-12 (2009).

21. Lubben, J. E. Assessing social networks among elderly populations. *Fam. Community Health* **11,** 42–52 (1988).

22. Garratt, A. M., Løchting, I., Smedslund, G. & Hagen, K. B. Measurement properties of instruments assessing self-efficacy in patients with rheumatic diseases. *Rheumatology* **53,** 1161–1171 (2014).

23. Mendoza-Vasconez, A. S., Marquez, B., Benitez, T. J. & Marcus, B. H. Psychometrics of the self-efficacy for physical activity scale among a Latina women sample. *BMC Public Health* **18,** 1097 (2018).

24. Lubben, J. *et al.* Performance of an abbreviated version of the Lubben Social Network Scale among three European community-dwelling older adult populations. *Gerontologist* **46,** 503–13 (2006).

25. Gale, N. K., Heath, G., Cameron, E., Rashid, S. & Redwood, S. Using the framework method for the analysis of qualitative data in multi-disciplinary health research. *BMC Med. Res. Methodol.* **13,** 117 (2013).

Figure's legends:

Figure 1. The logic model of the SITLESS intervention.

**** IDEA:** refers to the IDEA problem solving framework. [18]

Figure 2. The process evaluation framework for the SITLESS trial based on the guidance on "Process evaluation of complex interventions" from the MRC.[5]

Figure 3. Specific aims, procedures and outcomes of the process evaluation and their triangulation with impact evaluation outcomes

For peer review only - http://bmjopen.bmj.com/site/about/guidelines.xhtml

APPENDIX

Appendix 1: SEMI-STRUCTURED QUESTIONNAIRE FOR INTERVIEWS WITH PARTICIPANTS OF THE SMS GROUP

Appendix 2: SEMI-STRUCTURED QUESTIONNAIRE FOR THE FOCUS GROUP FOR THE SMS INTERVENTION GROUPS

Appendix 3: OBSERVATIONAL CHECKLIST OF THE SMS INTERVENTION SESSIONS

Appendix 4: OBSERVATIONAL CHECKLIST OF THE PA SESSIONS

Appendix 5: ATTENDANCE REGISTRY FOR THE SMS SESSIONS

Appendix 6: ATTENDANCE REGISTRY FOR THE PA SESSIONS

For peer review only

For peer review only - http://bmjopen.bmj.com/site/about/guidelines.xhtml

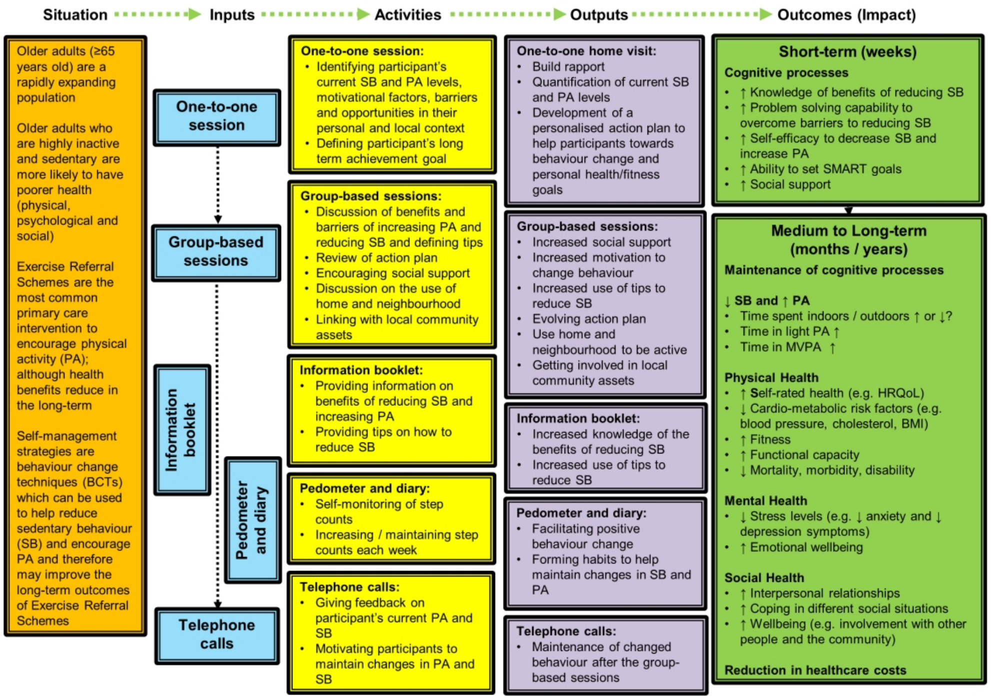

254x190mm (300 x 300 DPI)

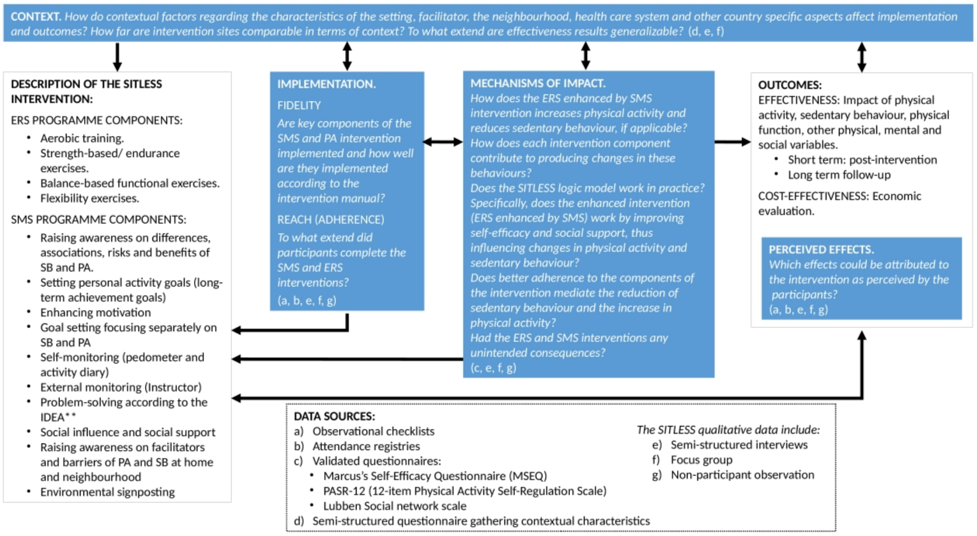

Figure 2. The process evaluation framework for the SITLESS trial based on the guidance on "Process evaluation of complex interventions" from the MRC.5

338x190mm (300 x 300 DPI)

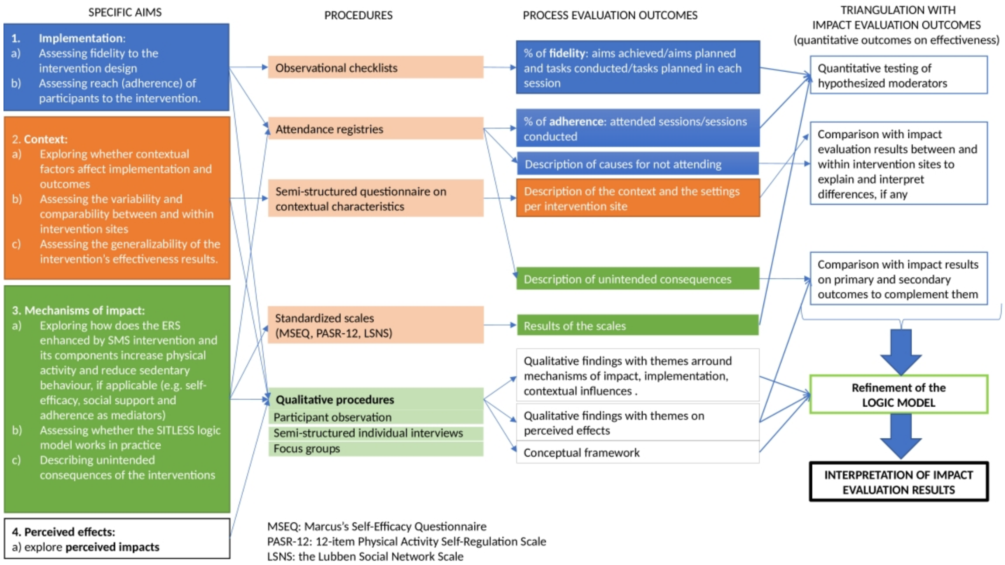

Figure 3. Specific aims, procedures and outcomes of the process evaluation and their triangulation with impact evaluation outcomes

338x190mm (300 x 300 DPI)

Appendix 1: SEMI-STRUCTURED QUESTIONNAIRE FOR INTERVIEWS WITH PARTICIPANTS OF THE SMS GROUP

Note: Aspects related with gender, frailty/robustness and ethnicity have been considered as elegibility criteria and will be considered in the content analysis. However, this questionnaire does not include any specific question regarding these aspects and are considered to emerge in their discourse.

Part one: introduction (5')

Section	Comments
**Introduction**	• Acknowledgements<br>• The conductor introduces briefly him/her self to the participant<br>• Inform about the duration and the aims of the meeting<br>• The conductor informs the participant that the meeting will be audio-recorded for later analysis.<br>• The conductor reminds the participant to turn-off or silence the phone.
**Informed consent**	• The conductor asks for permission about recording of the interview and informed consent.<br>• Reading and signing of informed consent.

For peer review only

Part two: overall mechanisms of impact (20’)

Dimension	Questions
*According to participant’ perceptions: how do the participant’ actively respond and interact with the **overall intervention**?*<br><br>**<u>Note:</u>** take into account that the SMS intervention requires following tasks:<br>○ *Fill the diary daily: steps or PA time, and calculate means.*<br>○ *Wear daily, open and read the pedometer.*<br>○ *Set goals on PA and follow-up accomplishment.*<br>○ *Set goals on SB and follow-up accomplishment (SITLESS tips).*<br>○ *Active participation in group discussions.*<br>○ *Telephone prompts*	• What are your general impression / thoughts of the intervention?<br>• Overall how satisfied are you with the intervention?<br>• What specific features of the intervention impressed you?<br>• What features of the intervention disappointed you?<br>• How likely would you be to recommend this intervention to a family member or friend? Why?<br>• What, if anything, do you find frustrating or unappealing about the intervention?<br>• Do you have any suggestions on how we could improve the intervention to make it more appealing?<br>• Were any of tasks required in the intervention difficult for you to perform?

For peer review only

Part three: overall perceived effects and mechanisms of impact (20')

Dimension	Questions
**Secondary outcomes**	• Have you perceived any effects of the intervention? Which ones?<br>• Have you perceived any specific effects on<br>○ General health?<br>○ Mental health? In terms of depression, loneliness, etc. and psychological/emotional wellbeing?<br>○ Physical health?<br>○ Social relationships?
**Primary outcomes: Effects on PA and SB**	
How does the SMS intervention produce change?	• During the intervention: have you increased your PA? (besides the ERS sessions)<br>• In case that you have increased your PA, why do you think you have done it?, Because your physical capacity increased<br>○ Because your self-efficacy increased<br>○ For other reasons<br>• In case that you have not increased your PA, why do you think you haven't done it?<br>• During the program: have you decreased your SB?<br>• If so, in which manner?<br>○ Incorporate new routines<br>○ Other
**Mid-long term effects on PA and SB**	• If not, why do you think that?<br>• Do you think you will maintain your increased PA / decreased SB once the intervention has finished?<br>• If so, in which manner?<br>• If not, why do you think that?

For peer review only

Part four: mechanisms of impact for each component (20')

Dimension	Questions
▶ *How do **each intervention components** contribute to produce change?*<br>▶ *Specifically, does the intervention work by improving self-efficacy and social support?*<br>▶ *Does a better accomplishment of the goal setting, self-monitoring and cues mediate the reduction of sedentary behaviour and the increase in physical activity?"*	• Can you state the strategies you have used to accomplish each session's goals? Have you accomplished them by yourself?<br>• What are the reasons why you have decided to accomplish (or not) the goals?<br>• How and why each component supported/permitted to achieve the goals of the intervention? (mechanisms of impact)<br>○ Raising awareness on differences, associations, risks and benefits of SB and PA.<br>○ Setting personal activity goals (long-term achievement goals)<br>○ Goal setting focusing separately on PA<br>○ Goal setting focusing separately on SB (SITLESS tips)<br>○ Self-monitoring:<br>▪ pedometer<br>▪ activity diary<br>○ External monitoring (Instructor)<br>○ Problem-solving<br>○ Raising awareness on facilitators and barriers of PA and SB at home and at the neighborhood (environmental signposting)<br>○ Peer and social support from the group<br>○ The trainer<br>○ Telephone prompts

For peer review only

Part five: context (20')

Dimension	Questions
**Physical environment**<br><br>*How do contextual factors regarding the characteristics of the neighbourhood affect outcomes according to participants' perceptions?*	• How do you perceive the physical environment of your neighbourhood?<br>• How do you perceive the specific settings of the intervention? (i.e., facilities used for the SMS and PA sessions)<br>○ Primary health care centre<br>○ Parks<br>○ Sports facilities<br>○ Community resources<br>• How do you perceive these specific aspects of your neighbourhood? (especially considering whether and how these aspects affect doing PA in the neighbourhood)<br>○ Socio-economic level<br>○ Urban/rural setting<br>○ Orography / walkability<br>○ Pleasant environment (comfortable, traffic…)<br>○ Personal safety (crimes, robberies, …)<br>○ Availability of places within an easy walking distance to do informal PA (parks, urban gym, …) and to do formal PA (organized PA offers)
**Personal networks**<br><br>*How do contextual factors regarding the characteristics of the personal network affect outcomes according to the participant's perceptions?*	• Do you think your personal situation or health status (e.g., having a supportive social network, being a caregiver, living alone, having a chronic disease, etc.) helps or hinders your physical activity and your sedentary behavior? How and why?<br>• Do you think your personal network (partners, family, friends) helps or hinders your physical activity and your sedentary behavior? How and why?<br>• Did you know other participants of the group before? How has that affected your participation in the group?

For peer review only

Appendix 2: SEMI-STRUCTURED QUESTIONNAIRE FOR THE FOCUS GROUP FOR THE SMS INTERVENTION GROUPS

Part one: introduction and presentation of participants (10')

Section	Comments
**Introduction**	• Acknowledgements<br>• The conductor introduces briefly him/her self to the participants<br>• Inform about the duration and the aims of the meeting<br>• The conductor informs the participants that the meeting will be audio-recorded for later analysis.<br>• The conductor reminds the participants to turn-off or silence phones.
**Informed consent**	• The conductor asks for permission about recording of the meeting and informed consent.<br>• Reading and signing of informed consent.
**Presentation of participants**	• Kindly ask participants to introduce their selves to the rest of the group.

For peer review only

Part two: overall mechanisms of impact (20')

Dimension	Questions
*According to participants' perceptions: how do participants' actively respond and interact with the **overall intervention**?*<br><br>**<u>Note:</u>** take into account that the SMS intervention requires following tasks:<br>○ *Fill the diary daily: steps or PA time, and calculate means.*<br>○ *Wear daily, open and read the pedometer.*<br>○ *Set goals on PA and follow-up accomplishment.*<br>○ *Set goals on SB and follow-up accomplishment (SITLESS tips).*<br>○ *Active participation in group discussions.*<br>○ *Telephone promots*	• What are your general impression / thoughts of the intervention?<br>• Overall how satisfied are you with the intervention?<br>• What specific features of the intervention impressed you?<br>• What features of the intervention disappointed you?<br>• How likely would you be to recommend this intervention to a family member or friend? Why?<br>• What, if anything, do you find frustrating or unappealing about the intervention?<br>• Do you have any suggestions on how we could improve the intervention to make it more appealing?<br>• Were any of tasks required in the intervention difficult for you to perform?

For peer review only

Part three: overall perceived effects and mechanisms of impact (20')

Dimension	Questions
**Secondary outcomes**	• Have you perceived any effects of the intervention? Which ones?<br>• Have you perceived any specific effects on<br>o General health?<br>o<br>o Mental health? In terms of depression, loneliness, etc. and psychological/emotional wellbeing?<br>o Physical health?<br>o Social relationships?
**Primary outcomes: Effects on PA and SB**<br><br>*How does the SMS intervention produce change?*	• During the intervention: have you increased your PA? (besides the ERS sessions)<br>• In case that you have increased your PA, why do you think you have done it?, Because your physical capacity increased<br>o Because your self-efficacy increased<br>o For other reasons<br>• In case that you have not increased your PA, why do you think you haven't done it?<br>• During the program: have you decreased your SB?<br>• If so, in which manner?<br>o Incorporate new routines<br>o Other<br>• If not, why do you think that?
**Mid-long term effects on PA and SB**	• Do you think you will maintain your increased PA / decreased SB once the intervention has finished?<br>• If so, in which manner?<br>• If not, why do you think that?

For peer review only

Part four: mechanisms of impact for each component (20')

Dimension	Questions
• *How do **each intervention components** contribute to produce change?*<br>• *Specifically, does the intervention work by improving self-efficacy and social support?*<br>• *Does a better accomplishment of the goal setting, self-monitoring and cues mediate the reduction of sedentary behaviour and the increase in physical activity?"*	• Can you state the strategies you have used to accomplish each session's goals? Have you accomplished them by yourself?<br>• What are the reasons why you have decided to accomplish (or not) the goals?<br>• How and why each component supported/permitted to achieve the goals of the intervention? (mechanisms of impact)<br>○ Raising awareness on differences, associations, risks and benefits of SB and PA.<br>○ Setting personal activity goals (long-term achievement goals)<br>○ Goal setting focusing separately on PA<br>○ Goal setting focusing separately on SB (SITLESS tips)<br>○ Self-monitoring:<br>▪ pedometer<br>▪ activity diary<br>○ External monitoring (Instructor)<br>○ Problem-solving<br>○ Raising awareness on facilitators and barriers of PA and SB at home and at the neighborhood (environmental signposting)<br>○ Peer and social support from the group<br>○ The trainer<br>○ Telephone prompts

For peer review only

Part five: context (20')

Dimension	Questions
**Physical environment**<br><br>*How do contextual factors regarding the characteristics of the neighbourhood affect outcomes according to participants' perceptions?*	• How do you perceive the physical environment of your neighbourhood?<br>• How do you perceive the specific settings of the intervention? (i.e., facilities used for the SMS and PA sessions)<br>○ Primary health care centre<br>○ Parks<br>○ Sports facilities<br>○ Community resources<br>• How do you perceive these specific aspects of your neighbourhood? (especially considering whether and how these aspects affect doing PA in the neighbourhood)<br>○ Socio-economic level<br>○ Urban/rural setting<br>○ Orography / walkability<br>○ Pleasant environment (comfortable, traffic…)<br>○ Personal safety (crimes, robberies, …)<br>○ Availability of places within an easy walking distance to do informal PA (parks, urban gym, …) and to do formal PA (organized PA offers)
**Personal networks**<br><br>*How do contextual factors regarding the characteristics of the personal network affect outcomes according to participants' perceptions?*	• Do you think your personal situation or health status (e.g., having a supportive social network, being a caregiver, living alone, having a chronic disease, etc.) helps or hinders your physical activity and your sedentary behavior? How and why?<br>• Do you think your personal network (partners, family, friends) helps or hinders your physical activity and your sedentary behavior? How and why?<br>• Did you know other participants of the group before? How has that affected your participation in the group?

For peer review only

Appendix 3: OBSERVATIONAL CHECKLIST OF THE SMS INTERVENTION SESSIONS

Centre: Group: Trainer:

Date: Session number: 2	
Week: 3	Stage: Ramping

FIRST GROUP-BASED SESSION Length of session: ______ minutes

Tasks	Completed	Partially completed	Not completed
**Activity 1** - Identifying the benefits of being less sedentary and more active.			
**Activity 2** - Share and discuss personal lifestyles related to sedentary behaviour, physical activity and physical exercise.			
**Activity 3** - Share and discuss tips for sitting less			
**Activity 4** - Retrieve the participants' baseline daily steps and/or weekly time in physical activities / exercise and discuss the reasons for differences between certain days.			
**Activity 5** - Agree on which SITLESS tips the participants will try over the next week.			
**FINAL TASKS**<br>• Make a statement to encourage the group to practise the SITLESS tips:<br>• The final tasks are to arrange a date/time for the next session and to remind the participants to bring their information booklets and activity diaries			

Aims of the session	Completed	Partially completed	Not completed
1. To identify the benefits of being less sedentary and more active.			
2. To share and discuss personal lifestyles related to sedentary behaviour, physical activity and physical exercise			
3. To share and discuss tips for sitting less.			
4. To retrieve the participants' baseline daily steps and/or weekly time in physical activities / exercise and discuss the reasons for differences between certain days.			
5. To agree on which SITLESS tips the participants will try over the next week.			

For peer review only

Centre: Group: Trainer:

Date:	Session number:	
	Week:	Stage:

Which tasks went particularly well during the session? Why?

Were any tasks difficult to implement? Why?

Were any modifications made to the planned session? Why? *(adhoc tailoring)*

Have you observed any adverse effects? Which ones?

Generally, what was the participant's response / attitude to the different activities (e.g. uninterested, excitable, attentive, sceptical, quiet, chatty)? Have you observed specific responses to be highlighted regarding gender, educational level, ethnicity..? Which ones?

Other comments

For peer review only

Appendix 4: OBSERVATIONAL CHECKLIST OF THE PA SESSIONS

Note: This annex contains the standard Observation Checklist of the PA sessions with all the information needed from each intervention site. This checklist has been adapted by each intervention site in order to avoid repetition by keeping track only of aspects of the PA interventions that may vary from session to session. E.g., Odense is conducting all sessions indoors and trainers do not need to register this aspect after each session.

Centre: Group: Trainer:

Date: Session number:

This checklist should be filled in by the PA instructor after EACH physical activity session

DATA ON EACH SESSION REPORTED BY THE INSTRUCTOR

1. Where have you conducted the physical activity session?

Indoors/outdoors Specific setting:___________

2. Has the climate / environment affected this session? *(for an individual / overall group)* YES/NO

How? ____________________________________

3. Was this session conducted by the usual instructor? YES/NO
4. Have you tailored the physical activity session to the frail participants? YES/NO

How?____

5. Have you personalized the progression in the exercise intensity? YES/NO

How?____

6. Have you tailored the physical activity session to specific participants for other reasons? YES/NO

How? ____

7. Use of equipment/ material:

Indicate the use of weight lifting equipment	Yes	No
Indicate the use of cardiovascular machines	Yes	No
Indicate the use of other material (e.g. therabands, balls, bosu)	Yes<br>If yes, which:_______<br>__________________	No
Indicate the use of recycled/ adapted material	Yes	No
Indicate the use of music	Yes	No

For peer review only

8. Have you applied any group dynamics to promote group cohesion? (e.g., cooperative games, work in pairs...)

How? ____

9. Every second session, use the final 5 minutes of session to ask the participants whether they have done the extra Physical Activity session on their own per week (defined as at least 30 minutes of moderate or vigorous PA following the WHO guidelines, for instance brisk walking)

Number of participants that had done the extra Physical Activity session/ out of the total attending → calculate percentage:

………………../………………: ____%

10. Observations relevant for the study (e.g. gender specific issues?)

Please add here observations on the development of the session. Add the participant code to refer to observations of specific participants

……

……

……

……

……

……

For peer review only

Appendix 5: ATTENDANCE REGISTRY FOR THE SMS SESSIONS

Centre: Group: Trainer:

		Dates of the sessions (dd/mm/yy)										
**Participant code**	**Participant name**	One-to-one	1st group session	2nd group session	3rd group session	4th group session	5th group session	6th group session	1st phone call	2nd phone call	3rd phone call	4th phone call
**EXAMPLE:** CL004	Maria Roca	√ 01.02.16	√	¥ B	√	√	√	√	√ 5/05/16	√ 6/07/16	∏ E 01-15/10/16	∏ E 01-15/01/17

√ = Attendance. In case a certain participant arrived considerably late or performed only part of the session (e.g., arrived 20 minutes late or did half of the exercises due to pain), report the % performed of the session.

¥ = Unexcused absence (e.g.., the trainer was NOT previously informed)

∏ = Excused absence (e.g.., the trainer was previously informed)

For peer review only

For peer review only - http://bmjopen.bmj.com/site/about/guidelines.xhtml

Appendix 5: ATTENDANCE REGISTRY FOR THE SMS SESSIONS

Centre: Group: Trainer:

Please, state the reason for non-attendance: (A) health problem (cold, illness, surgery, etc), (B) medical visit, (C) trip/travel, (D) familiar reason (taking care of grandsons, etc), (E) other (specify).

Please, state any **adverse effect** for participant and session (e. g. anxiety).

Please, fill the table in case you need to specify the reason for non-attendance or the adverse effect. Include those objective or perceived adverse effects that happened during this SMS session, that participants and/or the trainer attribute to the SMS intervention.

**Participant code**	**Date**	**Note of reasons for non-attendance**	**Note on adverse effect**
CL004	01-15/10/16	Participant not reached after repeated calls	
CL004	01-15/01/17	Participant not reached after repeated calls	

For peer review only

Appendix 6: ATTENDANCE REGISTRY FOR THE PA SESSIONS

Centre: Group:

Trainer:

			Dates of the sessions (dd/mm/yy)															
**Participant code**	**Participant name**	**SPPB**	01.02.16	03.02.16	08.02.16	10.02.16	15.02.06	17.02.16										
**EXAMPLE:** CL004	Maria Roca	L	√	X	∏ B	√	√ 2 50%	∏ E										

√ = Attendance. In case a certain participant arrived considerably late or performed only part of the session (e.g., arrived 20 minutes late or did half of the exercises due to pain), report the % performed of the session.

X = Unexcused absence (i.e., the trainer was NOT previously informed)

∏ = Excused absence (i.e., the trainer was previously informed)

Please, state the **SPPB** (Short Physical Performance Battery) as L (low), M (medium), or H (high)

Please, state any **adverse effect** of physical activity (PA) for participant and session, according to following codes: (1) stiffness after PA, (2) joint pain, (3) fall during PA session, (4) dizziness, (5) other (specify)

For peer review only

Please, state the **reason for non-attendance**: (A) health problem (cold, illness, surgery, etc.), (B) medical visit, (C) trip/travel, (D) familiar reason (taking care of grandsons, etc.), (E) other (specify)

Please, fill the table in case you need to specify the reason for non-attendance or the adverse effect. Include those objective or perceived adverse effects that participants and/or the trainer attribute to the PA session.

Participant code	Date	Note of reasons for non-attendance	Note on adverse effect
CL004	17.02.16	Forgot the session	

For peer review only
