# Supplementary material for: Exercise referral schemes enhanced by self-management strategies to reduce sedentary behaviour and increase physical activity among community-dwelling older adults from four European countries: protocol for the process evaluation of the SITLESS randomised controlled trial
Source: BMJ Open. 2019 Jun 14;9(6):e027073. doi: 10.1136/bmjopen-2018-027073 (PMC6588992; doi:10.1136/bmjopen-2018-027073)
Supplement: Reviewer comments [file bmjopen-2018-027073.reviewer_comments.pdf]

PEER REVIEW HISTORY

BMJ Open publishes all reviews undertaken for accepted manuscripts. Reviewers are asked to complete a checklist review form (**http://bmjopen.bmj.com/site/about/resources/checklist.pdf**) and are provided with free text boxes to elaborate on their assessment. These free text comments are reproduced below.

ARTICLE DETAILS

**TITLE (PROVISIONAL)**	Exercise referral schemes enhanced by self-management strategies to reduce sedentary behaviour and increase physical activity among community-dwelling older adults from four European countries: protocol for the process evaluation of The SITLESS randomised controlled trial
**AUTHORS**	Coll-Planas, Laura; Blancafort Alias, Sergi; Tully, Mark; Caserotti, Paolo; Giné-Garriga, Maria; Blackburn, Nicole; Skjødt, Mathias; Wirth, Katharina; Deidda, Manuela; McIntosh, Emma; Rothenbacher, Dietrich; Gallardo Rodríguez, Rodrigo; Jerez-Roig, J.; Sansano-Nadal, Oriol; Santiago, Marta; Wilson, Jason; Guerra-Balic, Míriam; Martín-Borràs, Carme; Gonzalez, Denise; Lefebvre, Guillaume; Denkinger, Michael; Kee, Frank; Salvà Casanovas, Antoni; Roqué i Figuls, Marta

VERSION 1 - REVIEW

**REVIEWER**	Alyt Oppewal<br>Erasmus MC, University Medical Center Rotterdam, the Netherlands
**REVIEW RETURNED**	13-Nov-2018

**GENERAL COMMENTS**	Exercise referral schemes enhanced by self-management strategies to reduce sedentary behaviour and increase physical activity among community dwelling older adults from four European countries: protocol for the process of evaluation of The SITLESS randomised controlled trial.<br><br>The authors are complimented for addressing this important topic. Low levels of physical activity and high levels of sedentary behaviour are a major health hazard. However, improving activity levels and reducing sedentary behaviour has been proven to be very difficult, especially in older adults. This study will provide important information for improving the effect of interventions within this area.<br><br>Introduction<br>The introduction is clearly written, however the research questions need some clarifications:<br>- Page 10, line 28: The research question 'How does the SMS intervention produce change?' does not specify in what area the authors want to evaluate change. This should be clarified and added to the research question.<br>- Page 10, line 30: The second research question 'How does each intervention component contribute to producing changes in behaviour' also does not specify what kind of

	behaviour. Is that only sedentary and physical activity behaviour, or also other kinds of behaviour, social behaviour for example?<br>- Page 10, line 32: With regard to the question ‘Does the intervention work by improving self-efficacy and social support?’, do the authors want to evaluate the intervention as a whole or also the components ERS and SMS separately?<br>Some minor points with regard to the introduction:<br>- Page 10, line 12: The term PA intervention has not been used previously and I think it has to be changed to ERS.<br>- Page 10: The bullets for the first two objectives are different than those of the last two objectives. I would suggest to number the aims instead of using bullets, as has been done in the text and in the method section as well.<br>- Page 10, line 39-41: Looking at the other research questions, this font should be in italic.<br><br>Methods and analysis<br>This study has a lot of aims and accompanying research questions. In the method section, the methods are discussed per aim. However, the aims have multiple research questions. Because the methods and outcome measures are not specified per research question it is now difficult to understand which outcome measures are used to answer the research questions. Therefore it is not directly clear how all questions will be answered. I would therefore suggest to specify the outcome measures per research question, within each aim. A table summarizing the methods and outcome measures used for each research question may also help to clarify this.<br><br>There is little information provided about the intervention itself. This would be very helpful to understand what is exactly being evaluated. For example, how long is the intervention? How long is the follow-up period? How many participants are included in total and per study arm? How often are the specific sessions provided? I understand that the full description of the intervention is described in reference 13 (Giné-Garriga et al. 2017), however for the readability of the article some of this information should also be mentioned here.<br><br>With regard to the statistical analyses (page 17) some points are unclear:<br>- Line 15-21: Which methods are used to assess differences?<br>- Line 39-44: The authors mention that the scales “will be analysed within the main database to test their potential role as mediating the effect of the intervention on physical activity and sedentary behaviour.” However, it is unclear how this will be analyses. Specify which statistical methods will be used to answer this question.<br>Some minor points with regard to the method section:<br>- Page 10, line 53: The abbreviation MRC has already been introduced in the introduction. Introduce the abbreviation once, and then use the abbreviation throughout the rest of the manuscript.<br>- Page 11, line 14: The aim ‘Perceived effects’ is missing here.<br>- Page 12, line 28-33: Again the aim regarding ‘Perceived effects’ is missing here.<br>- Page 14, line 48-50: Provide information about the psychometric properties of these scales, and in which population

	this has been studied. This information is needed to understand the suitability of these scales for this study.

**REVIEWER**	Dustin Scott Kehler<br>Dalhousie University
**REVIEW RETURNED**	15-Nov-2018

**GENERAL COMMENTS**	Protocol summary:<br><br>This article describes a protocol for a process evaluation of the SITLESS RCT. The authors aim to use the Medical Research Council (MRC) guidance to evaluate the intervention, specifically to assess the delivery quality and quantity of the trial, generalizability of effectiveness and to explore the mechanisms of impact and perceived impact of the program.<br><br>Strengths:<br><br>-The authors have chosen a robust tool (MRC guidance) to evaluate their complex intervention.<br><br>Major comments:<br><br>- The authors seek to assess the quantity and quality of what was delivered, but “quantity” and “quality” are not sufficiently defined.<br><br>-There is a rich, valuable amount of information that will come from the study participants themselves. However, little data are collected from those delivering the trial and no data are derived from other potential knowledge-users/stakeholders who may have had a large hand in developing the project. Neglecting to collect information from these key personnel seems to be a missed opportunity for this process evaluation. As such, the authors are recommended to provide a rationale (or to now include in data collection/analysis) for not including stakeholders in their process evaluation of SITLESS.<br><br>-In general, the methods need to be described in more detail to understand the process evaluation procedures. For example, the non-expert reader of the manuscript may not know what “maximal variation” is when purposive sampling. Furthermore, Responsible Research Innovation, or the Framework Method may not be known to many readers.<br><br>-The authors plan to analyze the qualitative and quantitative findings separately for each aim. It seems like there is a missed opportunity here to provide an enriched understanding of the contextual factors that led to the success (or failure) of the program. Methods such as triangulation may be considered to understand the relationship between the quantitative (e.g., mediators of the efficacy of SITLESS with the qualitative findings from the intervention/control participants).<br>Comments specific to sections in the manuscript:

	Abstract:<br>-The authors are recommended to provide some information regarding the analysis plan/mixed methods approach of the process evaluation. At this point the authors only describe the tools that are being used.<br><br>Introduction:<br><br>-Page 7, lines 46-51: "Therefore, the SITLESS project has designed a process evaluation with the aim of understanding how implementation, causal mechanisms and the contextual factors shape the short and long term outcomes." – it is suggested that the outcomes of SITLESS should be described at some point in the introduction to provide context for this statement.<br><br>-Page 7, lines 53-55: "2) assess the generalizability of the effectiveness…" – is this the effectiveness of the intervention to reduce sitting time and increase physical activity? Or the effectiveness of delivery of the intervention? Please clarify.<br><br>Methods:<br>-See comment above regarding the methods section.<br><br>-In light of most of the data being collected long after delivery of SITLESS, the authors are recommended to clarify whether this process evaluation was planned prior to implementation or was an ad-hoc plan.<br><br>-Page 9, lines 29-31: "…involve standardized scales to assess mediating factors.." – What are the standardized scales?<br><br>-It is recommended that an appendix with semi-structured interview and focus group questions be provided. Similarly, an appendix of the observation checklist and attendance registry be provided to provide the readers with a better understanding of data collection.<br><br>-Regarding the qualitative procedures for aim 3: it is recommended to describe how the information from quantitative mediating factors (i.e., self-efficacy, self-regulation, social networks) will be complimented or analyzed together with the qualitative data.<br><br>-The statistical analysis procedures for the quantitative data (e.g., mediating factors of self-efficacy, self-regulation, social networks) should be detailed.<br><br>Tables and figure:<br><br>-Some of the text is a little bit difficult to read and sometimes gets cut off at the bottom or the top. Likely this will be fixed with publications. Also Figure 1 seems to be cut off at the bottom of the activities and outputs sections. Please revise.

VERSION 1 – AUTHOR RESPONSE

Reviewer(s)' Comments to Author:
Reviewer: 1

Reviewer's comments	Response
- The authors seek to assess the quantity and quality of what was delivered, but "quantity" and "quality" are not sufficiently defined.	This sentence was changed to improve clarification for: "the implementation of the intervention in terms of: a) fidelity to the intervention design, variability of what was delivered and how between intervention sites; and b) reach (adherence) of participants to the intervention."
little data are collected from those delivering the trial and no data are derived from other potential knowledge-users/stakeholders who may have had a large hand in developing the project. Neglecting to collect information from these key personnel seems to be a missed opportunity for this process evaluation. As such, the authors are recommended to provide a rationale (or to now include in data collection/analysis) for not including stakeholders in their process evaluation of SITLESS.	We have included in the statement "Patient and Public involvement" how stakeholders are involved along the study and we included in the methods corresponding to aim 2 how the views of leaders of the intervention sites and local stakeholders will be gathered to assess the context of the intervention.
-In general, the methods need to be described in more detail to understand the process evaluation procedures. For example, the non-expert reader of the manuscript may not know what "maximal variation" is when purposive sampling.<br>Furthermore, Responsible Research Innovation, or the Framework Method may not be known to many readers.	The terms maximal variation, RRI and Framework Method are explained. We have also added detail throughout to help improve the readers understanding of the process evaluation procedures as following:<br><br>"maximum variation sampling method as a strategy to select a small number of cases that maximize the diversity relevant to the research question"<br><br>"In addition, for the SITLESS study, conducted in the European research framework Horizon 2020, it is a key issue to use the approach of Responsible Research Innovation (RRI). RRI is understood as an approach that anticipates and assesses potential implications and societal expectations with regard to research and innovation, with the aim to foster the design of inclusive and sustainable research and innovation.[17] RRI has been defined as an attempt to re-think research as a public good and thus conducted with and for society;.[17] In practice, the SITLESS project has considered specifically public involvement, gender,

	ethics, open access and governance along the project."<br><br>"The Framework Method belongs to the thematic analysis or qualitative content analysis."

-The authors plan to analyze the qualitative and quantitative findings separately for each aim. It seems like there is a missed opportunity here to provide an enriched understanding of the contextual factors that led to the success (or failure) of the program. Methods such as triangulation may be considered to understand the relationship between the quantitative (e.g., mediators of the efficacy of SITLESS with the qualitative findings from the intervention/control participants).<br>Abstract:<br>-The authors are recommended to provide some information regarding the analysis plan/mixed methods approach of the process evaluation. At this point the authors only describe the tools that are being used.	In the abstract, in a new section called "Triangulation with impact evaluation outcomes" and in the figure 3 now called Research questions, methods adopted and outcomes for the process evaluation of the SITLESS trial. We also have clarified the mixed methods approach and how we will combine the qualitative and quantitative outcomes from the process evaluation and triangulate these outcomes with those from impact evaluation.<br>New text:<br><br>"Triangulation with impact evaluation outcomes<br>As shown in Fig. 3, outcomes of the process evaluation are triangulated with impact evaluation outcomes (i.e., quantitative outcomes on effectiveness) to support their interpretation thus reinforcing each other. Specifically, quantitative outcomes on fidelity, adherence and from standardized scales are analysed as moderators with the impact evaluation outcomes. Descriptive results on context and qualitative findings on perceived effects are compared with impact results to complement them. Qualitative results and the overall quantitative results help to refine the logic model to finally support the interpretation of impact evaluation results."
Introduction:<br>-Page 7, lines 46-51: "Therefore, the SITLESS project has designed a process evaluation with the aim of understanding how implementation, causal mechanisms and the contextual factors shape the short and long term outcomes." – it is suggested that the outcomes of SITLESS should be described at some point in the introduction to provide context for this statement.	As suggested, outcomes of the study have been added to the introduction section as following:<br>"Primary outcomes of the trial include: (1) Sedentary behavior as sitting time and time spent in activities which have been traditionally reported as requiring ≤ 1.5 MET, (2) physical activity as daily countsper-minute and intensity of exercise performed, both objectively measured by ActiGraph® accelerometer. Furthermore, secondary outcomes include other healthrelated aspects, quality of life, function and

	psychosocial outcomes at post-intervention and 12-and 18-months follow-up."
Introduction:<br>Page 7, lines 53-55: "2) assess the generalizability of the effectiveness…" – is this the effectiveness of the intervention to reduce sitting time and increase physical	In this sentence, we meant the effectiveness of the intervention to reduce sitting time and increase physical activity. This has been now clarified in the text as following: "assessing the generalizability of the intervention's effectiveness results"

activity? Or the effectiveness of delivery of the intervention? Please clarify.	
Methods:<br>-In light of most of the data being collected long after delivery of SITLESS, the authors are recommended to clarify whether this process evaluation was planned prior to implementation or was an ad-hoc plan.	As now explained in the text, this process evaluation was planned prior to implementation of the intervention, except for the semi-structured questionnaire on contextual data that was added more recently. Nevertheless, observation checklists, attendance list and guidance for the qualitative procedures were pilot-tested with the first participants and improved accordingly to ease their use and capture most relevant data.<br>New text:<br><br>"This process evaluation was planned prior to implementation of the intervention, except for the semi-structured questionnaire on contextual data that was added more recently."
Methods:<br>-Page 9, lines 29-31: "…involve standardized scales to assess mediating factors.." – What are the standardized scales?	Scales have been listed to the text now.<br>New text:<br>"They also involve standardized scales to assess mediating factors (i.e., Marcus's SelfEfficacy Questionnaire (MSEQ)[18], the 12item Physical Activity Self-Regulation Scale (PASR-12)[19] and the Lubben Social Network Scale (LSNS)[20])."
Methods:<br>-It is recommended that an appendix with semi-structured interview and focus group questions be provided. Similarly, an appendix of the observation checklist and attendance registry be provided to provide the readers with a better understanding 67of data collection.	We have added the following six appendixes as examples of our templates and guidance for the qualitative procedures:<br>Appendix 1: SEMI-STRUCTURED QUESTIONNAIRE FOR INTERVIEWS WITH PARTICIPANTS OF THE SMS GROUP<br>Appendix 2: SEMI-STRUCTURED QUESTIONNAIRE FOR THE FOCUS GROUP FOR THE SMS INTERVENTION GROUPS<br>Appendix 3: OBSERVATION CHECKLIST OF THE SMS INTERVENTION

	SESSIONS<br>Appendix 4: OBSERVATION CHECKLIST OF THE PA SESSIONS<br>Appendix 5: ATTENDANCE LIST FOR THE SMS SESSIONS<br>Appendix 6: ATTENDANCE LIST FOR THE PA SESSIONS

Methods:<br>-Regarding the qualitative procedures for aim 3: it is recommended to describe how the information from quantitative mediating factors (i.e., self-efficacy, self-regulation, social networks) will be complimented or analyzed together with the qualitative data.	In the methods section, we have added a new subsection called "Triangulation with impact evaluation outcomes" and in the figure 3 now called Research questions, methods adopted and outcomes for the process evaluation of the SITLESS trial we have clarified the mixed methods approach and how we triangulate the outcomes of the process evaluation with those from impact evaluation.<br><br>New text:<br><br>"Triangulation with impact evaluation outcomes<br>As shown in Fig. 3, outcomes of the process evaluation are triangulated with impact evaluation outcomes (i.e., quantitative outcomes on effectiveness) to support their interpretation thus reinforcing each other. Specifically, quantitative outcomes on fidelity, adherence and from standardized scales are analysed as moderators with the impact evaluation outcomes. Descriptive results on context and qualitative findings on perceived effects are compared with impact results to complement them. Qualitative results and the overall quantitative results help to refine the logic model to finally support the interpretation of impact evaluation results."
Methods:<br>-The statistical analysis procedures for the quantitative data (e.g., mediating factors of self-efficacy, self-regulation, social networks) should be detailed.	We have changed the original text "The MSEQ, the PASR-12 and the Lubben Social Network Scale will be analysed within the main database to test their potential role as mediating the effect of the intervention on physical activity and sedentary behaviour" into this new text:<br>"The potential mediator role of adherence/dose, the MSEQ, the PASR-12 and the LSNS on the effect of the intervention on physical activity and sedentary behaviour will be explored by covariance analyses,

	adding these scales as covariates to the mixed linear models testing the effect of the intervention. The statistical efficacy analysis of SITLESS has been described previously.[14]”
Tables and figure:<br>-Some of the text is a little bit difficult to read and sometimes gets cut off at the bottom or the top. Likely this will be fixed with publications. Also Figure 1 seems to be cut off at the bottom of the activities and outputs sections. Please revise.	We apologise that these were difficult to read. The tables and figures, especially Figure 1, have been revised to make sure no text is cut and it is easier to read.

Reviewer: 2

Reviewer’s comments	Response
Introduction the research questions need some clarifications:<br>- Page 10, line 28: The research question ‘How does the SMS intervention produce change?’does not specify in what area the authors want to evaluate change. This should be clarified and added to the research question.	With this question we mean behaviour change in terms of increasing physical activity and reducing sedentary behaviour and it has now been clarified in the text of figure 2 since research questions have been deleted from the introduction section to avoid duplication and save words.<br><br>New text:<br>“How does the ERS enhanced by SMS intervention increases physical activity and reduces sedentary behaviour, if applicable?<br>How does each intervention component contribute to producing changes in these behaviours?<br>Does the SITLESS logic model work in practice? Specifically, does the enhanced intervention (ERS enhanced by SMS) work by improving self-efficacy and social support, thus influencing changes in physical activity and sedentary behaviour?<br>Does better adherence to the components of the intervention mediate the reduction of sedentary behaviour and the increase in physical activity?“
Introduction<br>- Page 10, line 30: The second research question ‘How does each intervention component contribute to producing changes in behaviour’ also does not specify what kind of behaviour. Is that only sedentary and physical activity behaviour, or also other kinds of behaviour, social behaviour for example?	With this question we mean behaviour change in terms of increasing physical activity and reducing sedentary behaviour and it has now been clarified in the text of figure 2 since research questions have been deleted from the introduction section to avoid duplication and save words.<br><br>New text:

	“How does the ERS enhanced by SMS intervention increases physical activity and reduces sedentary behaviour, if applicable?<br>How does each intervention component contribute to producing changes in these behaviours?<br>Does the SITLESS logic model work in practice? Specifically, does the enhanced intervention (ERS enhanced by SMS) work by improving self-efficacy and social support, thus influencing changes in physical activity and sedentary behaviour?<br>Does better adherence to the components of the intervention mediate the reduction of sedentary behaviour and the increase in physical activity?“<br>.
Introduction<br>- Page 10, line 32: With regard to the question ‘Does the intervention work by improving selfefficacy and social support?’, do the authors want to evaluate the intervention as a whole or also the components ERS and SMS separately?	Within this question we want to try to determine that mechanism of action through proposed mediation pathways, informed by the underpinning theory. We therefore propose assessing if the SMS intervention enhances the effects of ERS on increasing physical activity and reducing sedentary behaviour by improving self-efficacy and social support. It has been clarified in the text of figure 2 since research questions have been deleted from the introduction section to avoid duplication and save words.<br><br>New text:<br>“How does the ERS enhanced by SMS intervention increases physical activity and reduces sedentary behaviour, if applicable?<br>How does each intervention component contribute to producing changes in these behaviours?<br>Does the SITLESS logic model work in practice? Specifically, does the enhanced intervention (ERS enhanced by SMS) work by improving self-efficacy and social support, thus influencing changes in physical activity and sedentary behaviour?<br>Does better adherence to the components of the intervention mediate the reduction of sedentary behaviour and the increase in physical activity?“<br>.

Introduction<br>- Page 10, line 12: The term PA intervention has not been used previously and I think it has to be changed to ERS.	We thank the reviewer for identifying this error. We have changed PA for ERS in the introduction.
Introduction<br>- Page 10: The bullets for the first two objectives are different than those of the last two objectives. I would suggest to number the aims instead of using bullets, as has been done in the text and in the method section as well.	We agree that numbering aids the readability of the test. We have therefore numbered the aims instead of using bullets in line with the reviewers comments.
Introduction<br>- Page 10, line 39-41: Looking at the other research questions, this font should be in italic.	Research questions are now only in Figure 2 with the same format.
Methods and analysis<br>This study has a lot of aims and accompanying research questions. In the method section, the methods are discussed per aim. However, the aims have multiple research questions. Because the methods	Outcomes of the process evaluation are now explained in the manuscript. A new subsection called “Triangulation with impact evaluation outcomes” and in the figure 3 now called “Research questions, methods adopted and outcomes for the process evaluation of
and outcome measures are not specified per research question it is now difficult to understand which outcome measures are used to answer the research questions. Therefore it is not directly clear how all questions will be answered. I would therefore suggest to specify the outcome measures per research question, within each aim. A table summarizing the methods and outcome measures used for each research question may also help to clarify this.	the SITLESS trial” has been added.
There is little information provided about the intervention itself. This would be very helpful to understand what is exactly being evaluated. For example, how long is the intervention? How long is the follow-up period? How many participants are included in total and per study arm? How often are the specific sessions provided? I understand that the full description of the intervention is described in reference 13 (Giné-Garriga et al. 2017), however for the readability of the article some of this information should also be mentioned here.	We initially did not include details of the intervention as it is presented in detailed in reference 13. However, we acknowledge that it would be helpful for readers to have a summary of the intervention, and this information has been included.<br>New text:<br>“The ERS intervention aims to improve strength, aerobic capacity, and balance during 16 weeks, with two 60-minute sessions per week. The SMS consists of one one-to-one session, six group-based sessions and four telephone prompts to encourage behaviour change. Participants are followed-up for 12 and 18-months post-intervention. The control group receives two sessions consisting of general information and recommendations on healthy ageing. A total of 1,338 participants

	are expected to be included (446 in each arm)."
With regard to the statistical analyses (page 17) some points are unclear:<br>- Line 15-21: Which methods are used to assess differences?	The following explanation has been added to the text: "Percentage of fidelity of the implementation and adherence will be compared across intervention sites to identify whether differences may exist between them. Comparison will be conducted applying analysis of variance models."
With regard to the statistical analyses (page 17)<br>- Line 39-44: The authors mention that the scales "will be analysed within the main database to test their potential role as mediating the effect of the intervention on physical activity and sedentary behaviour." However, it is unclear how this will be analyses. Specify which statistical methods will be used to answer this question.	We have changed the original text "The MSEQ, the PASR-12 and the Lubben Social Network Scale will be analysed within the main database to test their potential role as mediating the effect of the intervention on physical activity and sedentary behaviour" into this new text:<br>"The potential mediator role of adherence/dose, the MSEQ, the PASR-12 and the LSNS on the effect of the intervention on physical activity and sedentary behaviour will be explored by covariance analyses, adding these scales as covariates to the mixed linear models testing the effect of the intervention. The statistical efficacy analysis of SITLESS has been described previously.[14]"
method section:<br>- Page 10, line 53: The abbreviation MRC has already been introduced in the introduction. Introduce the abbreviation once, and then use the abbreviation throughout the rest of the manuscript.	We apologise for this error, and have now corrected this.
method section:<br>- Page 11, line 14: The aim 'Perceived effects' is missing here.	"Perceived effects" have been added New text:<br>"This figure highlights the research question in the categories of implementation, context, mechanisms of impact and perceived effects, and relates them with each methodological procedure applied"
method section:<br>- Page 12, line 28-33: Again the aim regarding 'Perceived effects' is missing here.	"Perceived effects" have been added here also.<br>New text:<br>"The aims regarding context, mechanisms of impact and perceived effects will be explored at the end of intervention and both follow-up periods, whereas implementation is only assessed at the end of the intervention."

method section:<br>- Page 14, line 48-50: Provide information about the psychometric properties of these scales, and in which population this has been studied. This information is needed to understand the suitability of these scales for this study.	We added information in the text on the psychometric properties of the three scales mentioned. New text:<br>"MSEQ has been shown to be a reliable, valid and accurate measure of self-efficacy for physical activity participation in populations with chronic diseases.[21,22] The PASR-12 has been shown to be a reliable and valid scale for the measurement of self-regulation for physical activity among older adults.[19] The LSNS is commonly used as a measure of social networks. It has high levels of internal consistency, stable factor structures, high correlations with criterion variables and clinical cut-points show good convergent validity among European community-dwelling older adults.[23]"
